# Refining pain management in mice by comparing multimodal analgesia and NSAID monotherapy for neurosurgical procedures

**DOI:** 10.1038/s41598-024-69075-2

**Published:** 2024-08-12

**Authors:** Anna Munk, Vanessa Philippi, Verena Buchecker, Marion Bankstahl, Aylina Glasenapp, Andreas Blutke, Effrosyni Michelakaki, Steven Roger Talbot, Jörg Huwyler, Paulin Jirkof, Marcin Kopaczka, Dorit Merhof, Rupert Palme, Heidrun Potschka

**Affiliations:** 1 Institute of Pharmacology, Toxicology, and Pharmacy, Ludwig-Maximilians-Universität München, Koeniginstr. 16, 80539 Munich, Germany; 2 Institute for Laboratory Animal Science, Hannover Medical School, Hanover, Germany; 3 Institute of Veterinary Pathology, Ludwig-Maximilians-Universität München, Munich, Germany; 4 Department of Pharmaceutical Sciences, University of Basel, Basel, Switzerland; 5 Office for Animal Welfare and 3R, University of Zurich, Zurich, Switzerland; 6 Department of Electrical Engineering, RWTH Aachen University, Aachen, Germany; 7 Department of Informatics and Data Science, University of Regensburg, Regensburg, Germany; 8 Department of Biological Sciences and Pathobiology, Experimental Endocrinology, University of Veterinary Medicine, Vienna, Austria

**Keywords:** 3R, Severity assessment, Multimodal analgesia, Craniotomy, Postsurgical pain, Neuroscience, Pharmacology, Animal behaviour, Pain management, Neurosurgery, Mouse

## Abstract

While neurosurgical interventions are frequently used in laboratory mice, refinement efforts to optimize analgesic management based on multimodal approaches appear to be rather limited. Therefore, we compared the efficacy and tolerability of combinations of the non-steroidal anti-inflammatory drug carprofen, a sustained-release formulation of the opioid buprenorphine, and the local anesthetic bupivacaine with carprofen monotherapy. Female and male C57BL/6J mice were subjected to isoflurane anesthesia and an intracranial electrode implant procedure. Given the multidimensional nature of postsurgical pain and distress, various physiological, behavioral, and biochemical parameters were applied for their assessment. The analysis revealed alterations in Neuro scores, home cage locomotion, body weight, nest building, mouse grimace scales, and fecal corticosterone metabolites. A composite measure scheme allowed the allocation of individual mice to severity classes. The comparison between groups failed to indicate the superiority of multimodal regimens over high-dose NSAID monotherapy. In conclusion, our findings confirmed the informative value of various parameters for assessment of pain and distress following neurosurgical procedures in mice. While all drug regimens were well tolerated in control mice, our data suggest that the total drug load should be carefully considered for perioperative management. Future studies would be of interest to assess potential synergies of drug combinations with lower doses of carprofen.

## Introduction

In neurobiological research, intracranial interventions in mice are frequently applied surgical procedures^1,2^. However, the status quo of analgesics used for craniotomies in laboratory rodents raises concerns about the efficacy of the respective pain management. Recent evidence from a systematic review shows that 75% of studies published in 2019 did not report the use of perioperative analgesia^1^. Only 5.7% of studies reported using two or more analgesic components in 2019, indicating an untapped potential for multimodal analgesia^1^. In animal-based research, compliance with the 3R principles, including the adequate use of analgesia and anesthesia, are legal requirements within the European Union (Directive 2010/63/EU). In mice, craniotomy-associated pain lasts up to 48 h^2^, which is similar to reports of human patients suffering from moderate to severe pain for up to two days after neurocranial surgery^3^. Moreover, evidence exists that under-treatment of postsurgical pain can increase the variance of numerous readout parameters and can thus contribute to the general issue of reproducibility and robustness in animal-based research^4–6^.

As information on the pharmacokinetics and -dynamics of analgesics in mice is limited, therapeutic plasma concentrations are often extrapolated from other species and dose recommendations are often based on general experience^7,8^. Therefore, several recent studies suggested that recommended dosages of analgesic drugs in laboratory mice might lack sufficient efficacy^9–11^, and shorter administration intervals might be necessary to sustain therapeutic plasma levels^12,13^. Cho and colleagues (2019) reported that buprenorphine as well as high dosages of carprofen and meloxicam are effective in reducing craniotomy-associated pain^2^. To our knowledge, no study reports data on multimodal analgesia in murine craniotomies. In multimodal analgesia, analgesics with different, complementary mechanisms of action and suitable pharmacokinetics are combined to benefit from synergistic, analgesic effects. Considering their different target sites in the nociceptive system^6^, we selected NSAIDs, opioids, and local anesthetics as drug classes for this study. A single subcutaneous administration of the most commonly applied opioid, buprenorphine, however, fails to maintain therapeutic plasma levels for longer than 4 to 8 h^12–14^. To avoid high peak concentrations and to minimize stress due to frequent injections^15^, we decided to administer a sustained-release buprenorphine formulation (BUP-Depot), which provided sufficient pain relief up to 72 h in earlier mouse studies^16,17^. Because the NSAID carprofen proved ineffective at lower dosages^9,18,19^, Glasenapp and colleagues (2023) investigated the pharmacokinetics of high-dose carprofen administered via drinking water^20^. Based on their findings, the respective dosing scheme was applied in our study.

Pain assessment is not only a prerequisite for assessing severity and determining humane endpoints, but is also of utmost importance for successful perioperative pain management^8,21^. In this context, a clear distinction must be made between the terms ‘nociception’ on the one hand, and ‘pain’ on the other. Nociceptive assays cannot capture the complexity of postsurgical pain, as it is a conscious experience with various consequences including an impact on the animal’s affective state^6,7^. Moreover, as prey animals, mice hide pain and impairments to avoid the attention of predators^22^. To overcome the challenge of pain assessment in mice, composite measure schemes comprising physiological, biochemical, and behavioral parameters need to be applied^22,23^. In recent years, the mouse grimace scale (MGS), assessing facial expression patterns reflecting the experience of pain^24^, has been frequently used to measure postsurgical pain^21^.

Therefore, this study aimed to investigate the tolerability and efficacy of multimodal analgesic regimens and to refine perioperative pain management in murine craniotomies. We postulated that multimodal analgesic regimens are more effective in reducing craniotomy-associated pain than a monotherapeutic approach. With this study, we also aimed to validate sensitive severity assessment parameters for their applicability in quantifying postsurgical pain.

## Results

**Figure 1:**
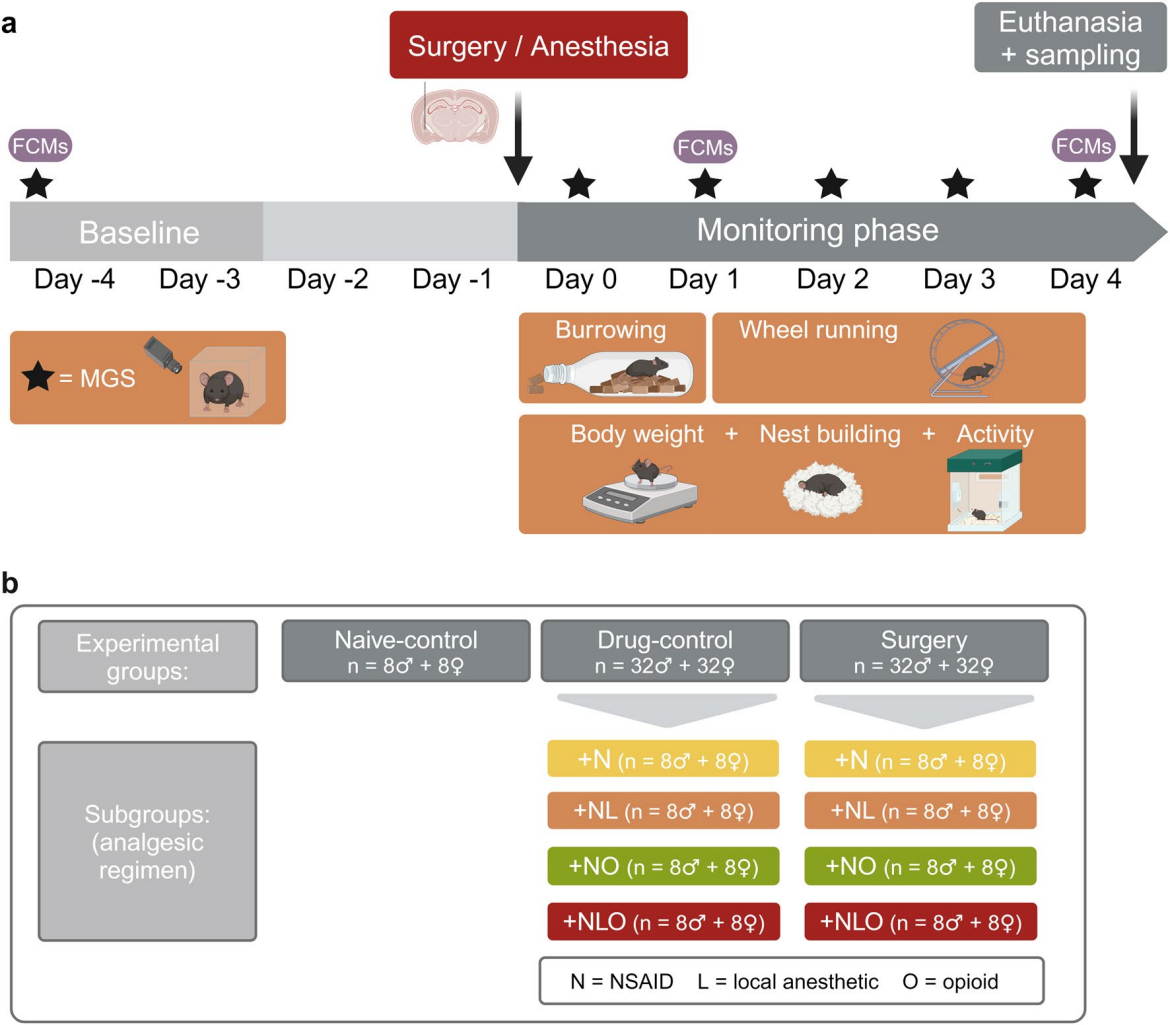
Experimental design. The study design including the test battery with (patho)physiological, biochemical, and behavioral parameters is illustrated in (**a**). The experimental groups and different subgroups are presented as an overview in (**b**). (MGS = mouse grimace scale, FCMs = fecal corticosterone metabolites, Created with BioRender.com).

Before starting the main study, we conducted a pilot study to assess the tolerability and efficacy of oral carprofen administration (see supplementary (S) information and Table S1). Based on a test battery comprising (patho)physiological, biochemical, and behavioral parameters (Fig. 1a), we assessed the tolerability and efficacy of four different analgesic regimens in the main study. Mice of the surgery group (referred to as ‘surgery mice’) and of the drug-control group (‘drug-control mice’) were assigned to the following subgroups + N, + NL, + NO, and + NLO (N = NSAID, L = local anesthetic, O = opioid; Fig. 1b). The influence of the factors analgesic regimen, time and their interaction on the read-out parameters was statistically analyzed. Drug-control and surgery subgroups were subsequently compared to the respective naive-control groups (‘naive-control mice’) using post hoc tests. Further details on the statistical analyses of significant results can be found in the supplementary information (Table S3-S8, S10, S17).

### Mouse grimace scale (MGS)

**Figure 2:**
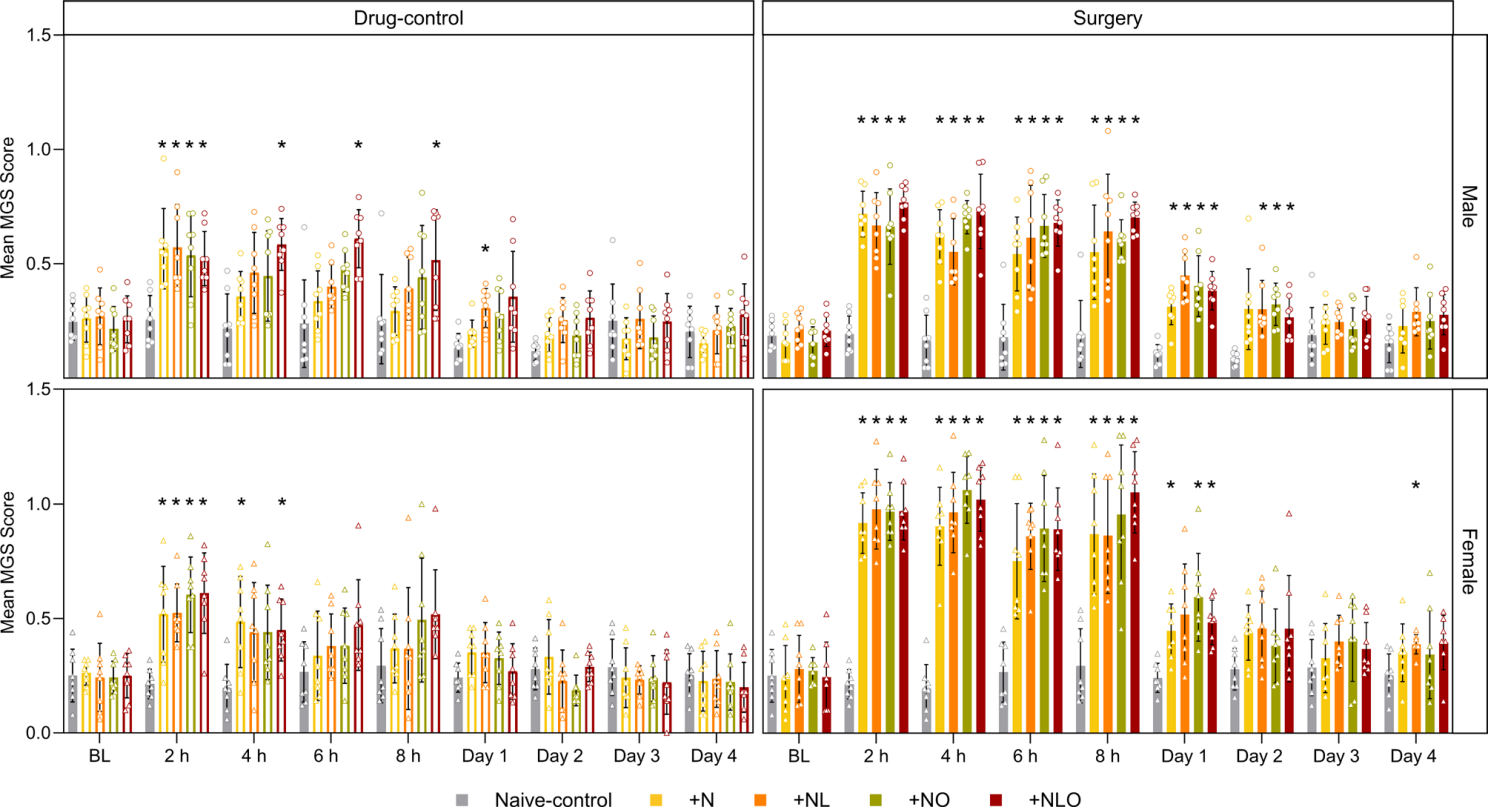
MGS. The mean MGS at baseline and after the intervention are illustrated for the drug-control and surgery groups compared to naive-control groups in both sexes (group sizes differing from n = 8: males 4 h surgery + NL n = 7, day 4 surgery + N n = 7). The MGS was significantly increased in the first hours until postsurgical day 1 in female and until day 2 in male mice. No clear group differences were found between the analgesic regimens. The MGS of drug-control mice was transiently increased after anesthesia. This effect was detectable in the male drug-control mice of the + NLO subgroup over a longer period of time. (* = p < 0.05; mean ± SD; Two-way RM ANOVA/ Mixed effects model with Bonferroni post hoc test; BL = baseline).

The main read out parameter MGS can be regarded as an indicator for postsurgical pain and was therefore closely monitored after anesthesia and surgery. 2 h after anesthesia, the MGS of drug-control mice was transiently increased in all analgesia subgroups in both sexes. This increase was prolonged in male + NLO drug-control mice 4 h and 6 h after anesthesia and on day 1 in male + NL drug-control mice. In female drug-control mice, the MGS remained significantly increased 4 h after anesthesia in + N and + NLO subgroups compared to respective naive-control mice. Regardless of the analgesic regimen, the MGS was significantly higher in surgery mice of both sexes than in naive-control mice on day 0. In male mice, the increase in MGS persisted until day 2, with the + N subgroup initially reaching naive-control levels. MG scores were increased in female mice until postsurgical day 1 for + N, + NO, and + NLO subgroups compared to naive-control mice. A further increase in MG scores was identified on day 4 for female + NL surgery mice compared to naive-control mice (Fig. 2, Table S3). Graphical illustrations of the sum MG-scores and information on the assessability of action units are included in the supplementary information (Fig. S1, S2, Table S2, S3).

### Home cage-based behavioral assessment

The general activity of the mice in their home cage and in the running wheel (see supplementary information) and the performance in non-essential behaviors, such as nest building and burrowing, were evaluated to detect craniotomy-associated postsurgical impairments and compound-related adverse effects.

#### Activity

**Figure 3:**
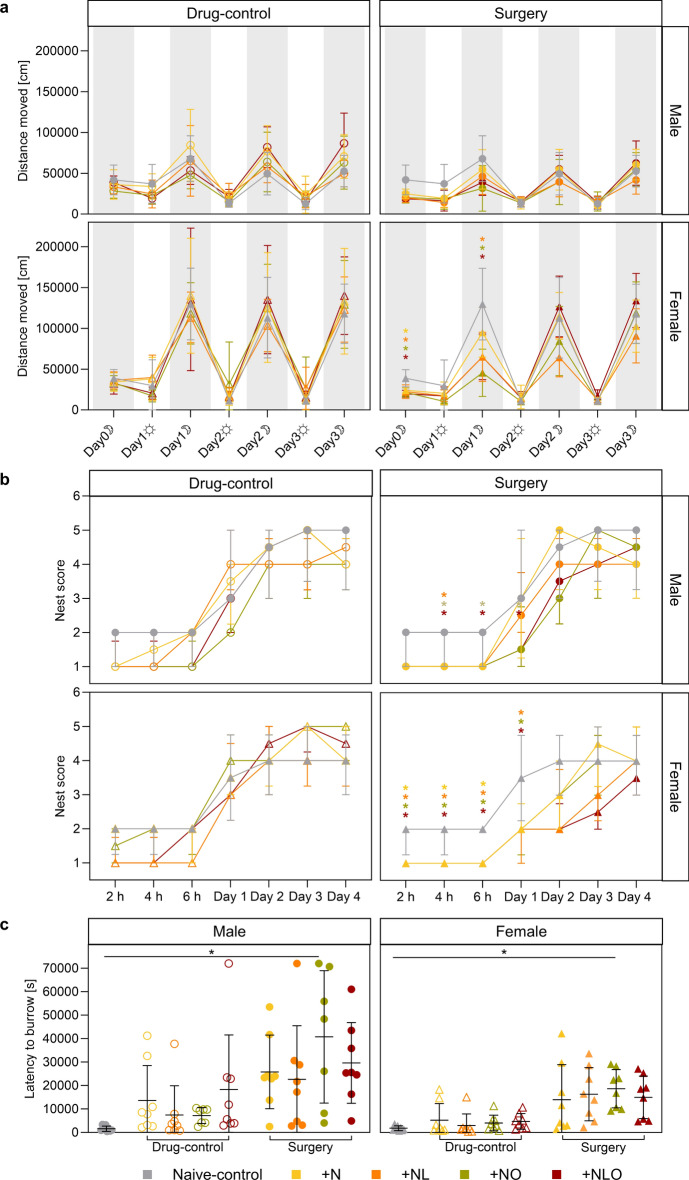
Home cage-based behavioral assessment. The graph (**a**) shows (the distance moved by the mice in the PhenoTyper home cages during the experimental phase for each 12-h of light phases (☼) and dark phases (☽) (group sizes differing from n = 8: males day 2 light/dark and day 3 light surgery + N n = 7, day 4 light/dark surgery + N n = 6 and all other groups n = 7). All female surgery mice moved a significantly shorter distance in the dark phase on day 0, regardless of the analgesic regimen. Distance moved was still significantly impaired during the dark phase on day 1 in the female mice of the + NL, + NO, and + NLO subgroups. The nest scores during the experimental phase are illustrated in (**b**) (group sizes differing from n = 8: males day 1 drug-control + NO n = 7, day 4 surgery + N n = 7). The nest complexity was significantly affected in both sexes in the first hours after surgery. Nest complexity of the mice in the surgery + N subgroup was not affected at all in the males, whereas it increased in the females on the first day. The latency to initiate burrowing within 20 h (day 0-1) is shown in (**c**) (group sizes differing from n = 8: males drug-control and surgery + NO n = 7). Both male and female mice in the surgery + NO subgroup showed a significantly increased latency to the onset of burrowing activity after surgery. (* = p < 0.05, analgesic regimen color-coded in (**a**) and (**b**); mean ± SD in (**a**) and (**c**), median ± IQR in (**b**); Two-way RM ANOVA/ Mixed effects model with Bonferroni post hoc test in (**a**), Kruskal-Wallis Test with Dunns post hoc test in (**b**), One-way ANOVA with Bonferroni post hoc test in (**c**)).

The activity of drug-control mice was not affected by the analgesic regimens or the anesthesia intervention within the first 20 h after anesthesia and the entire experimental phase. Regardless of the analgesic subgroup, male and female surgery mice showed a significantly reduced distance moved within the first 20 h after surgery compared to naive-control mice (Fig. S3, Table S4). The mixed effect analysis of distance moved during the entire experimental phase in male surgery mice could not identify any significant differences between analgesic regimens. In female surgery mice, the distance moved was significantly decreased in all subgroups during day 0 in the dark phase and in + NL, + NO, and + NLO subgroups during day 1 in the dark phase (Fig. 3a, Table S4).

#### Nest building

Neither anesthesia nor the different analgesic regimens exerted significant effects on the nest complexity observed for drug-control mice. In the first six hours after surgery, male and female mice showed a significant decrease in nest complexity scores when compared to naive-control mice. Male + NL, + NO, + NLO surgery mice and female surgery mice of all subgroups reached lower nest scores than respective naive controls. The nest building activity of female mice was still impacted on postsurgical day 1, with + NL, + NO, and + NLO mice reaching lower nest scores than sex-matched naive-control mice (Fig. 3b, Table S5).

#### Burrowing

Three surgery mice and one drug-control male mouse did not display any burrowing activity after surgery or anesthesia. Therefore, their burrowing latency was set to 72,000 s, resembling the total duration of observation. Burrowing latency of drug-control mice was not affected by anesthesia or the different analgesic regimens (Fig. 3c, Fig. S4, Table S6). In surgery mice treated with + NO, the burrowing latency was significantly increased (Fig. 3c, Table S6). However, when considering the baseline burrowing performance with the delta-burrowing latency, the previous findings were confirmed for female + NO surgery mice only (Fig. S4, Table S6).

### Body weight

**Figure 4:**
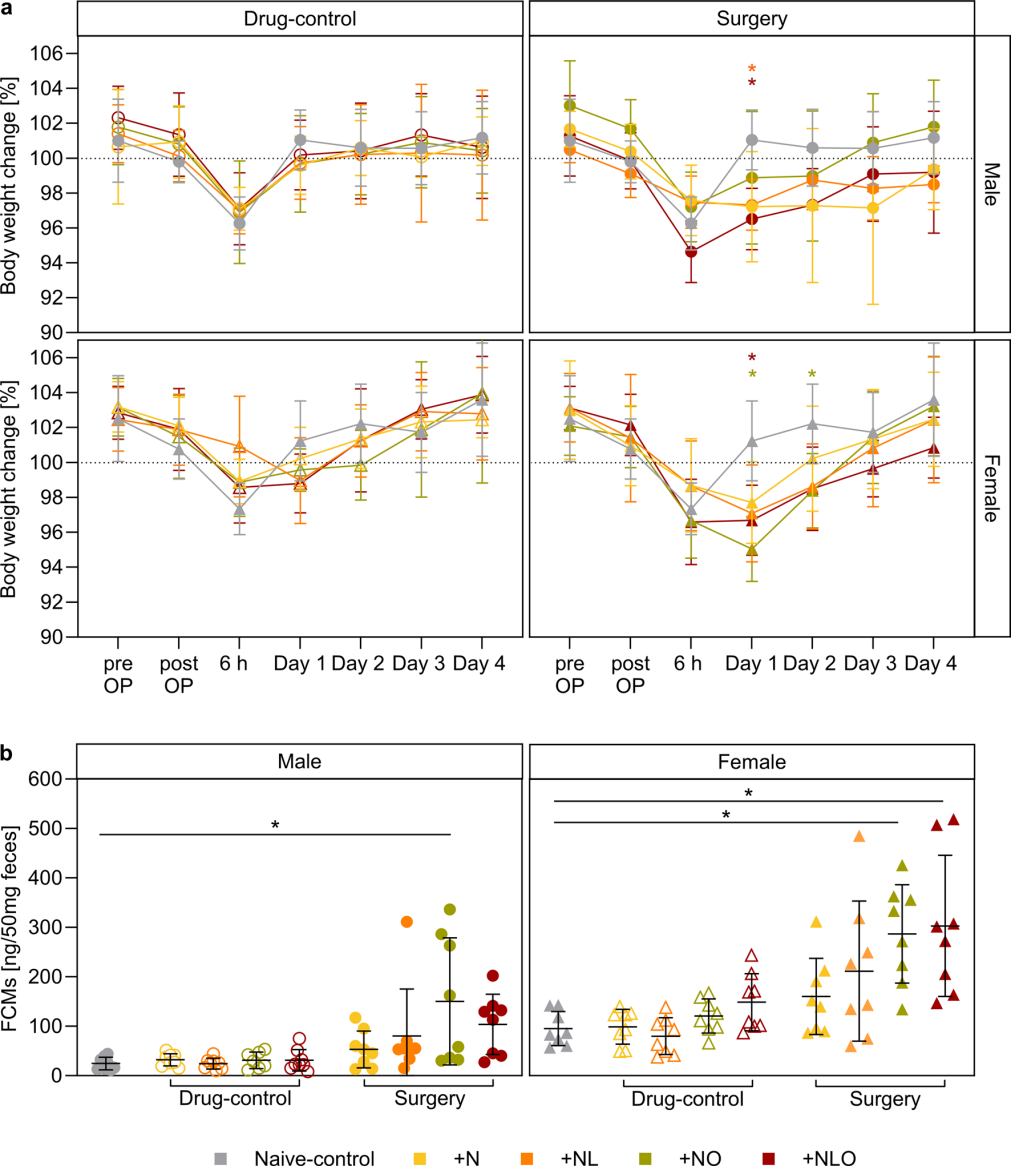
BWC and FCMs. The percentage change in body weight compared to the mean baseline values is illustrated in (**a**) (group sizes differing from n = 8: males day 4 surgery + N n = 7; females: 6 h drug-control + NL n = 7). A significant decrease in body weight change was identified for male surgery + NL, + NLO, and female surgery + NO, + NLO mice on day 1 and female surgery + NO mice on day 2. The concentration of fecal corticosterone metabolites on day 1 (**b**) was significantly increased in male surgery + NO and female surgery + NO, + NLO mice (group sizes differing from n = 8: males day 1 drug-control + NO n = 7, day 4 surgery + N n = 7; females day -6 surgery and drug-control + N n = 7). (* = p < 0.05, analgesic regimen color-coded in (**a**); mean ± SD in (**a**) and (**b**); Two-way RM ANOVA in (**a**), One-way ANOVA in (**b**) with Bonferroni post hoc test in (**a**) and (**b**); OP = operation).

The body weight was measured daily following surgery or anesthesia and the percentage of body weight change relative to individual baseline mean values was calculated. Neither the anesthesia nor the different analgesic regimens substantially affected the body weight change of drug-control mice of both sexes compared to respective naive-controls. Depending on the analgesic regimen, the body weight change was partially affected in surgery mice of both sexes after the surgical intervention. Compared to naive-control mice, the body weight change was significantly decreased in male surgery mice of the + NL and + NLO subgroups on day 1. For female surgery mice, the analysis revealed a significant reduction in body weight change of the + NO subgroup on days 1 and 2 and the + NLO subgroup on day 1 in comparison to female naive-control mice (Fig. 4a, Table S7).

### Fecal corticosterone metabolites (FCMs)

Fecal samples were collected repeatedly during the study to measure stress hormone metabolite levels. The FCM concentrations of female drug-control mice were affected by analgesic treatment. However, significant differences between naive-control and specific subgroups could not be confirmed in post hoc multiple comparison tests. The concentration of FCMs was significantly increased on postsurgical day 1 in male surgery mice of the subgroup + NO and female surgery mice of the subgroups + NO and + NLO (Fig. 4b, Table S8). The analysis of FCM concentrations at baseline, day 1 in male drug-control mice, and day 4 did not reveal any differences related to the analgesic regimen (Fig. 4b, Fig. S5).

### Neuro score

**Figure 5:**
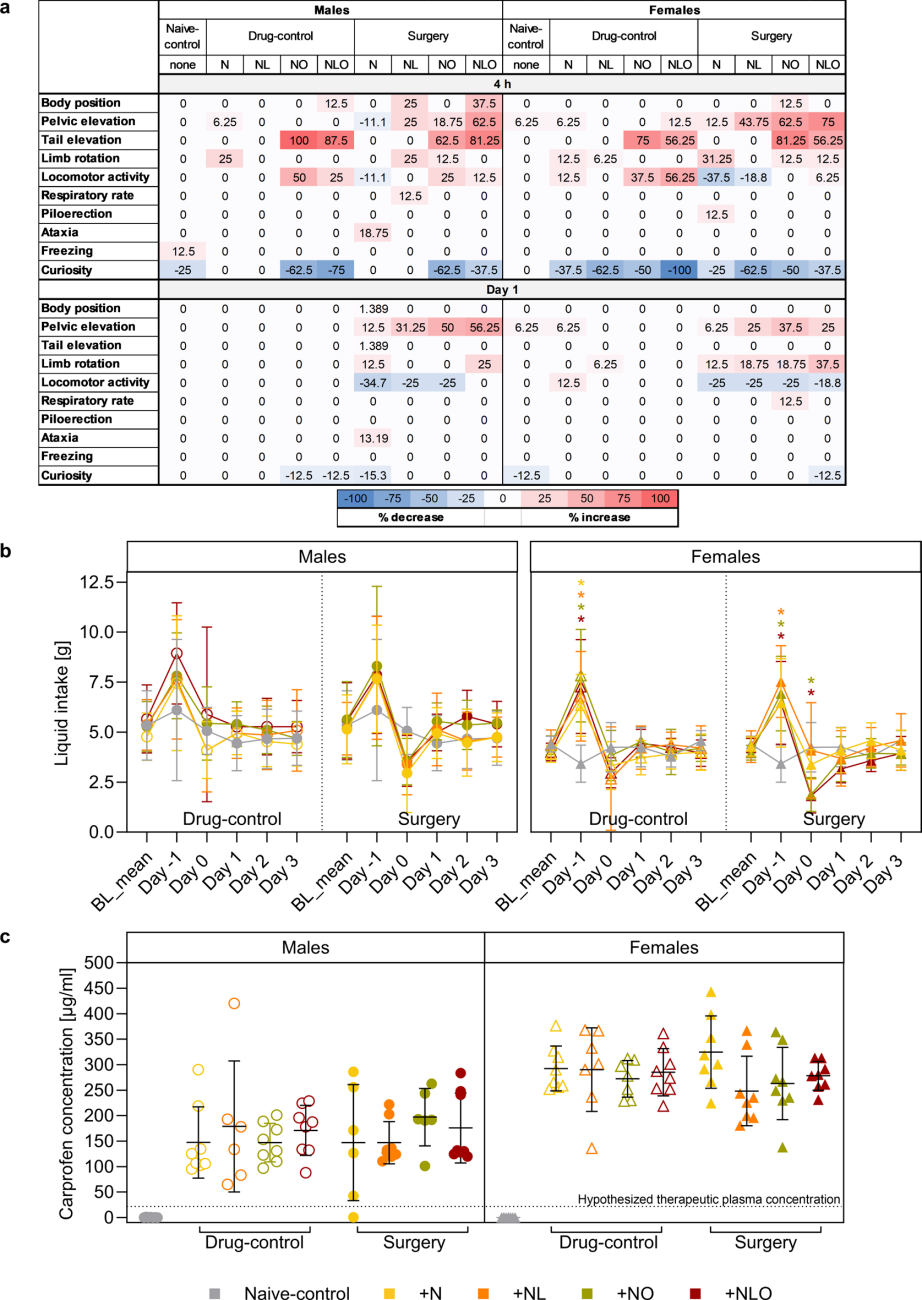
Neuro score, liquid intake, and carprofen plasma concentration. Relevant Neuro score parameters at the 4 h time point and on day 1 are shown as percentage mean increase (highlighted in red) or decrease (highlighted in blue) for individual scores per sex, time point, and analgesic regimen in (**a**). While the liquid intake (**b**) significantly increased in most female mice on day -1, it showed a decline on day 0 for female surgery + NO and + NLO mice. Data shown for BL_mean represents the individual, mean water intake over five days during baseline measurements (group sizes differing from n = 8: males day 4 surgery + N n = 7; females day 0 drug-control + NL n = 7). The carprofen plasma concentrations measured on day 4 (**c**) exceeded the hypothesized therapeutic plasma level of 20–24 µg/ml in both male and female mice (group sizes differing from n = 8: males naive-control n = 7, surgery + N n = 7, drug-control + NL n = 7, surgery + NO n = 6; females: naive-control n = 7, drug-control + NL n = 7, drug-control + NO n = 7). (* = p < 0.05, analgesic regimen color-coded in (**b**); mean ± SD in (**b**) and (**c**); Two-way RM ANOVA with Bonferroni post hoc in (**b**)).

Some Neuro score parameters (vocalization while handling, touch reaction, irritability, urination, and defecation) showed individual variance at baseline and subsequent time points. Four hours after anesthesia or surgery, the majority of male and female mice of the + NO and + NLO subgroups presented with elevated tails, many male mice showed less curiosity behavior, and some drug-control mice stood out with hyperlocomotion. On the following postoperative days, scores for locomotor-related parameters, such as pelvic elevation, limb rotation, and locomotor activity, were influenced to varying degrees in all surgery subgroups and both sexes (Fig. 5a, Table S9).

### Liquid intake

The liquid intake was measured daily, first, to obtain baseline water intake values for determining the concentration of carprofen in the drinking water, and second, to assess the postsurgical oral uptake of the analgesic compound. The baseline water intake measurements accounted for a daily mean water intake of 5.324 g (standard deviation (SD) = 1.509) in males and 4.201 g (SD = 0.525) in females. The liquid intake during the experimental phase changed over time in male mice, but neither the analgesic regimen nor the impact of the intervention significantly changed the liquid intake compared to naive-control mice. However, in female drug-control mice and in all surgery mice except for the + N subgroup, the liquid intake was significantly increased on day -1, when mice received carprofen-treated water for the first time. The liquid intake was significantly reduced in female + NO and + NLO surgery mice on day 0 (Fig. 5b, Table S10).

### Carprofen plasma concentration

The liquid chromatography-mass spectrometry of plasma samples taken at euthanasia after five consecutive days of carprofen administration via the drinking water revealed carprofen concentrations of mean (M) = 162.8 µg/ml (SD = 70.98) in males and of M = 281.9 µg/ml (SD = 59.31) in females (Fig. 5c). The measured plasma concentrations exceeded hypothesized therapeutic carprofen plasma concentrations of 20–24 µg/ml^7,20,25^ by a factor of 6 to 11.

### Histopathology

**Figure 6:**
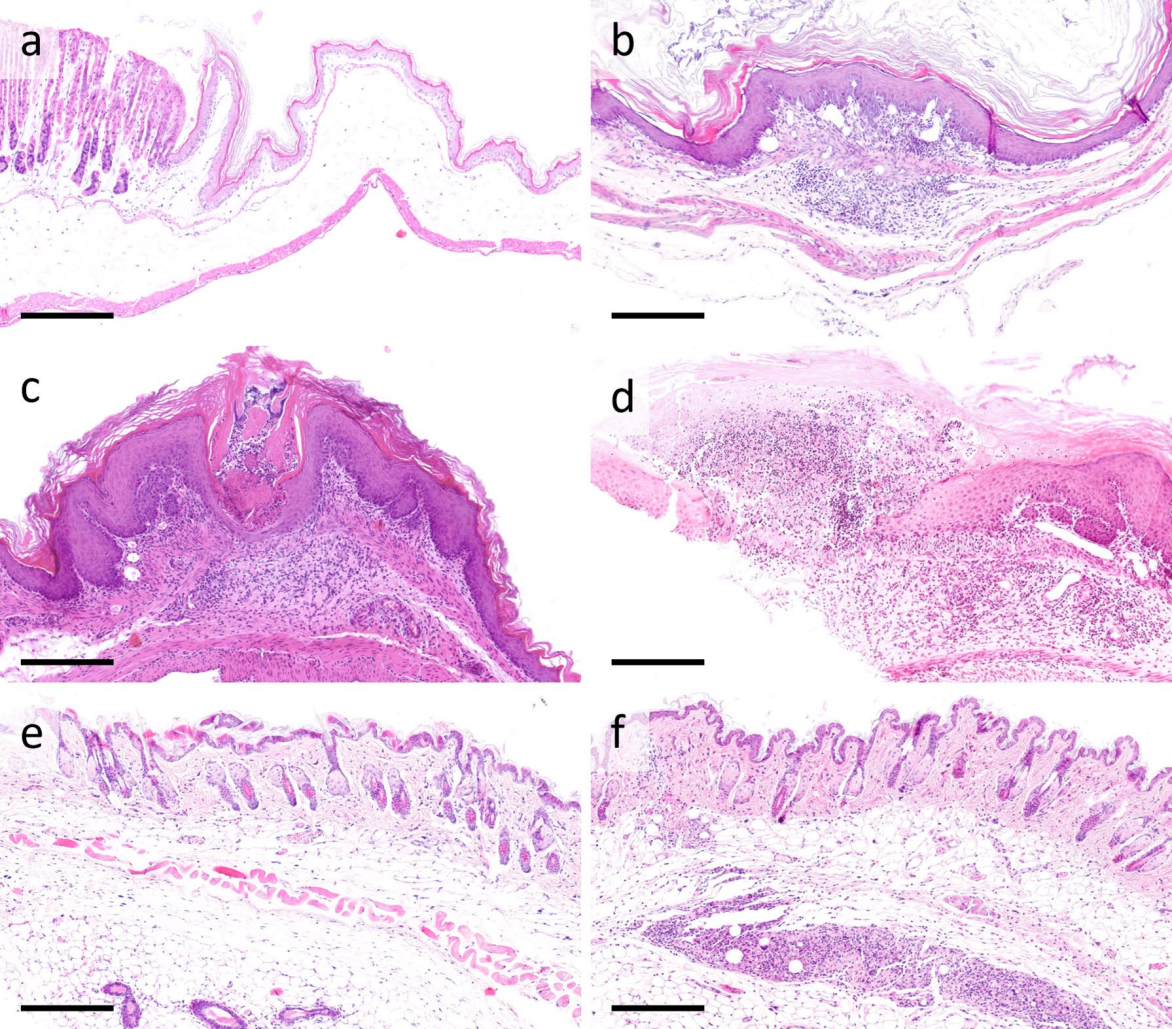
Histopathology. Representative histological images of the lesions within the gastric mucosa (**a**–**d**) and haired skin (**e**,**f**). Histopathological lesions of the gastric mucosa were present in 22% of the samples, without correlating to the treatment group or sex. The spectrum of histopathological findings ranged from no alterations (**a**) to moderate, focal, mixed cellular gastritis, covered by intact gastric mucosa (10.5%) (**b**), focal erosions of the epithelium (9%), accompanied by mixed intraepithelial (microabscess) and mucosal inflammation, granulation tissue formation and hyperplasia of the intact epithelium (**c**), to ulcerative lesion with associated inflammation (2%) (**d**) (example cases: (**a**): naive-control, male; (**b**): surgery + N, female; (**c**): drug-control + NL, male; (**d**): drug-control + NO, male. Please note that the histopathological lesions shown here are not representative for distinct experimental treatment groups.). Skin lesions were frequently present in mice that were subcutaneously injected with BUP-Depot (69%). Unaltered skin of a female naive-control mouse (**e**). Focally extensive, mild to moderate, mixed cellular panniculitis, in the injection site of a drug-control + NLO mouse (**f**). (FFPE, Hematoxylin and eosin (HE); Scale bars = 250 μm).

Most sections of gastrointestinal tissue did not display relevant histopathological lesions (Fig. 6, Table S11). In the stomach, lesions were noticed in a limited number of cases (22%) without an apparent correlation to a distinct experimental group or the sex of the animals. The pathological alterations detected in the gastric mucosa, were almost entirely limited to the non-glandular part of the gastric mucosa and were predominantly localized in proximity to the transition from the non-glandular to the glandular part of the mucosa. The lesions comprised mild to moderate, focal, mixed infiltrations of inflammatory cells in the mucosa and submucosa (10.5%) and focal intraepithelial micro-abscesses, sometimes accompanied by focal formation of granulation tissue, as well as focal erosions (9%) or ulcerations (2%) of the overlying mucosa. In naive-control mice no such lesions were observed. Apart from focal, minimal erosions in two female mice (1.4%), no apparent pathological lesions were observed in the duodenal mucosa. Observed skin lesions comprised mild to moderate, focally extensive, mixed cellular panniculitis, frequently seen in mice receiving subcutaneous BUP-Depot injections (69%). Examined sections of skin samples from untreated (i.e., not-injected) mice did not show histopathological alterations.

### Design of a composite measure scheme (CMS)

**Figure 7:**
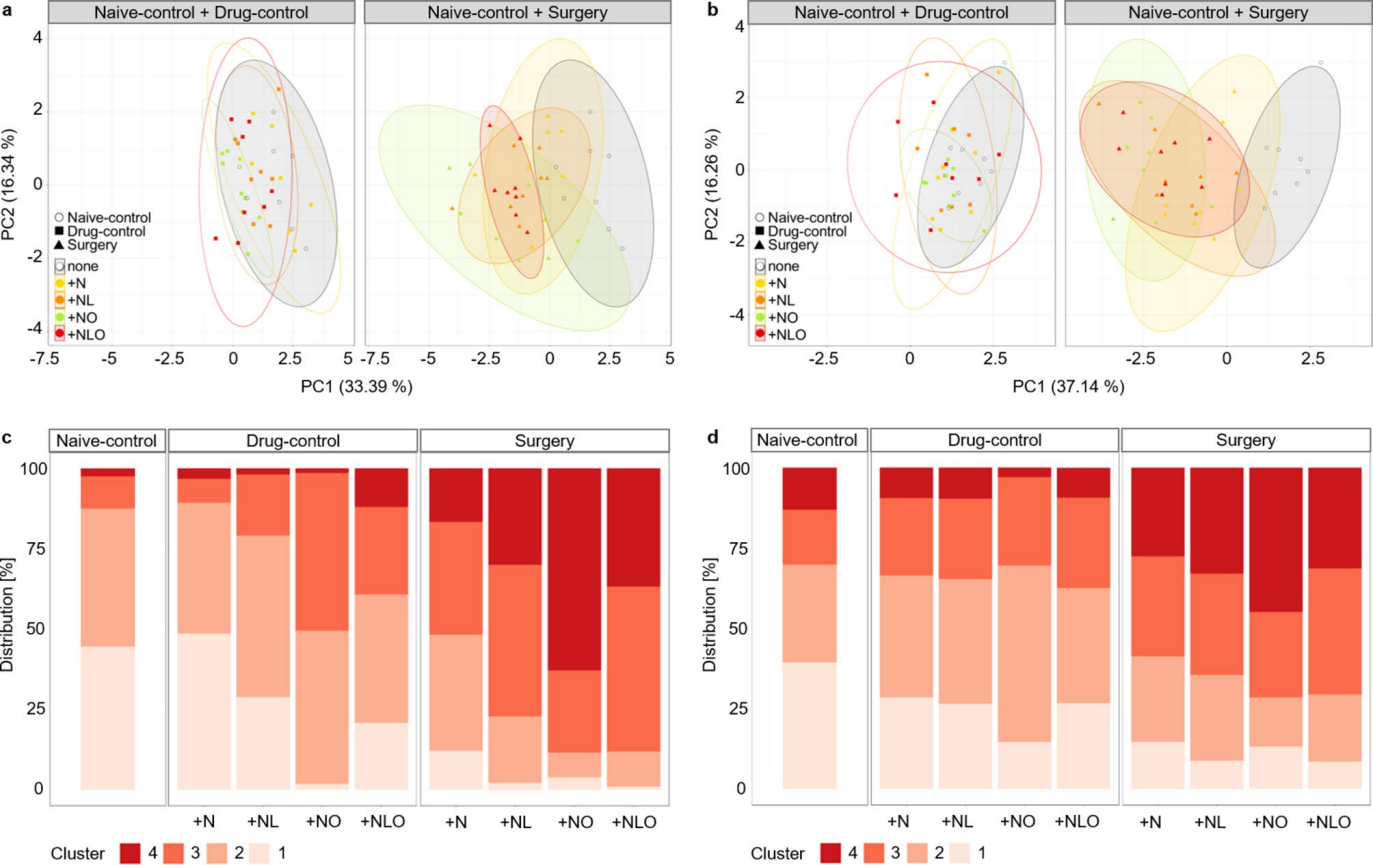
PCA and CMS. Principle component analyses of selected parameters on day 1 are illustrated for males (**a**) and females (**b**). In both sexes, a separation of naive-control and surgery groups along PC1 became evident, whereas data sets of naive-control and drug-control groups largely overlap. The allocation of male (**c**) and female (**d**) mice to predefined clusters by *k*-*means*-based clustering is shown for selected parameters on day 1 (cluster 4 = high severity, cluster 1 = low severity). The surgery + NO group had the highest proportion of mice in severity cluster 4 in both sexes, while the surgery + N group had the lowest proportion of mice in severity cluster 4.

A previously described bioinformatic workflow^26,27^ was applied to design a composite measure scheme for comparative severity assessment of electrode-implanted mice under different analgesic regimens. Since single parameters were mainly affected on the first postsurgical day and analyses revealed only minor persistent changes during the following days, we focused only on the first postsurgical day. First, eleven parameters, which provided additive information for severity assessment, were preselected for both sexes (Table S12). To identify correlated parameters (defined as: correlation coefficient r < -0.5 or > 0.5 in combination with p < 0.05), a Spearman correlation analysis was performed for each sex (Table S13). The results of the correlation analysis are illustrated in a heat map (Fig. S6). High correlations (r < -0.7 or > 0.7 and p < 0.05) between distance moved dark/light phase, velocity dark/light phase and VWR dark phase (r = 0.78 to 0.99, all correlations p < 0.001), lead to the exclusion of the parameters velocity dark/light phase and VWR dark phase in both male and female mice with eight parameters remaining for further analysis. Data of male and female mice were then subjected to a principle component analysis. PC 1 accounted for 33.39% (SD = 1.14) and 37.14% (SD = 1.72) and PC 2 for 16.34% (SD = 1.09) and 16.26% (SD = 1.14) of the total variance of data from male and female mice, respectively. To improve visualization, results of one PC analysis per sex are illustrated separately for naive-control and drug-control or surgery groups in Fig. 7a,b. PC 1 and 2 failed to separate data from naive-control and drug-control mice of both sexes, whereas data from both sexes suggest a separation between naive-control and surgery mice along PC 1. However, a clear separation along the two plotted main principle components for the different subgroups of the surgery mice could not be detected.

For the robust identification of top-ranking parameters, as described previously^26^, the data sets were subjected to 100-fold PCA runs, each resampling 80% of the data set along PC 1 and PC 2. For male mice, the following parameters were mainly responsible for the variance within the dataset and repeatedly occurred within the first 4 positions: nest building, body weight change, MGS, FCM, and distance moved during the light phase. The following top parameters were identified for female mice: Neuro score, distance moved during the light phase, FCM, body weight change, and nest building (Table S14). All eight parameters appeared within the top-ranking parameters, although some accounted for only a small proportion of variance. To avoid loss of informative value of these parameters, all parameters were included in creating the composite score as has been proposed for murine datasets before^27^. The number of clusters was selected upon interpreting the scree plot within the cluster sum of squares along PC 1 and PC 2 (Fig. S7). 100-fold *k*-*means*-based clustering was performed by resampling 80% of the dataset along PC 1 and PC 2 each time^26^ to determine the cluster thresholds of 4 selected clusters. Mice of different subgroups were allocated to one of the 4 clusters (Fig. 7c,d). Cluster 4 was defined to represent high severity. Cluster 3, 2 and 1 represented gradually decreasing severity levels. In male mice allocation to cluster 4 was highest in the + NO surgery group (62.8%) followed by mice in + NLO (36.7%), + NL (30.0%) and + N (16.6%) surgery subgroups. Similar proportions of male naive-control and drug-control mice were allocated to cluster 4 (range: 1.4 – 12%). However, in cluster 3, differences between + NO (49.0%) and + NLO (27.1%) in comparison to + NL (19.0%), + N (7.4%) drug-control mice and naive-control mice (10.0%) are observed. The percentage of female surgery groups allocated to the highest severity cluster 4 is as follows: + NO (44.9%), + NL (32.9%), + NLO (31.4%), and + N (27.5%). The cluster allocation of female drug-control mice seems similar to the naive-control mice apart from a slight difference in cluster 2 of the + NO group (54.9% versus 30.4% in naive control mice) (Table S15, S16).

## Discussion

This study investigated the tolerability and efficacy of different analgesic regimens in mice undergoing a neurosurgical procedure. The experimental findings also provide information on the validity of different parameters for postsurgical severity assessment in mice. In addition, we applied and further validated a bioinformatic workflow that was previously developed for the design of composite measure schemes allowing evidence-based comparative severity assessment.

The MGS has been thoroughly validated as a readout parameter responding to different noxious stimuli^9,24,28^. While the sensitivity of the MGS has been repeatedly confirmed (reviewed by^21,28–30^), the MGS responds to other influencing factors and its increase can also serve as a general illness symptom^31–34^. The current study further confirms these limitations in the specificity of the MGS. In mice only exposed to isoflurane anesthesia in combination with different analgesia regimens, the MG scores proved to be significantly increased during the early period following anesthesia. These findings are in line with earlier reports describing an impact of isoflurane exposure on the MGS in CD1, DBA/2 and female C57BL/6JRj mice^9,35,36^. The prolonged increase that we observed in mice with buprenorphine injection might indicate that this opioid can exert effects on the MGS. However, careful interpretation is necessary considering earlier findings arguing against a relevant impact of buprenorphine^9,35^ and considering that mice in our study were exposed to a combination of analgesics.

Evidence exists that locomotor activity, circadian rhythmicity, wheel running activity, nest building and burrowing can serve as valuable parameters for evidence-based pain assessment in mice^18,19,21,22,37,38^. In our study, home cage activity patterns including distance moved, wheel running activity, nest building, and initiation of burrowing activity remained unaffected by drug exposure regardless of the drug class and their combinations. The reductions in dark phase activity and nest complexity observed in the different treatment groups during the early postsurgical phase can thus be interpreted as an indicator of residual pain and distress without the need to correct for drug effects. The same conclusion seems valid for the latency to initiate burrowing behavior following the surgical intervention. However, for this readout parameter a high variance was observed, which, on the one hand, might reflect the individual condition of the animals. On the other hand, it seems to result in a low sensitivity of the parameter for detecting group differences. In this context, it is of interest that a high variance in burrowing performance was also evident in earlier studies assessing its value as a general severity assessment or pain parameter^18,37,39–41^.

As previously discussed, body weight is a parameter that can be easily determined but might require complex considerations for interpretation^38,42,43^. Our data from naive mice reveal a drop in body weight as a non-specific response to transport and an approximately four to ten-hour stay in the laboratory environment even though the animals remained in their home cage and had continuous ad libitum access to their food pellets. While data from the surgical day need to be corrected for this non-specific effect related to the disruption of the stay in the animal facility, a delay in recovery of normal body weight following surgery seems to reflect residual pain and distress in some treatment groups. Thus, in line with earlier studies in laboratory mice^44,45^, our data confirm the value of postoperative body weight monitoring for severity assessment.

Analysis of FCMs represents a well-established indirect non-invasive approach for assessment of HPA (hypothalamic–pituitary–adrenal) axis activation and glucocorticoid release^46^. While FCM increases must be considered as a non-specific measure of stress responses^46^, elevated FCM levels can be observed as a consequence of non-controlled or insufficiently controlled pain in laboratory rodents^11,37,38,42^. Our findings revealed an increase in FCM levels only in those surgical groups that were exposed to the opioid buprenorphine. A study in opioid-naive rodents already described a stimulation of corticosterone release in response to acute administration of various endogenous opioid peptides suggesting that different opioid receptors can modulate the HPA axis^47^. Thus, opioid exposure should be considered as a potential confounding factor for the interpretation of FCM levels in the context of pain assessment. However, in our study, opioid exposure without a surgical procedure did not exert relevant effects on FCM levels indicating a probable mixed effect of surgery and buprenorphine exposure.

Even with complete elimination of pain, the tissue injury caused by the surgical intervention activates the nociceptive system leading to a complex pathophysiological surgical stress response involving the sympathetic nervous system, the HPA axis, changes in the metabolism and the immune system^6,48^. This surgical stress response might have impacted results of several read-out parameters of this study. Moreover, interpretation of all findings needs to consider that since we did not include a surgical group without analgesic drug treatment for ethical reasons, some of the alterations could be related to a mixed effect, i.e. an interaction between postsurgical pain and distress and drug adverse effects. For instance, animals with pain and distress might be more sensitive to specific adverse effects than naive control animals.

In general, the multidimensional nature of pain responses and the lack of sufficient specificity of clinical pain parameters, implies the need for composite measure schemes combining multiple behavioral, physiological, and biochemical parameters. Recently, we have developed a bioinformatic workflow for the selection of parameters to design composite measure schemes suitable for evidence-based severity assessment^26,27^. The bioinformatic workflow and the resulting CMS have been validated with different induced and genetic rat and mouse models of neurological and neuropsychiatric disorders^26,27^. The data confirmed that the CMS allows allocating individual animals to severity levels and comparative severity assessment across groups and models. Applying the bioinformatic workflow to data sets of this study provided evidence that Neuro score, distance moved during the light phase, body weight change, nest building, MGS and FCMs are among the top parameters best distinguishing between the treatment groups. These findings further confirm the informative value of the respective parameters.

The cluster analysis provided a basis for comparing the different drug-control and surgical groups with each other and with naive-control animals. In this context, it is emphasized that the severity levels cannot be directly translated to the classification in the European Directive 2010/63/EU mild, moderate, and severe, i.e. the highest severity level in this study does not reflect a severe classification, but just the maximum level observed in this study.

Surprisingly allocation to severity levels in the different treatment groups did not confirm the superiority of the multimodal approaches. Regardless of sex, the lowest number of animals allocated to the highest severity level was evident in the group with NSAID monotherapy. These data may indicate that the administration of an NSAID alone might be sufficient for pain management following neurosurgical procedures in mice. Cho and colleagues (2019) have already demonstrated that injected and oral carprofen and meloxicam can efficaciously reduce MG scores following craniotomy in mice^2^. Despite this report, we expected that a combination of different analgesics and local anesthetics targeting different components of the nociceptive system ranging from the peripheral nociceptors to central processing of nociceptive signals would have the potential to exert synergistic effects.

In this context, it needs to be taken into account that a relatively high dose of carprofen (25 mg/ kg/ day) was administered and that relatively high drug exposure rates were indicated by the analysis of carprofen plasma concentrations in the present study. In view of the high drug exposure rates, the histopathological assessment, which identified mild erosive lesions in the stomach of 24% of carprofen-treated mice, must be considered. The histopathological assessment indicates that gastrointestinal tolerability might be limited in individual animals exposed to high-dose carprofen. The decision for this carprofen dose was based on national recommendations^49^ and findings by our collaborators^20^, who demonstrated tolerability of the dose. Considering local reactions that we observed with exposure to subcutaneous carprofen injections and pharmacokinetic data provided by Marion Bankstahl’s group^20^, the decision was made in favor of oral self-administration via a drinking solution. In line with findings by Glasenapp as well as Ingrao and colleagues^20,50^, an increase in drinking volume was observed on the first day of carprofen exposure in female mice, which might help with fast loading and rapidly reaching therapeutic plasma concentrations. On the other hand, one needs to avoid cumulation to non-tolerated concentrations because of the initial attractiveness of the carprofen solution. Moreover, data from buprenorphine-treated female mice with a transient drop in water intake suggest the need to carefully control the impact of other drugs. Other authors have described a transient decrease of food and water intake after surgical interventions in mice^12,37,38^, which can also limit the oral uptake of analgesia administered via the drinking water. Thus, while oral dosing via a drinking solution offers the advantage of avoiding repetitive handling and restraint, the disadvantages include inter-individual variability in dosing and challenges in controlling or compensating for influencing factors. It is emphasized that related to these potential limitations and influencing factors, it is of utmost importance to carefully monitor mice to detect breakthrough pain and to administer injectable rescue analgesia in respective cases.

Infiltration of the surgical area with local anesthetics offers the unique opportunity to block the transduction of nociceptive signals and limit sensitization processes in the nociceptive system^6,37^. However, analysis of various pain parameters did not provide evidence that additional local anesthesia potentiated the efficacy of the therapeutic regimens. Considering the high dose of bupivacaine, it is unlikely that this is related to the chosen dosage. A direct postsurgical effect on nociceptive signaling is probably limited considering evidence suggesting a short duration of action of up to one hour in mice^7,51^.

Sustained-release buprenorphine formulation can offer advantages concerning tolerability and administration-associated distress^7,12,13,52^. The flattening of plasma concentration curves with attenuation of C_max_ concentrations combined with more consistent and long-lasting therapeutic concentrations can limit concentration-related adverse effects and the risk of breakthrough pain. While commercial sustained-release formulations are available in the United States (US), there is a lack of respective pharmaceuticals in Europe. More recently, Schreiner and colleagues (2020) reported the development of a poly-lactic-co-glycolic acid (PLGA) based microparticulate buprenorphine formulation. The authors confirmed a rapid onset of action and a duration of analgesic effects for at least two days in mice^16^. In a follow-up study, effective postsurgical analgesia of 72 h became evident following administration of the BUP-Depot in a mouse femoral fracture model^17^. Our assessment in drug-control mice further supported excellent tolerability without any moderate or severe adverse effects in groups exposed to BUP-Depot. Effects in the Neuro score were limited to the opioid-specific impacts such as tail elevation, changes in locomotion and reactivity as already described in the literature^12,53^. The efficacy testing did not reveal a potentiation of analgesic efficacy by additional buprenorphine administration with the drug regimens used in the present study.

Taken together the failure to demonstrate synergistic effects might be related to different influencing factors including the high carprofen exposure levels. Our data indicate that oral administration of carprofen at high doses may exert effects that cannot be further potentiated by combination of buprenorphine or local anesthetics. Despite the absence of moderate or severe adverse effects in all treatment groups, the trend for higher severity levels in animals with multimodal regimens might point to a detrimental effect of the overall drug load in the surgical groups, which should be taken into account during the planning of pain management approaches. As already stated, an interaction between postsurgical pain and distress and drug adverse effects might contribute to the level of the detrimental effects.

As previously mentioned, a surgical group without analgesic drug treatment was not included for ethical reasons. A respective group would have been of interest for assessment of the levels and duration of postsurgical pain and distress in untreated animals, and for interpretation of our findings. In this context, we would like to emphasize that our study design has built on an earlier study by Cho and colleagues^2^, in which the authors have already demonstrated that relevant craniotomy-associated pain measured by MG-scores lasts up to 48 h in control mice treated with vehicle only^2^. Based on these findings, which provide robust proof for significant postsurgical pain and distress levels, we did not consider it necessary to include a respective control group, which would be exposed to a situation with uncontrolled postsurgical pain.

Of course, an interlaboratory and cross-study comparison and general conclusions need to carefully consider influencing factors including mouse strain, habituation, age, single housing, and handling procedures, which can limit translation to different laboratory environments.

In conclusion, our findings confirmed the informative value of Neuro score, home cage locomotion, body weight change, nest building, MGS and FCMs for assessment of postoperative pain and distress. Applying a bioinformatic workflow resulted in the design of a composite measure scheme allowing the allocation of individual animals to different severity levels and comparison between treatment groups. Comparative assessment failed to confirm the superiority of multimodal regimens in comparison with high-dose NSAID monotherapy, thus, indicating that the latter may result in sufficient analgesia. In this context, it needs to be considered that the pain level might be affected by various influencing factors.

While all drug regimens were well tolerated in control mice, our data suggest that the total drug load should be carefully considered for perioperative management. The benefits and harms of all analgesics should therefore be thoughtfully weighed up according to the principle of “as little as possible, as much as required”. Future studies would be of interest to assess whether synergism can be observed if lower doses of carprofen are compared with an opioid and/or a local anesthetic.

## Material and methods

### Ethical statement

All animal experiments were conducted in accordance with the EU Directive 2010/63/EU and the German Animal Welfare Act and reported in line with the ARRIVE (Animal Research: Reporting of In Vivo Experiments) guidelines 2.0^54^. The Basel declaration including the 3R principles was taken into account for all investigations. All animal experiments were approved by the government of Upper Bavaria (Munich, Germany, license number ROB-55.2-2532.Vet_02-19-157).

### Animals

Female and male C57BL/6J wildtype mice (n = 149) were obtained from Charles River Laboratory (Sulzfeld, Germany) and were kept under specific pathogen-free (SPF) conditions according to FELASA recommendations^55^. Experimental animals were aged between 84 and 90 days and weighed between 21.04 g and 26.36 g (M, females and males, respectively) at the start of the experiment (day 0, Mon). Animals were housed under controlled environmental conditions (22 ± 2 °C and 55 ± 10% humidity) in a 12-h dark–light cycle (CEST: 6 am to 6 pm, CET: 5 am to 5 pm) with ad libitum access to standard diet of food pellets for mice and rats (Ssniff Spezialdiäten GmbH, Soest, Germany) and tap water (250 ml water bottle, Ehret GmbH, Emmendingen, Germany). After they arrived at the animal facility, the animals underwent an acclimatization period of seven days. During the acclimatization period and baseline measurements, male mice were single-housed in Makrolon type III cages (Ehret GmbH, Emmendingen, Germany), equipped with wood chip bedding (SAFE select, J. Rettenmaier & Söhne GmbH & CO. KG, Rosenberg, Germany), one cotton nestlet (Zoonlab GmbH, Castrop-Rauxel, Germany), and a square mouse house (Zoonlab GmbH, Castrop-Rauxel, Germany). Upon arrival, female mice were housed in groups of four animals per cage for four days (day -11 to -7, Thur-Mon). Makrolon type III cages were equipped with wood chip bedding, two cotton nestlets, and a square mouse house. To obtain individual baseline measurements, female mice were separated after the initial group-housing period (day -7, Mon) and single-housed under the same conditions as males. During acclimatization, mice were accustomed to tail handling and neck fixations by the main female experimenter on a daily basis (day -7 to day -3, Mon-Fri).

During the monitoring phase, i.e. after the intervention (day 0 to day 4, Mon-Fri), mice were housed individually in a home cage system, providing continuous video recording (PhenoTyper, Noldus, Wageningen, Netherlands). As described previously^40^, each PhenoTyper was supplemented with approximately 200 g wood chip bedding material, one cotton nestlet, and an infrared translucent mouse house (Noldus, Wageningen, Netherlands). A running wheel (PhenoWheel (diameter: 15 cm, width: 7 cm), Noldus, Wageningen, Netherlands) was mounted into the home cage system on day 1 and provided until day 4 (Tue-Fri). Standard diet for mice and rats and tap water or carprofen-treated tap water (drinking bottle, Noldus, Wageningen, Netherlands) were provided ad libitum. Mice were checked daily by applying a clinical score.

### Study design

Mice (n = 144) were randomly allocated (R version 4.1.1^56^, simple randomization) to one of the following three experimental groups: surgery (n = 64), drug-control (n = 64), and naive-control group (n = 16) (Fig. 1b). Both, the mice in the surgery and drug-control group, were anesthetized, while only the mice in the surgery group underwent an intracranial electrode implantation. In addition, depending on the respective analgesic regimen, the mice in the surgery group and in the drug-control group were assigned to one of the following subgroups: + N, + NL, + NO, + NLO (N = NSAID, L = local anesthetic, O = opioid; n = 16 per analgesic regimen). The sex ratio in each experimental group and subgroup was 1:1. Five mice had to be excluded during the study and were replaced by reserve animals (see supplementary information for details).

After the acclimatization period, baseline data (day -4 to -3, Thur-Fri) were collected for the behavioral, physiological, and biochemical parameters. On day 0, the surgery and drug-control group mice were anesthetized with isoflurane. Mice in the surgery group underwent an intracranial electrode implantation in the amygdala. In contrast, mice in the drug-control group were not subjected to a surgical procedure (see supplementary information for details). Behavioral, (patho)physiological, and biochemical parameters were assessed repeatedly after the mice recovered from anesthesia for four days daily. On day 4, mice were euthanized, and samples were collected for further analyses (Fig. 1a).

To reduce the risk of bias in- and exclusion criteria were determined prior to the study and measures to avoid batch effects were applied (see supplementary information). In contrast to the animal caregivers, the main female experimenter was not blinded to group allocation due to the administration of drugs. Except for the Neuro score, all parameters were analyzed retrospectively, whereby the experimenter was blinded to the allocation of subgroups and the time points. However, the video footage and image assessment unmasked the allocation to the surgery group, as mice with implants were clearly identified.

Because we observed local side effects at the injection sites of mice receiving subcutaneous carprofen injections in the neck fold, we conducted a pilot study to evaluate the tolerability and efficacy of oral carprofen administration as an alternative approach (see supplementary information). Local adverse effects occurred in mice receiving s.c. carprofen injections in the neck fold at a dosage of 20 mg/kg, using Rimadyl 50 mg/ml (Zoetis Deutschland GmbH, Berlin, Germany) diluted 1:10 with Aqua ad iniectabilia (B. Braun Melsungen AG, Melsungen, Germany). Mice showing local adverse effects had received one s.c. carprofen injection 60 to 90 min before surgery/anesthesia, followed by three postsurgical s.c. carprofen injections every 24 h.

### Analgesia

The NSAID carprofen (Rimadyl 50 mg/ml, Zoetis Deutschland GmbH) was administered at a dose of 25 mg/kg via drinking water. The daily baseline water intake per mouse was determined gravimetrically (bench scale, FCB 3K0.1, Kern & Sohn GmbH, Balingen, Germany) during the acclimatization period (day -7 to day -3, Mon-Fri), including a correction factor for dripping bottles in the calculation of bottle weight differences. Based on the mean baseline water intake per day and the mean body weight at the baseline time point, the concentration of carprofen in the stock solution was calculated for each batch individually (males M = 0.128 mg/ml (SD = 0.021); females M = 0.123 mg/ml (SD = 0.011)). Mice were supplied with carprofen-treated water 20-24 h before surgery (day -1, Sun) and had ad libitum access until four days (day 0 to day 4, Mon-Fri) after surgery. Carprofen-treated water intake was measured on a daily basis, applying correction factors for bottle drips for the intake calculation.

The sustained-release buprenorphine formulation (BUP-Depot) was used as an opioid. The lyophilisate (1.25 mg buprenorphine hydrochloride/vial) was reconstituted with 4 ml of Aqua ad iniectabilia. BUP-Depot was injected subcutaneously at a dosage of 1.2 mg/kg in the lower abdominal region 60-90 min before surgery.

Bupivacaine 0.25% with epinephrine 0.00025% (bupivacaine 0.5% with epinephrine 0.0005% (Jenapharm, Mibe GmbH, Brehna, Germany) diluted 1:1 with saline (Isotonische Natriumchlorid-Lösung ad us. vet., B. Braun Melsungen AG, Melsungen, Germany)) was administered subcutaneously to the + NL and + NLO subgroups at a total dosage of 8 mg/kg. In the + NL and + NLO surgery mice, this dosage was divided into a subcutaneous administration at the incision line during anesthesia and a direct administration of one drop on the exposed skull during surgery. Regardless of whether the surgery mice received a bupivacaine injection, the surgical procedure began after a 15-min waiting period in all subgroups.

### MGS

For MGS video recordings, mice were put in plexiglas cubicles (L9 x W5 x H5 cm) and placed in a recording rack exposed to 400–410 lx. Videos were recorded for 10 min with a reflex camera (EOS 800D Digital Camera + EF-S 18-55 mm f/4–5.6 IS STM objective, Canon Inc, Tokyo, Japan). Videos were recorded on day -4 (Thurs) for baseline, on day 0 (Mon) at 2 h, 4 h, 6 h, 8 h post-recovery as well as on day 1 to day 4 (Tues–Fri, 1.5-5 h after light change). A frame-grabbing tool^57,58^ extracted about 600 images from the recorded video file. Images were then manually preselected using the following image quality criteria: 1) mouse in profile or front view, 2) at least one eye visible (exclusion of eye closure due to blinking or sleeping), 3) nose and cheek area visible, 4) at least one ear visible, 5) mouse is static and calm (exclusion of sniffing or grooming), and 6) good image quality^58^. Afterward ten pictures of the preselected ones were chosen for every minute, picking the image closest to xx:30 min. Ten pictures per mouse and time point were scored blinded and randomized by a single, trained scorer, using the MGS score tool^57,58^ and applying the previously described MG score^24^. Thereafter, a mean MG score was calculated for each animal and time point. If there were difficulties in evaluating individual action units (e.g. whiskers), this was taken into account when calculating the mean MGS. In addition, the sum MG score with and without the action unit “whisker change” was calculated by adding the mean action unit scores of 10 images per mouse and time point.

### Home cage-based behavioral assessment

#### Activity

General activity (distance moved, velocity) was assessed continuously from 2 h post-recovery on day 0 (Mon) onwards. Video recordings from PhenoTyper home cages and the integrated tracking software EthoVision XT 15 (EthoVision XT, RRID: SCR_000441) provided the duration mice spent in the pre-defined zone around the mouse house (house zone). Results are presented for 12 h time slots, representing the light and dark phases in the animal facility. In addition, the first 20 h post-recovery time slot is shown separately.

#### Nest building

To assess baseline nest building behavior, nest pictures (LUMIX DMC-LF1, Panasonic Corporation, Kadoma, Japan) in three different angles (top view and two side views at an angle of 90 and 45 degrees) were taken 2 h, 4 h, and 6 h after cage change on day -4 (Thurs) and 1–2 h after light change on day   -3 (Fri). To assess postsurgical nest building behavior, nest pictures were taken 4 h, 6 h, and 8 h post-recovery on day 0 and 1–2 h after light change on day 1 to day 4 (Tues–Fri). The scorer of the nest pictures was blinded to treatment allocation. Details on the scoring are provided in the supplements.

#### Burrowing

To habituate mice to the burrowing set-up, a pellet-filled burrowing bottle was put into the home cage on days -9 to -7 (Sat–Mon). Baseline burrowing behavior was assessed on days -4 to -3 (Thurs–Fri). Directly after cage change, a burrowing bottle (length: 20 cm, diameter of the bottleneck: 3.5 cm; Zoonlab GmbH, Castrop-Rauxel, Germany), filled with 200 ± 1 g standard food pellets for mice and rats, was placed in the cage. The weight of the burrowed pellets was measured 2 h, 4 h, 6 h, and 20 h after cage change. Additional lateral video recording (Axis M1065-L Network Camera, Axis Communications AB, Lund, Sweden) was used for the analysis of the latency to burrow (displaying burrowing behavior for > 10 s^18^ or > 20 s with short breaks of maximum 5 s). If no burrowing behavior was observed on video recordings at baseline time point, the mouse was excluded from further analysis of burrowing post-recovery. The same experimental set-up was used post-recovery on day 0 in the PhenoTyper, and burrowed weight was measured 4 h, 6 h, 8 h, and 20 h post-recovery. If no burrowing behavior was observed on video recordings post-recovery, the value for latency to burrow was set to 72,000 s, resembling the total duration of observation.

### Body weight

Body weight was measured (bench scale, FCB 3K0.1) on arrival, day -4 (Thur), day -3 (Fri), presurgical (approximately 1 h after light change), directly post-recovery, 6 h post-recovery (Mon), and on day 1 to 4 (Tues–Fri). To evaluate the development of the mice´s body weight, the percentage change in body weight was calculated and normalized to the individual mean baseline weight.

### FCMs

Fecal samples were collected from the MGS box, the weighing cage, and 2 h after cage change on day -7 (Mon, females only) and day -4 (Thurs) for baseline values and accordingly on day 1 (Tues) and day 4 (Fri) for postsurgical values (see supplementary information).

### Neuro score

The Neuro score (see description of the assessment of the Neuro score in the supplementary information and information on the scoring system in Table S18), a modified Irwin Score was applied on day -4 (Thurs) for baseline values and on the post-recovery time point 4 h on day 0 (Mon) as well as on day 1 to day 4 (Tues–Fri).

### Euthanasia and sampling

On day 4 (Fri), mice were euthanized with 600 mg/kg Pentobarbital (Narkodorm, 182.3 mg/ml Pentobarbital, CP-Pharma Handelsges.mbH, Burgdorf, Germany), injected intraperitoneally, receiving 100 mg/kg Metamizole (Vetalgin, 500 mg/ml Metamizole/dipyrone, MSD Animal Health, Munich, Germany), diluted 1:10 in 0.2% saccharine solution *per os* 30 min beforehand. Directly after euthanasia, skin samples of BUP-injection site were harvested, cardiac exsanguination and transcardial perfusion fixation with 4% PFA followed. After the perfusion the stomach and duodenum were extracted and underwent gross examination. Additionally, the brain was harvested. Blood samples were transferred in 1.3 ml EDTA tubes (Sarstedt, Nümbrecht, Germany) and centrifuged at 4° Celsius, 2500 *g* for 10 min (Centrifuge 5418 R, Eppendorf SE, Hamburg, Germany) to obtain plasma samples. The concentration of carprofen in plasma samples was analyzed with liquid chromatography-mass spectrometry^20^. Stomach, duodenum, and haired skin samples were investigated histomorphologically (see supplementary information for details).

### Statistical analyses

The sample size calculation was conducted to determine the number of animals with an a priori power analysis for the main readout parameter MGS (see supplementary information for details). Differences between the naive-control group and drug-control group and between the naive-control group and surgery group were analyzed using two-way repeated-measures (RM) analysis of variance (ANOVA) or one-way ANOVA. Significant differences were further investigated using a Bonferroni multiple comparison post hoc test. Since RM ANOVA cannot handle single missing values, data were analyzed by fitting a mixed effects model (compound symmetry covariance matrix, restrict maximum likelihood fitting, fixed factors: time, analgesic regimen; random factors: subject). Following the pre-study statistical analysis plan, the MGS was analyzed with Two-way RM ANOVA. Where indicated, data were analyzed with non-parametric statistics using the Kruskal–Wallis test and Dunn´s multiple comparison test. In this study, we aimed to investigate the effects of time and analgesic regimens in surgery and drug-control mice compared to naive controls. Therefore, the effects of sex and respective interactions of sex were not investigated, and analyses were performed for each sex separately. A *p*-value < 0.05 was considered as statistically significant. Significant differences in post hoc tests for naive-control vs. drug-control or surgery are reported in the text and detailed in the supplementary information. They are illustrated in graphs, while significant findings between the subgroups are only reported in the supplementary information. Data are presented as mean ± SD or median with interquartile range (IQR). Statistical analyses and graphical illustrations were conducted using GraphPad Prism 10.0.2 (GraphPad Software, Boston, MA, USA) and R version 4.1.1^56^. Spearman correlation was calculated and visualized using the R package *corrplot*^59^. All other graphical illustrations, including plots for principal component analyses (PCA), were created with *ggplot2*^60^. As described previously^26,27^, datasets were subjected to a bioinformatic workflow to design a composite measure scheme using the R package available at https://github.com/mytalbot/cms.

### Supplementary Information

Supplementary Information.

## Data Availability

All raw datasets of the study are available in the Figshare repository (DOI:  10.6084/m9.figshare.26030569).
