# Supplementary material for: Refining pain management in mice by comparing multimodal analgesia and NSAID monotherapy for neurosurgical procedures
Source: Sci Rep. 2024 Aug 12;14:18691. doi: 10.1038/s41598-024-69075-2 (PMC11319454; doi:10.1038/s41598-024-69075-2)
Supplement: Supplementary file 1 — Supplementary Information. [file 41598_2024_69075_MOESM1_ESM.docx]

**Refining pain management in mice by comparing multimodal analgesia and NSAID monotherapy for neurosurgical procedures**

Anna Munk^a^, Vanessa Philippi^a^, Verena Buchecker^a^, Marion Bankstahl^b^, Aylina Glasenapp^b^, Andreas Blutke^c^, Effrosyni Michelakaki^c^, Steven Roger Talbot^b^, Jörg Huwyler^d^, Paulin Jirkof^e^, Marcin Kopaczka^f^, Dorit Merhof^g^, Rupert Palme^h^ and Heidrun Potschka^a*^

^a^Institute of Pharmacology, Toxicology, and Pharmacy, Ludwig-Maximilians-Universität München, Munich, Germany
^b^Institute for Laboratory Animal Science, Hannover Medical School, Hanover, Germany
^c^Institute of Veterinary Pathology, Ludwig-Maximilians-Universität München, Munich, Germany

^d^Department of Pharmaceutical Sciences, University of Basel, Basel, Switzerland

^e^Office for Animal Welfare and 3R, University of Zurich, Zurich, Switzerland

^f^Department of Electrical Engineering, RWTH Aachen University, Aachen, Germany

^g^Department of Informatics and Data Science, University of Regensburg, Regensburg, Germany

^h^Department of Biological Sciences and Pathobiology, Experimental Endocrinology, University of Veterinary Medicine, Vienna, Austria

**Correspondence*:

Heidrun Potschka: potschka@pharmtox.vetmed.uni-muenchen.de

***Supporting Information***

**Page:**

1. **Supplementary Results** 3 - 5
2. **Supplementary Methods** 6 - 13
3. **Supplementary Figures** 14 - 25
4. **Supplementary Tables** 26 – 64
5. **Supplementary Results**

***Pilot study***

Prior to the start of the main study, we conducted a pilot study to assess the tolerability and efficacy of oral carprofen administration for postoperative pain management after intracranial surgery. Mice were assigned to one of the following two groups: (a) mice receiving carprofen 20-24 hours before surgery and four days after surgery via drinking water, and (b) mice receiving one preoperative injection of carprofen, followed by *ad libitum* access to carprofen-enriched water for four days after surgery. Clinical scores, body weight data, and Neuro scores did not indicate any tolerability issues with orally administered carprofen. However, histopathological evaluation revealed two mice in each of the analgesic groups showing minor ulcerative lesions in the non-glandular part of the stomach. At the injection site of one mouse in group b), which received a preoperative injection of carprofen, macroscopic skin lesions were identified. Burrowing and nest building performance as well as body weight development indicated better postsurgical pain management in group a) (Table S1). In summary, the data obtained in our pilot study suggest that oral carprofen is safe to use in C57BL/6J mice for craniotomy-associated pain management. Because one mouse in group b) developed lesions at the injection site, we decided not to administer an initial dose of carprofen subcutaneously directly before surgery.

***Mouse Grimace Scale (MGS)***

To facilitate a direct comparison between the MGS results of this study and other studies, additional illustrations of the sum MGS are provided (see Fig. S1 and S2). For each mouse and scoring time point, 10 pictures were evaluated for five action units, resulting in 50 score values. The mean scores for each action unit were then calculated, resulting in five mean action unit scores. Instead of calculating the mean of the five mean action unit scores, the five mean action unit scores were added up to form the sum MGS. Furthermore, the percentages of non-assessable action units within all assessable MG-pictures are displayed in supplementary Table S2. Since in 5 % of the scoring cases the action unit ‘whisker change’ could not be evaluated in 10 images, the sum MGS was illustrated with and without this action unit. The sum MG scores of male drug-control mice were transiently increased 2 h after anesthesia in all analgesic subgroups, 4 h and 6 h after anesthesia in the +NLO subgroup only and on day 1 in the +NL subgroup. In female drug-control mice a sum MG score increase was identified in +NL, +NO, +NLO sugroups 2 h and in +N and +NLO subgroups 4 h after anesthesia. Regardless of analgesic regimens, sum MG scores of male surgery mice were significantly impacted on the surgery day and on day 1. On day 2 male sugery mice of the subgroups +NL, +NO and +NLO still showed increased sum MG scores. Except for mice of the +NL subgroup 2 h after surgery, the MG scores of female surgery mice were significantly increased on the surgery day. This increase was still detectable on day 1 in +N, +NO and +NLO as well as on day 4 in +NL female surgery mice (Fig. S1, Table S3). The results of the analyses of the sum MGS without the action unit ‘whisker change’ revealed only minor differences: While the sum MG scores of female +N drug-control mice 4 h after anesthesia and female +N surgery mice on day 1 was not affected anymore, the sum MG scores of male +NLO surgery mice on day 4 and female +NL surgery mice 2 h after surgery were significantly increased (Fig. S2, Table S3). When interpreting these results of the sum MGS, the exlusion of values due to the lack of assessiblity of the action unit ‘whisker change’ in all 10 pictures per mouse per time point must be taken into account (cases: n = 14 male and n = 61 female).

***Home cage-based behavioral assessment***

**Velocity**

The reduced activity was also reflected by a reduced mean velocity in surgery mice of the subgroups +N, +NL, and +NLO within the first 20 h after surgery (Fig. S3b, Table S4).

**Zone in-house**

Because some mice used the burrowing bottle as shelter or built their nest outside of the mouse house, the time mice spent in the zone ‘house’ had to be excluded from the analysis.

**Voluntary Wheel Running (VWR)**

The analysis of VWR activity, which was measured from the dark phase of day 1 onwards, failed to detect any impact of the analgesic regimen, but did reveal a significant time-dependence in the VWR activity in drug-control and surgery mice of both sexes (Fig. S3c, Table S4).

**Burrowing**

During the twenty-hour burrowing test, the overall performance in removing pellets from the burrowing bottle was affected by time in mice of both sexes. Although the two-way RM ANOVA indicated an impact of the analgesic regimen on the amount of removed pellets in surgery mice of both sexes, significant differences between subgroups were not confirmed by the subsequent *post hoc* test (Fig. S4b, Table S6). The analysis of the baseline burrowing performance (delta (▲)-burrowing = baseline burrowing – experimental burrowing) indicated an effect of the analgesic regimen on the amount of pellets burrowed by female drug control-mice (two-way RM ANOVA), which, however, could not be confirmed by the subsequent *post hoc* test (Fig. S4c, Table S6).

***Body weight***

The two-way RM ANOVA or mixed-effects analyses of the absolute body weight data revealed a main effect of time only (Fig. S5a, Table S7).

***Carprofen plasma concentration***

Surprisingly, very low carprofen concentrations of 0.33 µg/ml (SD = 0.44) or 0.13 µg/ml (SD = 0.05) could be measured in naive-control male and female mice that were only exposed to tap water. These findings suggest a remaining carprofen contamination of cleaned water bottles in our animal facility. We were able to confirm this assumption by testing water samples of cleaned drinking bottles, which had been exposed to carprofen-treated water before.

1. **Supplementary Methods**

***Pilot study***

Six C57BL/6J mice (Charles River Laboratory, Sulzfeld, Germany; 70-76 days old at arrival) of each sex received an intracranial electrode implantation and were equally assigned to one of the following two groups:

a) Mice (n = 6) receiving carprofen 20-24 hours before surgery and four days after surgery via drinking water (25 mg/kg carprofen)

1. Mice (n = 6) receiving one preoperative injection of carprofen (20 mg/kg, s. c. injection in the neck fold, 60 – 90 min prior to surgery), followed by *ad libitum* access to carprofen-enriched water (25 mg/kg carprofen) for four days after surgery

In addition to the analgesic treatment above, both groups received local anesthetics and opioids as described for the main study. The experimental procedure and the assessment of postoperative pain were consistent with the main study.

***Study design***

Experiments were conducted in 20 batches with n = 8 mice each. Male mice were assessed prior to female mice (experimental periods: Oct. 2022 to Jan. 2023 (CET) for males and April 2023 to July 2023 (CEST) for females). To avoid batch effects, various experimental groups and subgroups were included per batch. In each batch (except for 2 batches with reserve animals), three to four analgesic regimens, and the naive-control group were represented. Moreover, to reduce confounding factors, the mice in the surgery and drug-control group that received the same analgesic regimen were paired and underwent all experimental procedures in parallel. The sequence of subgroups undergoing anesthesia or surgery and subsequent experiments was randomized (R version 4.1.1^1^, simple randomization) within each batch. Placing the subgroup pairs in the PhenoTyper rack followed a rotating system. Prior to the study, in- and exclusion criteria were determined: First, only healthy, C57BL/6J wildtype SPF-mice obtained from Charles River and only mice with intact whiskers were included in the study. Second, the following criteria were defined as exclusion criteria: 1) severe wound infection, intrasurgical bleeding or loss of electrode implant, 2) reaching of humane endpoint (definition see below), 3) no burrowing behavior in baseline (latency to burrow), 4) mal- or dysfunction of equipment. During the study, five mice had to be excluded due to the following reasons: humane endpoint reached (n = 1), severe bleeding in surgery (n = 1), and barbering during acclimatization period (n = 3). The five dropouts were replaced by reserve animals and underwent the experimental procedure in two reserve batches that, as an exception, did not reach animal numbers of 8 per batch. One male mouse of the surgery +N subgroup reached the humane endpoint at day 3 due to evidence for breakthrough pain. Collected data up to day 3 of this mouse were included in further analyses. If the group size differed from n = 8 due to the exclusion of animals or missing experimental values, this is explicitly mentioned in the respective figure legends.

***Surgery and anesthesia***

The surgical procedure took place under isoflurane anesthesia (introduction: 4%, maintenance 1.8 – 2%) (Isofluran 1 ml/ml, CP-Pharma Handelsges.mbH, Burgdorf, Germany). Mice were placed on a heating mat (PVC Heizmatte IP67, Dragon Terraristik Bedarf, Duisburg, Germany). Body temperature (Acorn Temp JKT Hermocouple Thermometer, Oakton) and the toe pinch reflex were monitored regularly. Eyes were lubricated with eye ointment (Bepanthen® Augen- und Nasensalbe; Bayer Vital GmbH, Leverkusen, Germany). The skull of the mouse was fixed in a stereotactic device (TSE Systems GmbH, Bad Homburg, Germany). After shaving and disinfection (octenisept®; Schülke & Mayr GmbH, from Covetrus DE GmbH, Norderstedt, Germany), the skin of the skull was incised and the surgical site exposed. A small hole, using a dental drill (spiral drill, diameter 1.2 mm, Fischer Goldschmiedebedarf, Pforzheim, Germany) was drilled in the skull, to implant a depth-electrode in the right amygdala (coordinates with regard to bregma males: lat 3.3, ap -1.4, dv 5.0; coordinates females: lat 3.3, ap -1.4, dv 4.9). In addition, three further holes were drilled (spiral drill, diameter 1.0 mm, Fischer Goldschmiedebedarf, Pforzheim, Germany) for the fixation screws (BN 650, M 1.2x2, Schrauben Preisinger GmbH, Munich, Germany). The electrode was stabilized with dental cement (Paladur®; Heraeus, Hanau, Germany) supported by intraosseal fixation screws. Skin margins were adapted with sutures (Surgicryl Monofilament, reabsorbable, USP 5/0 EP 1Smi AG, St. Vith, Belgium). At the end of surgery, balanced electrolyte solution (Ringer-Lactat Infusionslösung, B. Braun Melsungen AG, Melsungen, Germany) was injected subcutaneously to compensate loss of fluid. After waking up from anesthesia, the cages of the mice were placed on heating mats for full recovery. The time point when mice showed reflexes and regained consciousness was defined as recovery time point. Mice in the drug-control group underwent isoflurane anesthesia in parallel with the mice in the surgery group of the same treatment subgroup.

***Home cage-based behavioral assessment***

**Voluntary Wheel Running**

The PhenoWheel was installed on day 1 (Tues) and was continuously accessible to mice until day 4 (Fri). Total distance run was analyzed with the software EthoVision XT 15, based on the wheel turns per minute.

**Nest building**

The nest complexity was assessed by applying following score modified from Jirkof et al.^2^.

- Score 1: Nestlets are almost not manipulated (> 90% are intact)
- Score 2: Nestlets are slightly manipulated (50-90% are intact)
- Score 3: Nestlets are largely manipulated (50-90% are torn)
  - < 50% are intact
  - < 90% are in one quarter of the cage
  - Shreds are placed all over of the cage and not in the shape of a nest
- Score 4: Flat nest (> 90% are torn)
  - Shreds are placed in one quarter of the cage
  - < 50% of the walls are higher than one third of the height of the mouse house
- Score 5: Nearly perfect nest (> 90% are torn)
  - 50% of the walls but < 90% are higher than one third of the height of the mouse house
- Score 6: Perfect nest (> 90% are torn)
  - 90% of the walls are higher than one third of the height of the mouse house

***Fecal corticosterone metabolites (FCMs)***

Samples were stored at -20 °C, homogenized repeatedly, and dried by 60 °C in the hot cabinet overnight twice. 0.05 g of each feces sample was extracted in 1 ml of 80 % methanol. The supernatant was pipetted, and the extracted samples were stored at -80 °C. A 5α-pregnane-3β,11β,21-triol-20-one enzyme immunoassay was conducted to quantify FCM concentrations^3,4^.

***Neuro score***

The Neuro score has been designed based on the Irwin Test, which is a well-known procedure for assessment of the behavioral and physiologic state of mice and the response of the central, peripheral and autonomous nervous system to drugs^5^. The modification comprised the exclusion of parameters that were not considered valuable in the context of severity assessment, as well as parameters whose assessment could be associated with distress and pain such as ‘toe pinch’ or ‘tail pinch’. Moreover, we have added specific parameters for postsurgical assessment such as ‘wound healing’. For all parameters a score ‘0’ indicates evidence for a normal condition. All other scores indicate evidence for a deviation from normal. Please note that for selected parameters the directionality was reflected by adding minus or plus signs (e.g. ‘locomotor activity’: ‘-1’ = reduced activity, ‘+1’ = increased activity). However, to calculate the sum Neuro score the single scores were always summed up without taking these signs into account.

As a modified scoring system, the Neuro score already provided valuable information for severity assessment in several studies assessing the severity of rodent models of neurophysiological and psychiatric disorders^6-16^. In this study, the Neuro score was assessed by two well-trained female experimenters during the course of the daily behavioral testing procedure to minimize handling-related distress. To further minimize the interference of parameters and a potential impact of handling-associated distress on the readouts, the order of assessment was based on the invasiveness of the individual parameters. First, parameters were collected for which the animals were visually inspected in their home cages for a few minutes, causing a minimum of distress to the animals. Next, the cage lid was removed and the parameters ‘curiosity’, ‘startle’ and ‘touch reaction’ were evaluated. To assess the parameter ‘curiosity’, the mouse was presented with a pencil approximately 3 cm in front of its nose and its reaction to the object was evaluated. To assess the paremeter ‘startle’, the experimenter tapped the cage wall opposite the mouse once with a pencil and the mouse´s reaction to the sound was observed. For the parameter ‘touch reaction’ the experimenter slightly stroked over the mouse´s back three times in succession and observed its reaction to the touch. Following assessment in the home cage (with cage lid removed), the mouse was taken out of its home cage to monitor the body weight and collect video footage for the analysis of the MGS. The parameters whose collection required direct interaction with the animal, associated with more intense handling, were collected at the end of the daily behavioral testing procedure. Mice were hold in the neck to assess the parameters ‘vocalization while handling’, ‘irritability’, ‘abdominal tone’, ‘body tone’, ‘urination’, and ‘defecation’. If the mouse displayed aggressive behavior during fixation (e.g. attempted biting), this was scored as ‘irritability’. The experimenter lightly touched the abdomen to obtain information about the ‘abdominal tone’. A detailed description is provided in Table S18.

Please note that the parameters ‘urination’, ‘defecation’, and ‘vocalisation while handling’ were not considered for the sum Neuro score and the processing in the bioinformatic workflow to design a CMS as they are not considered relevant in the context of postsurgical severity assessment (Table S12).

***Quantification of carprofen concentration in plasma***

Carporfen concentrations were quantified using liquid chromatrography-mass spectrometry as previously described in detail by Glasenapp and colleagues (2023)^17^.

***Histopathology of gastrointestinal tract and injection sites samples***

The examination was performed on formalin-fixed, paraffin-embedded tissue sections, using standard Haematoxylin & Eosin (HE) and Giemsa stains for all samples and additionally PAS-reaction for skin samples. All cases were independently evaluated by two pathologists. Only skin samples of +NO and +NLO analgesic supgroups (n = 64) were analyzed, as local adverse effects at the injection site have been described for commercially available sustained-release buprenorphine formulations^18,19^. Since minor alterations were reported by the pathologists during the couse of the study, we then decided to additionally examine exemplary skin samples of naive-control mice (n = 5) for a direct comparison.

***Humane endpoint***

Mice were monitored daily with a focus on general condition, habitus, body weight, behavior, motor function, and postsurgical wound healing. The physiological state was scored with a value of 0. Alterations from the physiological state were scored on a scale of 1 to 3 (mildly, moderately, or severly altered state, respectively). A total score was calculated based on the sum of the deviations of individual parameters. If any single parameter scored ≥3 and/or the sum of the scores scored ≥5, the humane endpoint was reached and the animal was euthanized.

***Statistical analyses***

The sample size calculation to determine the number of experimental units, was conducted with an a priori power analysis using the program G*Power 3.1.9.2. (University of Düsseldorf). One experimental unit consisted of one single animal. The MGS (effect size f = 0.41, biological relevant difference = 0.25, SD = 0.3) was selected as main readout parameter for this analysis. The F tests-ANOVA: repeated measures and F test-ANOVA: fixed effects, omnibus, one-way were chosen as statistical tests. The alpha error was set to 0.05 and the power to 80 %. The power analysis resulted in following group sizes for 18 groups: F test-ANOVA repeated measures: between factors n = 5, within-between factors n = 2, within factors n = 2 and F test-ANOVA fixed effects, omnibus, one-way: n = 8. A total sample size of n = 144 with n = 8 animals per group is required to declare results between groups statistically significant with a power of 0.80 and a significance level of 0.05. In addition, effect sizes (Cohen´s d, using pooled standard deviations) were calculated for all parameters and time points, respectively, by comparing readouts from male and female naive-control mice to sex-matched surgery and drug-control mice, based on the effect of the analgesic regimen. Effect size calculation was performed using the R package ‘*effsize*’^20^. Effect sizes (Cohen’s d) are provided in Table S17.

***References***

1 R: A Language and Environment for Statistical Computing (R Foundation for Statistical Computing, Vienna, Austria, 2022).

2 Jirkof, P. *et al.* Assessment of postsurgical distress and pain in laboratory mice by nest complexity scoring. *Lab Anim* **47**, 153-161, doi:10.1177/0023677213475603 (2013).

3 Touma, C., Palme, R. & Sachser, N. Analyzing corticosterone metabolites in fecal samples of mice: a noninvasive technique to monitor stress hormones. *Horm Behav* **45**, 10-22, doi:10.1016/j.yhbeh.2003.07.002 (2004).

4 Touma, C., Sachser, N., Möstl, E. & Palme, R. Effects of sex and time of day on metabolism and excretion of corticosterone in urine and feces of mice. *Gen Comp Endocrinol* **130**, 267-278, doi:10.1016/s0016-6480(02)00620-2 (2003).

5 Irwin, S. Comprehensive observational assessment: Ia. A systematic, quantitative procedure for assessing the behavioral and physiologic state of the mouse. *Psychopharmacologia* **13**, 222-257, doi:10.1007/bf00401402 (1968).

6 Möller, C. *et al.* Impact of repeated kindled seizures on heart rate rhythms, heart rate variability, and locomotor activity in rats. *Epilepsy Behav* **92**, 36-44, doi:10.1016/j.yebeh.2018.11.034 (2019).

7 Koska, I. *et al.* Toward evidence-based severity assessment in rat models with repeated seizures: II. Chemical post-status epilepticus model. *Epilepsia* **60**, 2114-2127, doi:10.1111/epi.16330 (2019).

8 Boldt, L. *et al.* Toward evidence-based severity assessment in mouse models with repeated seizures: I. Electrical kindling. *Epilepsy Behav* **115**, 107689, doi:10.1016/j.yebeh.2020.107689 (2021).

9 Buchecker, V. *et al.* Toward evidence-based severity assessment in mouse models with repeated seizures: (II.) Impact of surgery and intrahippocampal kainate. *Eur Surg Res* **64**, 30-48, doi:10.1159/000522156 (2022).

10 Reiber, M. *et al.* Development of behavioral patterns in young C57BL/6J mice: a home cage-based study. *Sci Rep* **12**, 2550, doi:10.1038/s41598-022-06395-1 (2022).

11 Reiber, M., Miljanovic, N., Schönhoff, K., Palme, R. & Potschka, H. Behavioral phenotyping of young Scn1a haploinsufficient mice. *Epilepsy Behav* **136**, 108903, doi:10.1016/j.yebeh.2022.108903 (2022).

12 Seiffert, I. *et al.* Toward evidence-based severity assessment in rat models with repeated seizures: III. Electrical post-status epilepticus model. *Epilepsia* **60**, 1539-1551, doi:10.1111/epi.16095 (2019).

13 Di Liberto, V. *et al.* Imaging correlates of behavioral impairments: An experimental PET study in the rat pilocarpine epilepsy model. *Neurobiol Dis* **118**, 9-21, doi:10.1016/j.nbd.2018.06.010 (2018).

14 Aulehner, K. *et al.* The impact of tethered recording techniques on activity and sleep patterns in rats. *Sci Rep* **12**, 3179, doi:10.1038/s41598-022-06307-3 (2022).

15 van Dijk, R. M. *et al.* Imaging biomarkers of behavioral impairments: A pilot micro-positron emission tomographic study in a rat electrical post-status epilepticus model. *Epilepsia* **59**, 2194-2205, doi:10.1111/epi.14586 (2018).

16 Reiber, M. *et al.* Phenotyping Young GluA1 Deficient Mice - A Behavioral Characterization in a Genetic Loss-of-Function Model. *Front Behav Neurosci* **16**, 877094, doi:10.3389/fnbeh.2022.877094 (2022).

17 Glasenapp, A., Bankstahl, J. P., Bähre, H., Glage, S. & Bankstahl, M. Subcutaneous and orally self-administered high-dose carprofen in male and female mice: pharmacokinetics, tolerability and impact on cage-side pain indicators. *bioRxiv*, 2023.2006.2003.543582, doi:10.1101/2023.06.03.543582 (2023).

18 Clark, T. S., Clark, D. D. & Hoyt, R. F., Jr. Pharmacokinetic comparison of sustained-release and standard buprenorphine in mice. *J Am Assoc Lab Anim Sci* **53**, 387-391 (2014).

19 Carbone, E. T., Lindstrom, K. E., Diep, S. & Carbone, L. Duration of action of sustained-release buprenorphine in 2 strains of mice. *J Am Assoc Lab Anim Sci* **51**, 815-819 (2012).

20 effsize: Efficient Effect size computation v. 0.8.1 (2020).

1. **Supplementary Figures**

**Supplementary figure S1: Sum MGS**

The sum MGS (sum of mean action units per mouse per time point) including the action unit ‘whisker change’ is illustrated for the drug-control and surgery groups compared to naive-control groups in both sexes. If the action unit ‘whisker change’ was not assessable in all 10 pictures per mice per time point, sum MG scores were excluded from the illustrations and analyses. (group sizes differing from n = 8: males BL drug-control +NL n = 7; 2h surgery +NO, +NLO, drug-control +N n= 7, drug-control +NL, +NO n = 6; 4h surgery +NL, drug-control +NO n = 7; 6h surgery +NO n = 6, drug-control +NL n = 7; 8h surgery +NL n = 7; day 1 surgery +NO n = 7; day 4 surgery +N, +NO n = 7. Females BL surgery and drug-control +NO n = 6, drug-control +NL n = 7; 2h surgery +N, +NO, drug-control +N, +NL, +NLO, naive-control n = 6, sugery +NL n = 3, drug-control +NO n = 5; 4h surgery +N, +NO n = 6, surgery +NO n = 5, drug-control +N, +NL, +NLO n = 7, drug-control +NO n = 4; 6h surgery +NL, +NLO n = 7, drug-control +N, +NO n = 5; 8h surgery +N, drug-control +NLO n = 7, drug-control +NO n = 5; day 1 drug-control +NO n = 6, naive-control n = 7; day 3 surgery +N, drug-control +NO n = 7; day 4 drug-control +NLO n = 7). (* = p < 0.05; mean ± SD; Mixed effects model with Bonferroni *post hoc* test; BL = baseline).


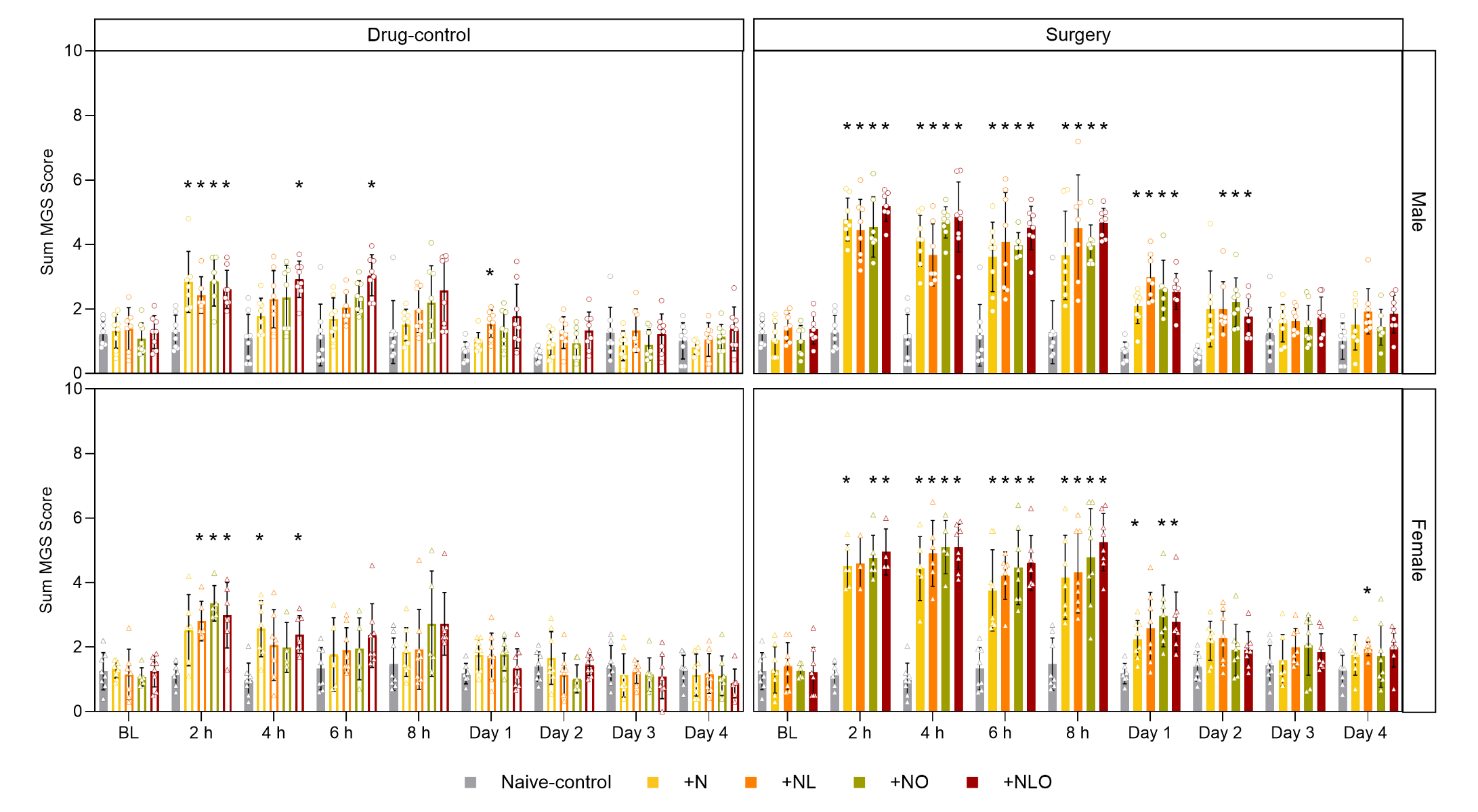


**Supplementary figure S2: Sum MGS without ‘whisker change’**

The sum MGS (sum of mean action units per mouse per time point) without the action unit ‘whisker change’ is illustrated for the drug-control and surgery groups compared to naive-control groups in both sexes (group sizes differing from n = 8: males 4 h surgery + NL n = 7, 6h drug-control +NL n = 7, day 4 surgery and drug-control +N n = 7). (* = p < 0.05; mean ± SD; Two-way RM ANOVA/ Mixed effects model with Bonferroni *post hoc* test; BL = baseline).


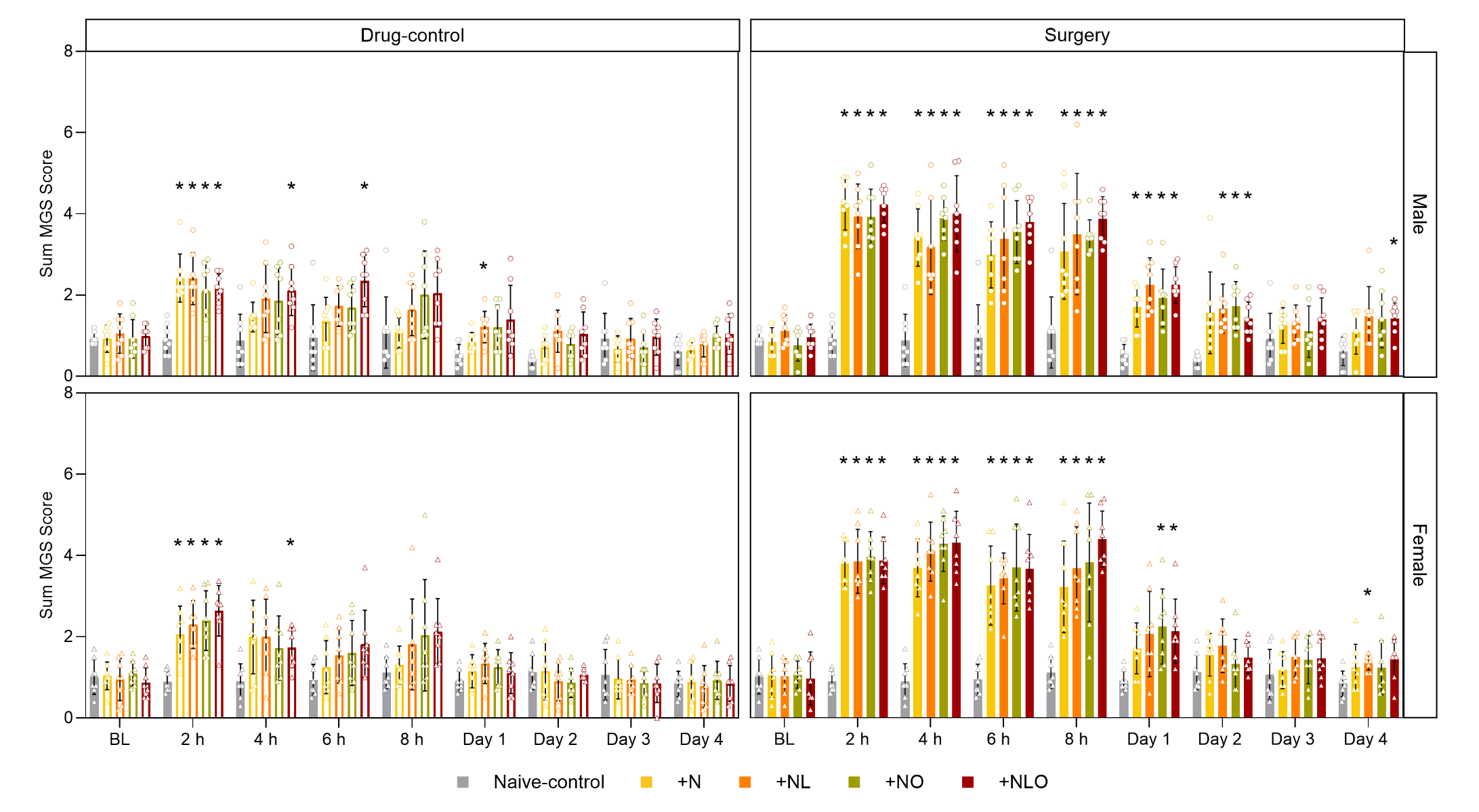


**Supplementary figure S3: Activity**

During the first 20 h following surgery, the distance moved (**a**) in PhenoTyper home cages was significantly reduced in all groups with the different analgesic regimens. In contrast, the mean velocity (**b**) was only reduced in +NL, +NLO males and +NL, +NO, +NLO females (group sizes n = 8). No differences in the Voluntary Wheel Running activity (**c**) were observed in 12-h of light phases (☼) and dark phases (🌙) (group sizes differing from n = 8: males day 4 light/dark surgery +N n = 6 and all other groups n = 7).(* = p < 0.05; analgesic regimen color-coded in (**c**); mean ± SD in (**a**), (**b**), and (**c**); One-way ANOVA in (**a**) and (**b**), Two-way RM ANOVA/ Mixed effects model with Bonferroni *post hoc* test in (**c**)).

**
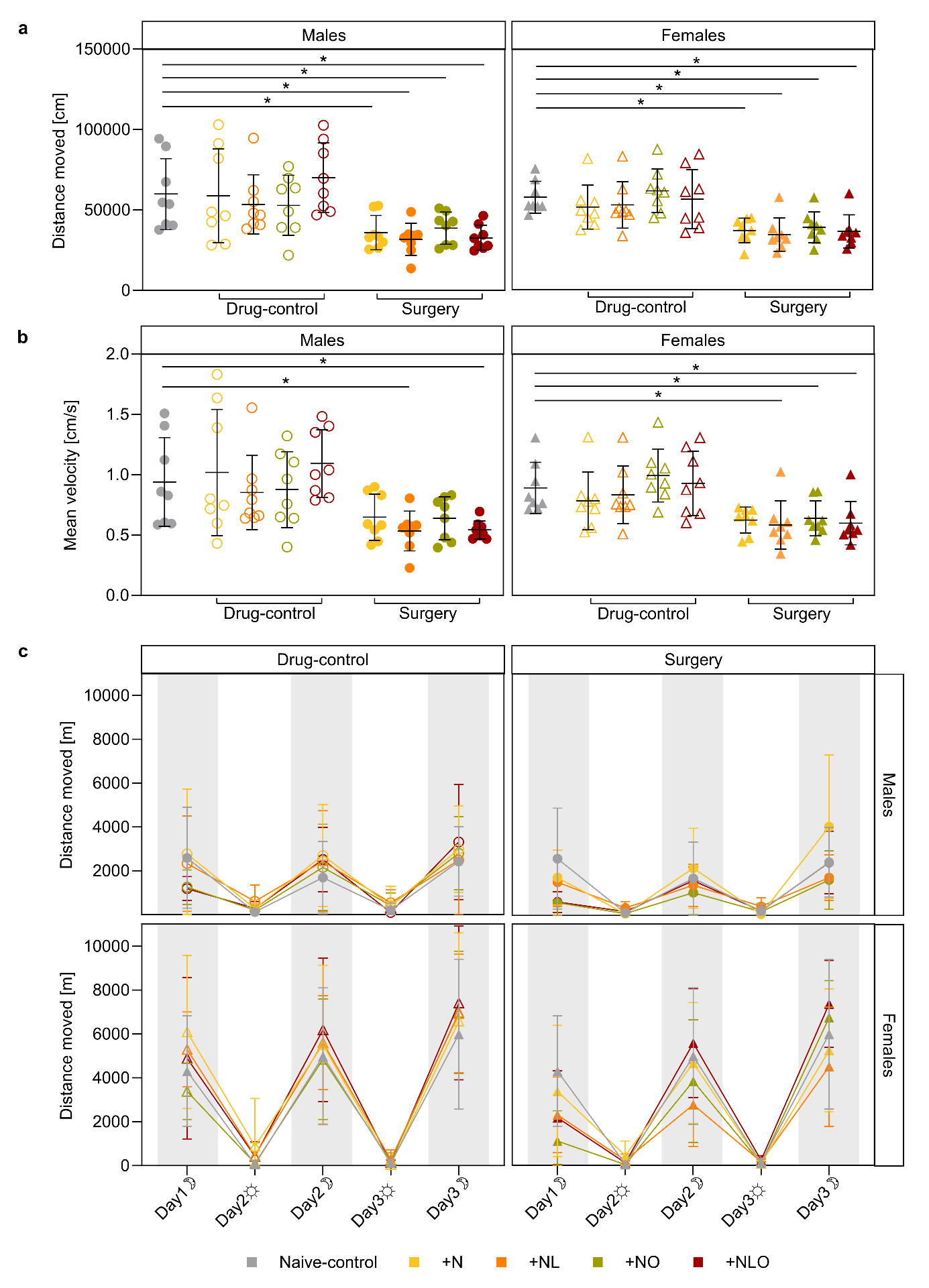
**

**Supplementary figure S4: Home cage-based behavior**

The baseline nest complexity of male and female mice is illustrated in (**a**) (group sizes n = 8). Burrowing behavior measured by weight of burrowed pellets after surgery or anesthesia is illustrated in (**b**). The difference to the baseline performance is illustrated as delta (▲)-burrowing in (**c**) (group size n= 8). Taking the baseline values into account, the delta (▲)-latency to the onset of burrowing is shown in (**d**) (group sizes differing from n = 8: males drug-control +NO n = 7).(* = p < 0.05; analgesic regimen color-coded in (**a**), (**b**), and (**c**); median ± IQR in (**a**), mean ± SD in (**b**), (**c**), and (**d**); Kruskal Wallis Test with Dunns *post hoc* in (**a**), One-way ANOVA in (**d**), Two-way RM ANOVA/ Mixed effects model with Bonferroni *post hoc* test in (**b**) and (**c**)).

**
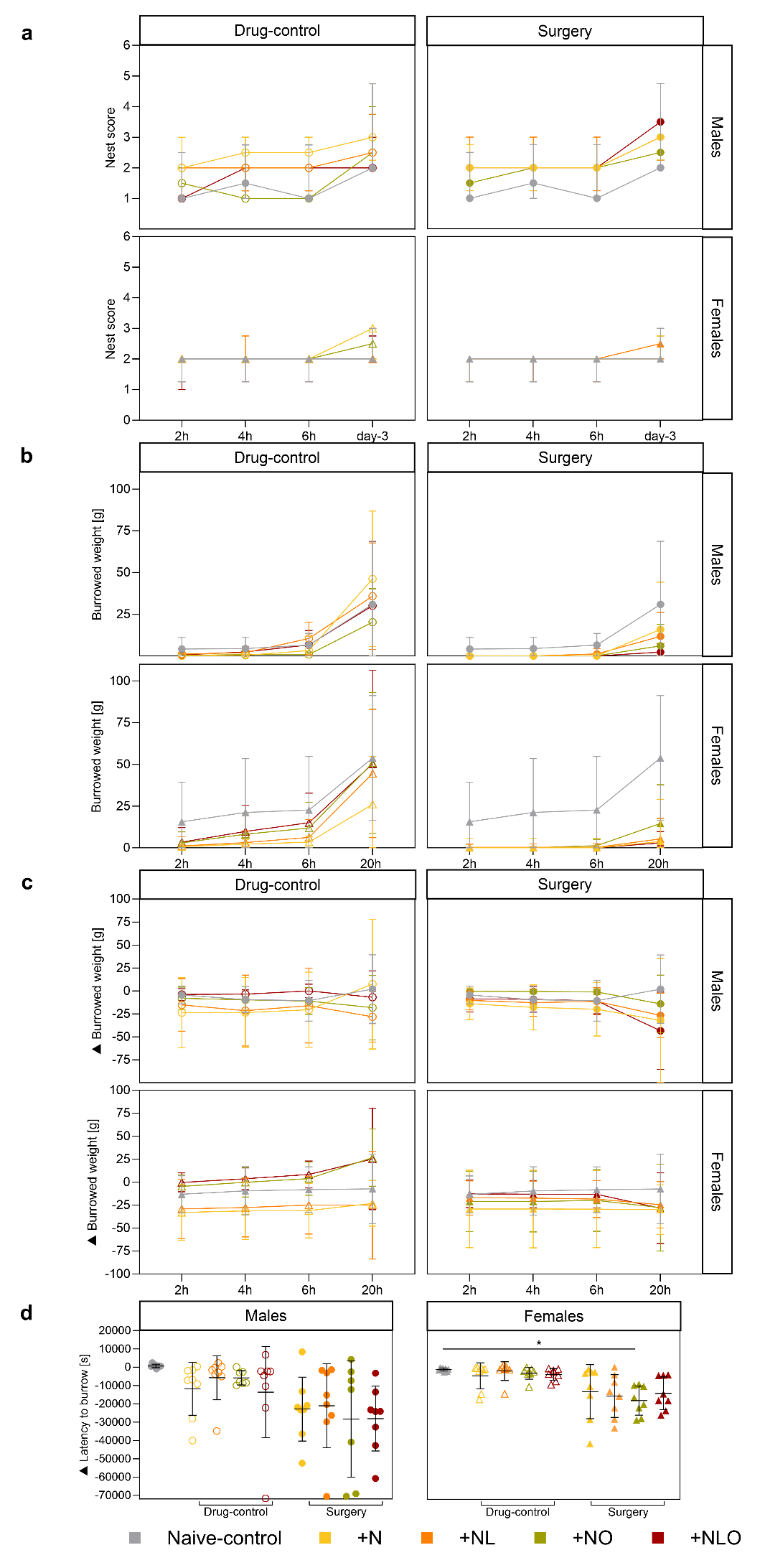
**

**Supplementary figure S5: Body weight and FCMs**

Absolute body weight data from male and female mice are shown in (**a**) (group size differing from n = 8: males: day 4 +N surgery n = 7, females: 6 h +NL drug-control and day -4 naïve-control n = 7). Baseline measurements of the fecal corticosterone metabolites are illustrated for females on day -6 (group-caged) (**b**) and males and females (single-caged) on day -3 (**c**) (group size differing from n = 8: females: day -6 +N surgery and drug-control n = 7). FCM concentrations at the day of euthanasia (day 4) are shown in (**d**) (group size differing from n = 8: males day 4 +N surgery n = 7). (* = p < 0.05; analgesic regimen color-coded in (**a**), mean ± SD in (**a**), (**b**), (**c**), and (**d**); One-way ANOVA in (**b**), (**c**), and (**d**), Two-way RM ANOVA/ Mixed effects model with Bonferroni *post hoc* test in (**a**)).

**
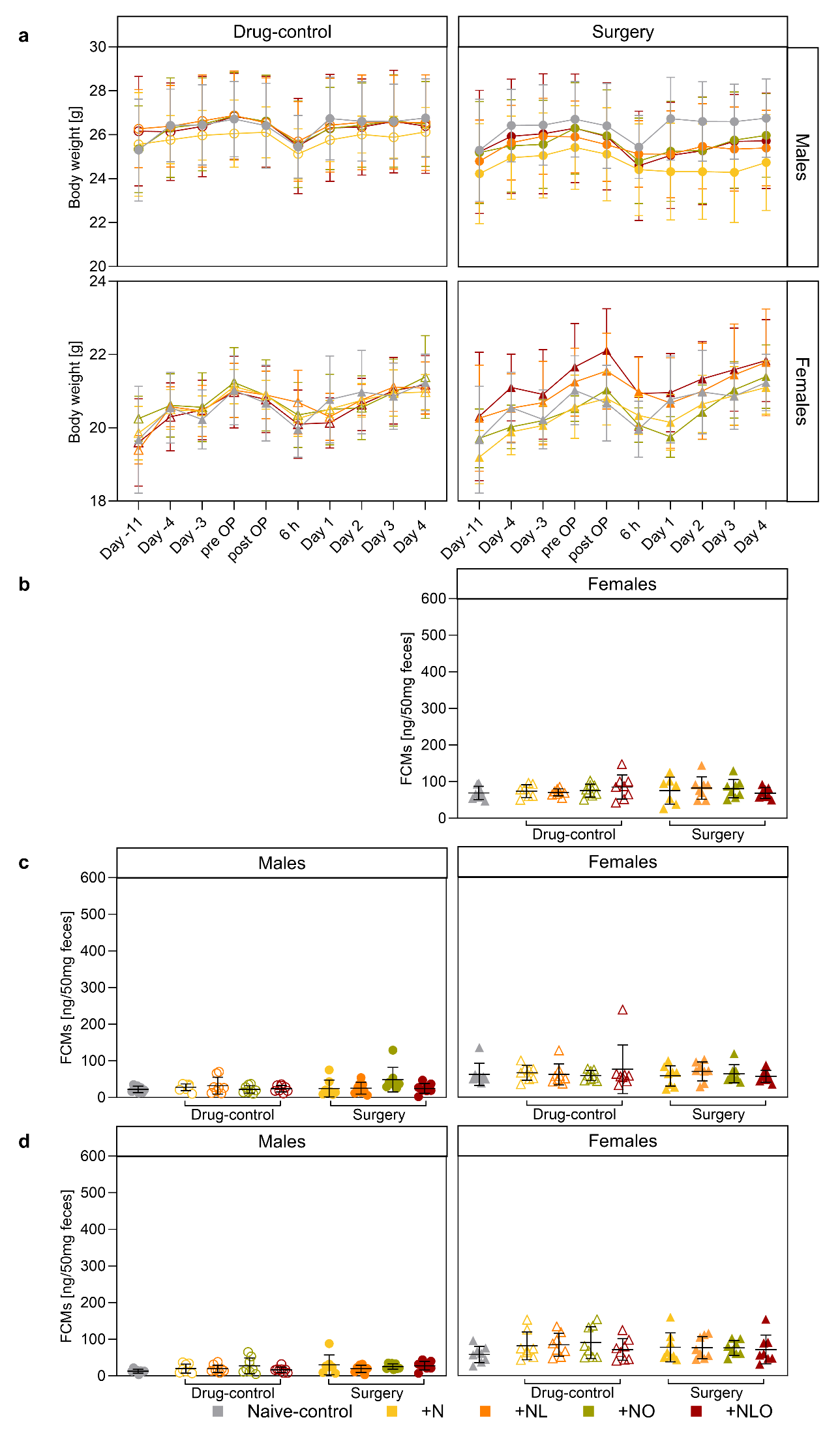
**

**Supplementary figure S6: Spearman correation**

Results of Spearman correlation analysis illustrated in a heat map. The data set comprises selected parameters from males (**a**) and females (**b**) on day 1. See the table S11 for explanation of parameters.


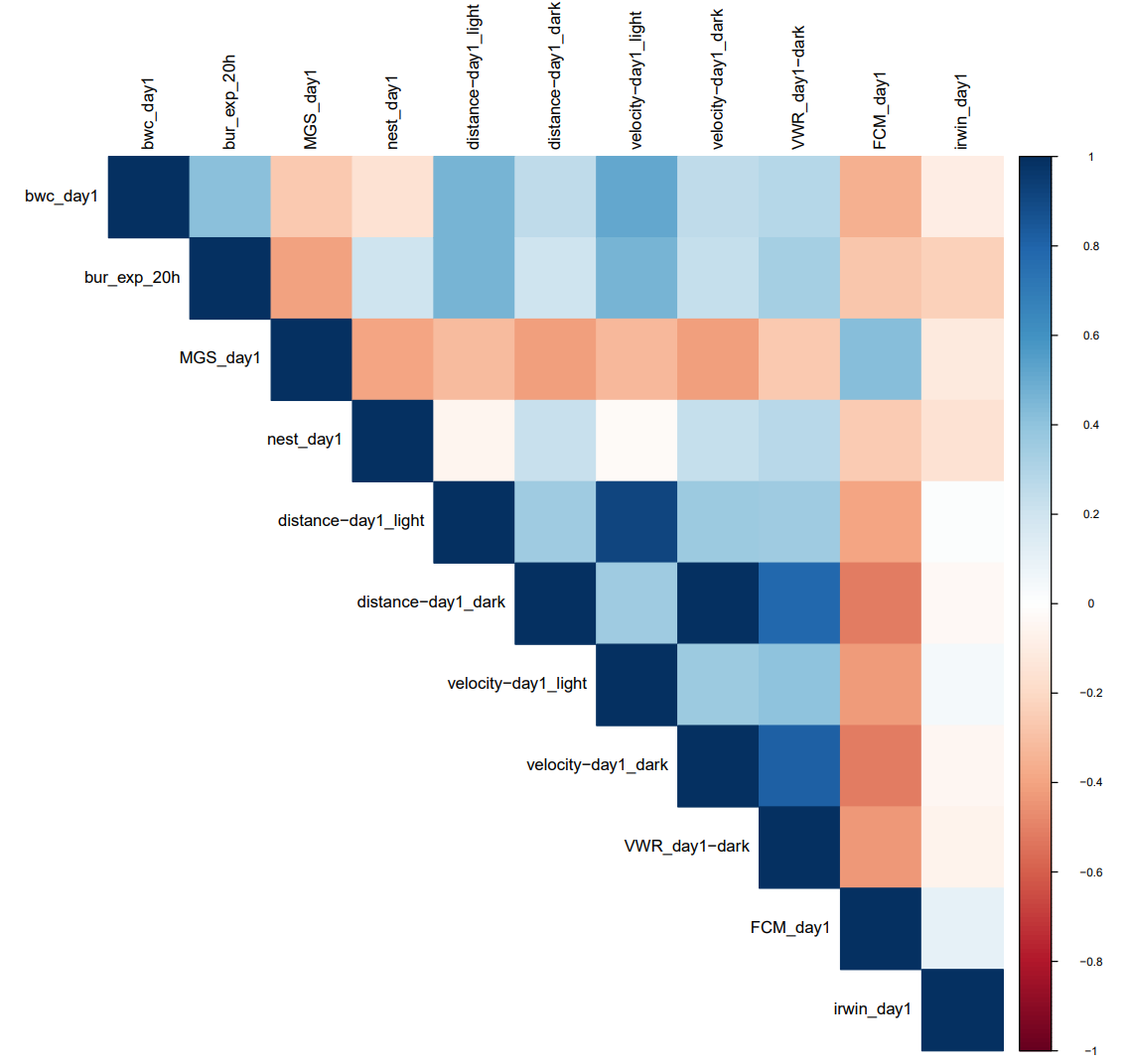

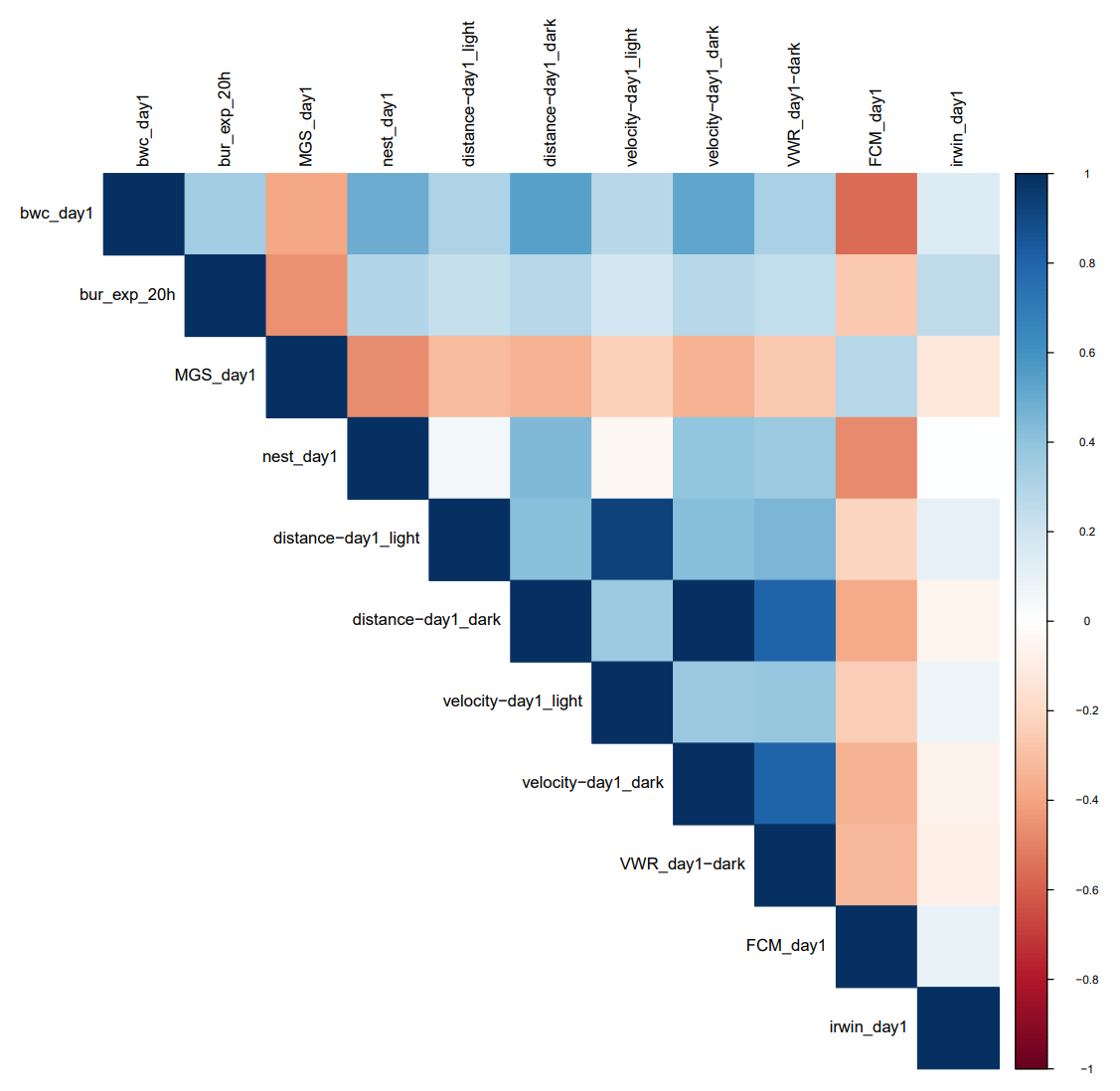


**a**

**b**


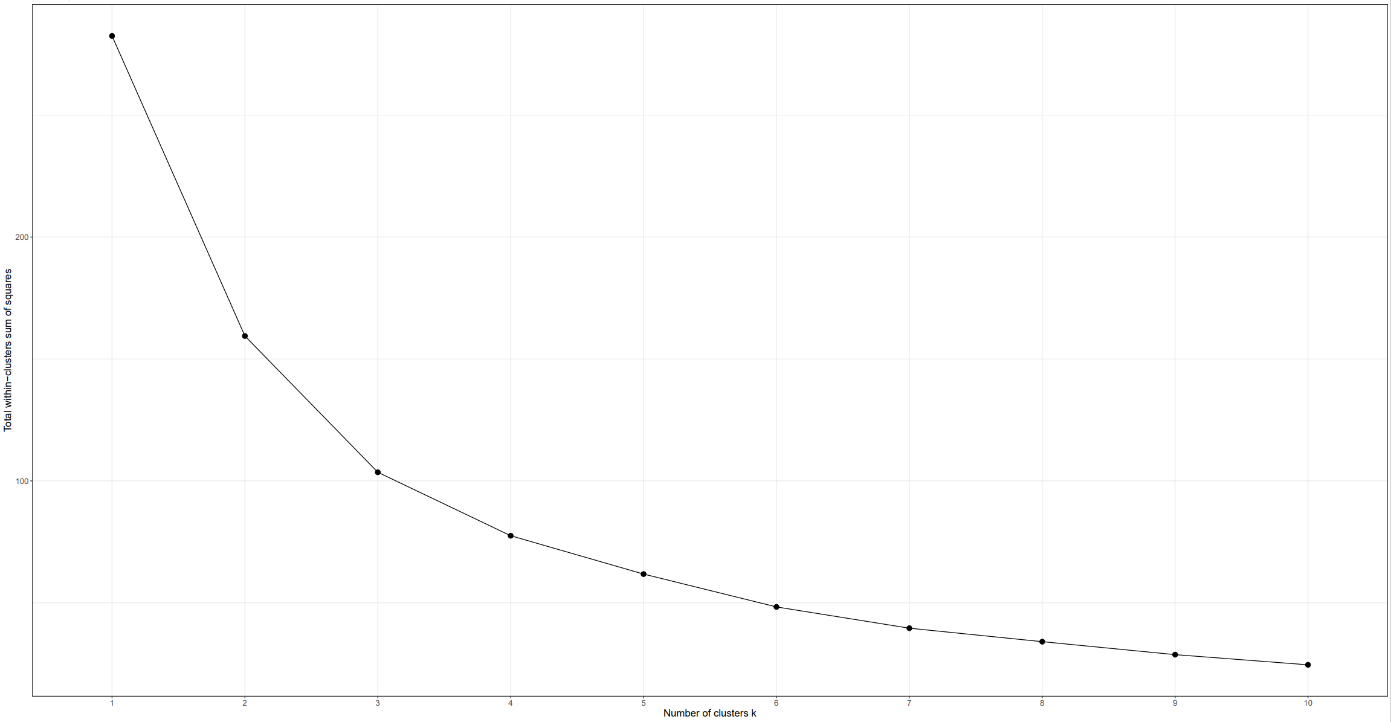


**a**

**b**


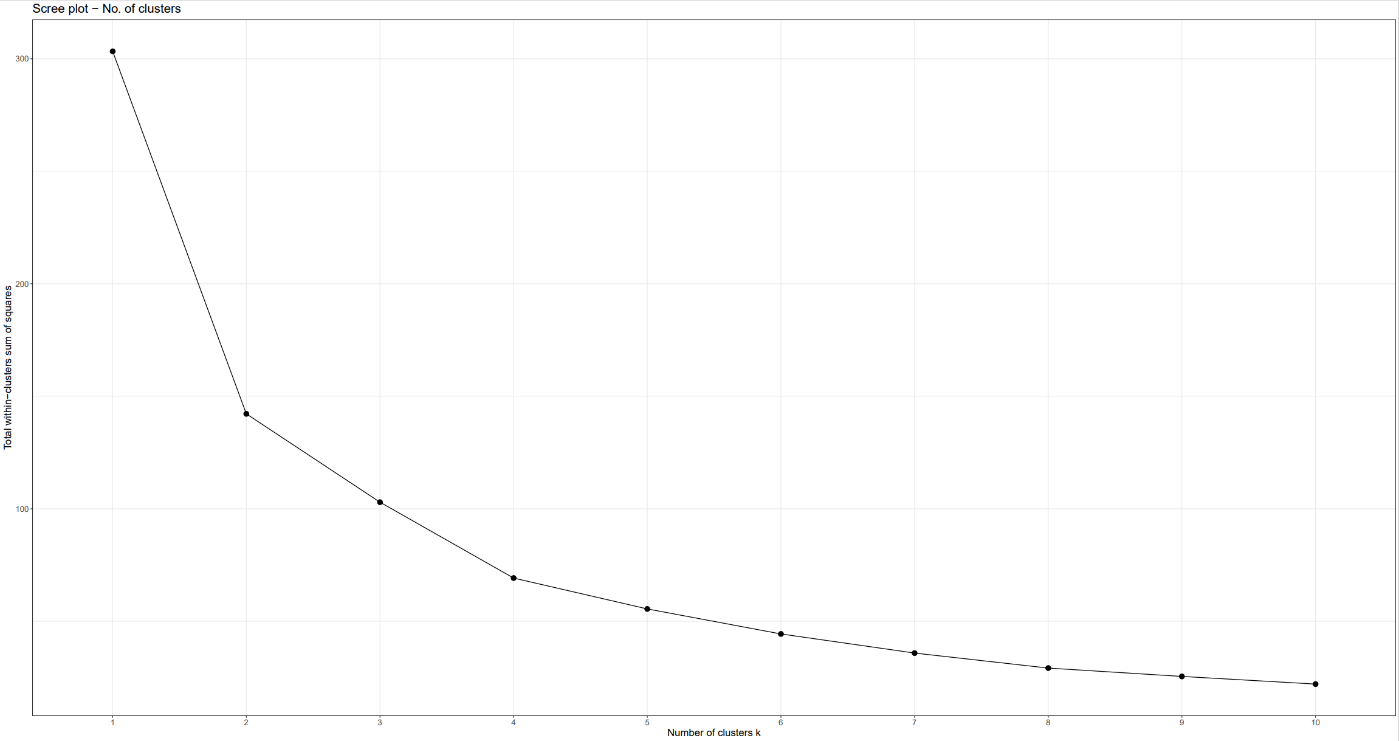


**Supplementary Figure S7: Scree plot**

Scree plot of the within-cluster sum of squares to determine the number of clusters *k* of male (**a**) and female (**b**) data on day 1.

1. **Supplementary Tables**

**Supplementary table 1: Descriptive statistics pilot study**

|  | | **A** | | | | **B** | | | |
| --- | --- | --- | --- | --- | --- | --- | --- | --- | --- |
|  |  | **Males** | | **Females** | | **Males** | | **Females** | |
| **Parameter** | **Time point** | **Mean** | **SD** | **Mean** | **SD** | **Mean** | **SD** | **Mean** | **SD** |
| Body weight [g] | BL | 27.67 | 4.15 | 21.20 | 0.92 | 28.23 | 0.15 | 21.17 | 0.65 |
|  | BL | 27.27 | 3.98 | 20.70 | 1.47 | 27.90 | 0.30 | 21.73 | 0.61 |
|  | Pre-OP | 28.00 | 4.52 | 21.70 | 1.57 | 28.63 | 0.68 | 22.07 | 0.50 |
|  | Post-OP | 28.17 | 3.96 | 21.57 | 1.62 | 28.70 | 0.70 | 22.53 | 0.38 |
|  | 6 h | 26.60 | 4.36 | 21.17 | 1.53 | 27.13 | 0.51 | 21.60 | 0.14 |
|  | Day 1 | 26.93 | 4.74 | 20.47 | 1.10 | 26.97 | 1.47 | 19.73 | 0.51 |
|  | Day 2 | 27.07 | 4.04 | 20.53 | 0.64 | 27.07 | 0.40 | 18.60 | 0.80 |
|  | Day 3 | 27.43 | 3.84 | 21.40 | 0.61 | 28.10 | 1.10 | 19.62 | 1.73 |
|  | Day 4 | 27.83 | 4.02 | 21.90 | 0.56 | 29.10 | 0.69 | 21.60 | 1.18 |
| Mean MGS | BL | 0.11 | 0.02 | 0.09 | 0.01 | 0.10 | 0.04 | 0.12 | 0.04 |
|  | 2 h | 1.01 | 0.21 | 1.12 | 0.06 | 0.96 | 0.09 | 0.99 | 0.11 |
|  | 4 h | 0.99 | 0.15 | 0.98 | 0.07 | 0.77 | 0.25 | 0.99 | 0.22 |
|  | 6 h | 0.95 | 0.03 | 0.77 | 0.24 | 0.94 | 0.09 | 1.02 | 0.25 |
|  | 8 h | 0.97 | 0.22 | 0.94 | 0.08 | 0.86 | 0.12 | 0.90 | 0.04 |
|  | Day 1 | 0.49 | 0.00 | 0.65 | 0.32 | 0.62 | 0.09 | 0.53 | 0.14 |
|  | Day 2 | 0.35 | 0.10 | 0.50 | 0.23 | 0.44 | 0.02 | 0.53 | 0.07 |
|  | Day 3 | 0.37 | 0.11 | 0.26 | 0.09 | 0.36 | 0.05 | 0.41 | 0.12 |
|  | Day 4 | 0.35 | 0.03 | 0.43 | 0.10 | 0.35 | 0.11 | 0.36 | 0.06 |
| Burrowing [g] | BL_2h | 12.57 | 20.05 | 1.93 | 3.35 | 0.00 | 0.20 | 1.00 | 1.39 |
|  | BL_4h | 12.47 | 19.96 | 1.97 | 3.50 | 1.20 | 0.95 | 0.93 | 1.37 |
|  | BL_6h | 12.37 | 20.04 | 2.63 | 3.04 | 1.07 | 0.93 | 1.73 | 3.00 |
|  | BL_20h | 27.80 | 28.71 | 25.70 | 11.87 | 18.77 | 19.91 | 34.30 | 19.35 |
|  | exp_2h | 0.17 | 0.21 | -0.03 | 0.06 | 0.17 | 0.21 | -0.23 | 0.06 |
|  | exp_4h | -0.07 | 0.21 | -1.10 | 1.82 | 0.17 | 0.47 | -0.17 | 0.06 |
|  | exp_6h | 0.13 | 0.25 | -1.13 | 1.97 | 0.30 | 0.53 | -0.10 | 0.17 |
|  | exp_20h | 10.47 | 18.74 | 21.27 | 37.72 | 0.83 | 0.76 | 6.23 | 11.10 |
| Nest score # | BL_2h | 2 |  | 1 |  | 3 |  | 1 |  |
|  | BL_4h | 3 |  | 2 |  | 3 |  | 2 |  |
|  | BL_6h | 3 |  | 2 |  | 3 |  | 2 |  |
|  | BL_20h | 3 |  | 3 |  | 4 |  | 2 |  |
|  | exp_2h | 1 |  | 1 |  | 1 |  | 1 |  |
|  | exp_4h | 1 |  | 1 |  | 1 |  | 1 |  |
|  | exp_6h | 1 |  | 1 |  | 1 |  | 1 |  |
|  | Day 1 | 3 |  | 2 |  | 1 |  | 1 |  |
|  | Day 2 | 4 |  | 4 |  | 3 |  | 2 |  |
|  | Day 3 | 4 |  | 5 |  | 4 |  | 3 |  |
|  | Day 4 | 5 |  | 5 |  | 5 |  | 4 |  |
| Sum  Neuroscore # | BL | 2 |  | 1 |  | 5 |  | 4 |  |
|  | 4h | 3 |  | 3 |  | 3 |  | 5 |  |
|  | Day 1 | 1 |  | 2 |  | 2 |  | 2 |  |
|  | Day 2 | 1 |  | 2 |  | 2 |  | 2 |  |
|  | Day 3 | 1 |  | 1 |  | 1 |  | 1 |  |
|  | Day 4 | 1 |  | 1 |  | 0 |  | 1 |  |
| PT distance moved [cm] | 20 h | 36321.3 | 6428.2 | 41276.5 | 5253.8 | 36478.2 | 1373.7 | 32492.7 | 2188.8 |
|  | Day 0 dark | 19217.8 | 4695.2 | 23422.1 | 6983.5 | 23437.3 | 4468.7 | 18341.6 | 1851.5 |
|  | Day 1 dark | 27463.3 | 368.9 | 31510.6 | 8954.1 | 27550.9 | 15344.0 | 74570.5 | 11499.9 |
|  | Day 2 light | 15167.9 | 5752.0 | 10737.6 | 957.1 | 10323.4 | 871.3 | 23812.6 | 13155.8 |
|  | Day 2 dark | 64197.7 | 41532.0 | 34870.1 | 8400.7 | 41736.4 | 8948.8 | 54487.6 | 17570.3 |
|  | Day 3 light | 12408.1 | 3925.7 | 11506.4 | 658.1 | 10589.7 | 1780.2 | 13715.5 | 3076.0 |
|  | Day 3 dark | 52651.8 | 13932.2 | 35334.8 | 9075.9 | 59775.2 | 26504.0 | 45481.4 | 31934.6 |
| PT VWR [m] | Day 1 dark | 296.3 | 112.5 | 481.6 | 681.1 | 475.7 | 615.5 | 2736.5 | 904.0 |
|  | Day 2 light | 116.2 | 59.7 | 52.5 | 74.3 | 8.5 | 0.7 | 968.2 | 931.4 |
|  | Day 2 dark | 2134.4 | 2462.1 | 695.5 | 983.7 | 1422.0 | 1454.5 | 2526.8 | 1129.3 |
|  | Day 3 light | 208.6 | 262.7 | 41.5 | 58.6 | 67.9 | 74.0 | 186.3 | 85.9 |
|  | Day 3 dark | 2481.9 | 1495.7 | 620.4 | 877.4 | 3096.3 | 3245.9 | 2499.9 | 2594.4 |
| PT duration in zone house [s] | 20 h | 47285.7 | 10761.3 | 25994.4 | 23901.5 | 22294.4 | 9644.0 | 44118.4 | 6917.2 |
|  | Day 0 dark | 28090.0 | 5261.0 | 14917.1 | 14703.4 | 11899.0 | 9539.9 | 27725.8 | 3962.3 |
|  | Day 1 dark | 24242.4 | 5284.7 | 25970.8 | 4954.4 | 25123.4 | 7904.3 | 22334.9 | 3368.7 |
|  | Day 2 light | 32807.3 | 2016.6 | 33254.9 | 1987.6 | 33839.6 | 414.4 | 28653.4 | 6079.2 |
|  | Day 2 dark | 18004.9 | 7677.3 | 23126.4 | 6419.3 | 23638.2 | 2627.0 | 27212.5 | 3064.0 |
|  | Day 3 light | 34797.7 | 2429.8 | 35273.3 | 3247.5 | 34241.6 | 1755.6 | 28210.4 | 3342.4 |
|  | Day 3 dark | 16492.7 | 5248.2 | 23598.0 | 5580.3 | 15270.4 | 10212.5 | 27152.5 | 4222.3 |
| Liquid intake (cor) [g] | Mean-BL | 5.07326 | 1.3388 | 4.64993 | 0.79186 | 4.56823 | 0.51498 | 5.22656 | 0.16394 |
|  | Day -1 | 4.78056 | 1.79258 | 4.61389 | 0.95394 |  |  |  |  |
|  | Day 0 | 2.42083 | 0.57735 | 2.42963 | 2.58879 | 2.62963 | 2.46346 | 1.35417 | 0.55076 |
|  | Day 1 | 3.75417 | 1.30512 | 4.5963 | 2.27894 | 3.8963 | 2.37939 | 1.32083 | 0.23094 |
|  | Day 2 | 4.42083 | 0.75056 | 4.7963 | 1.56519 | 5.16296 | 1.08449 | 2.2875 | 2.72213 |
|  | Day 3 | 2.92083 | 0.9609 | 4.02963 | 0.9682 | 3.76296 | 0.92241 | 3.92083 | 2.65393 |
| Histopathological examination | After euthanasia |  | | n = 1 ulcerative lesion GIT | | n = 1 ulcerative lesion GIT; n = 1 macroscopic visible lesion at carprofen injection site | |  | |

n = 3 mice per group and treatment (for PT analysis A females and B males n =2; baseline neuroscore A male, A female, B female n = 2; 6 h body weight B female n = 2). # = median is reported, BL = measured at baseline time point (day -4 to day -3), cor = corrected for bottle drips, exp = measured during experimental phase (day 0 – day 4), GIT = gastrointestinal tract, OP = operation, PT = PhenoTyper home cage, SD = standard deviation.

**Supplementary table 2: Number of cases in which action units could not be evaluated**

^a^calculated per total number of analyzed pictures n= 13,055; ^b^calculated per total number of extracted pictures n = 13,130; ^c^the action unit ‘whisker change’ was not evaluable in all 10 pictures per mouse per time point, calculated per total MG-scoring cases (including all mice and time points) n = 1,296.

|  | **% of cases in which action units could not be evaluated^a^** | | | | | **% of rejected pictures^b^** | **% no whisker change^c^** |
| --- | --- | --- | --- | --- | --- | --- | --- |
|  | **Orbital tightening** | **Nose bulge** | **Cheek bulge** | **Ear position** | **Whisker change** |  |  |
| Total | 0.09 | 0.22 | 0.00 | 0.04 | 54.18 | 0.57 | 5.79 |
| Males | 0.05 | 0.18 | 0.00 | 0.05 | 45.67 | 0.32 | 2.16 |
| Females | 0.14 | 0.26 | 0.00 | 0.03 | 62.67 | 0.83 | 9.41 |

**Supplementary table 3: Statistical analyses of MGS**

| **Statistical test** | **Time point** | **Experimental group** | **Experimental group** | ***p*-value** |
| --- | --- | --- | --- | --- |
| **Mean MGS Score (see Fig. 2)** | | | | |
| **Two-way RM ANOVA** | **Males: Naive-control vs Drug-control**  **Time:** *F*_5.723, 200.3_ = 31.7, *p* < 0.0001  **Analgesic regimen:** *F*_4, 35_ = 9.62, *p* < 0.0001  **Time x analgesic regimen:** *F*_32, 280_ = 2.448, *p* < 0.0001 | | | |
| Bonferroni *post hoc* test | 2 h | Naive-control | Drug-control + N | 0.0117 |
|  | 2 h | Naive-control | Drug-control + NL | 0.0169 |
|  | 2 h | Naive-control | Drug-control + NO | 0.0283 |
|  | 2 h | Naive-control | Drug-control + NLO | 0.0033 |
|  | 4 h | Naive-control | Drug-control + NLO | 0.001 |
|  | 4 h | Drug-control + N | Drug-control + NLO | 0.0112 |
|  | 6 h | Naive-control | Drug-control + NLO | 0.0062 |
|  | 6 h | Drug-control + N | Drug-control + NLO | 0.0089 |
|  | 6 h | Drug-control + NL | Drug-control + NLO | 0.0268 |
|  | Day 1 | Naive-control | Drug-control + NL | 0.0052 |
| **Mixed-effects model** | **Males: Naive-control vs Surgery**  **Time:** *F*_5.672, 197.1_ = 103.1, *p* < 0.0001  **Analgesic regimen:** *F*_4, 35_ = 27.59, *p* < 0.0001  **Time x analgesic regimen:** *F*_32, 278_ = 6.798, *p* < 0.0001 | | | |
| Bonferroni *post hoc* test | 2 h | Naive-control | Surgery + N | < 0.0001 |
|  | 2 h | Naive-control | Surgery + NL | 0.0003 |
|  | 2 h | Naive-control | Surgery + NO | < 0.0001 |
|  | 2 h | Naive-control | Surgery + NLO | < 0.0001 |
|  | 4 h | Naive-control | Surgery + N | < 0.0001 |
|  | 4 h | Naive-control | Surgery + NL | 0.0011 |
|  | 4 h | Naive-control | Surgery + NO | < 0.0001 |
|  | 4 h | Naive-control | Surgery + NLO | < 0.0001 |
|  | 6 h | Naive-control | Surgery + N | O.0031 |
|  | 6 h | Naive-control | Surgery + NL | 0.0069 |
|  | 6 h | Naive-control | Surgery + NO | < 0.0001 |
|  | 6 h | Naive-control | Surgery + NLO | < 0.0001 |
|  | 8 h | Naive-control | Surgery + N | 0.0161 |
|  | 8 h | Naive-control | Surgery + NL | 0.0107 |
|  | 8 h | Naive-control | Surgery + NO | 0.0003 |
|  | 8 h | Naive-control | Surgery + NLO | < 0.0001 |
|  | Day 1 | Naive-control | Surgery + N | 0.0004 |
|  | Day 1 | Naive-control | Surgery + NL | < 0.0001 |
|  | Day 1 | Naive-control | Surgery + NO | 0.0022 |
|  | Day 1 | Naive-control | Surgery + NLO | < 0.0001 |
|  | Day 2 | Naive-control | Surgery + NL | 0.0179 |
|  | Day 2 | Naive-control | Surgery + NO | 0.001 |
|  | Day 2 | Naive-control | Surgery + NLO | 0.0066 |
| **Two-way RM ANOVA** | **Females: Naive-control vs Drug-control**  **Time:** *F*_5.147, 180.1_ = 20.5, *p* < 0.0001  **Time x analgesic regimen:** *F*_32, 280_ = 2.613, *p* < 0.0001 | | | |
| Bonferroni *post hoc* test | 2 h | Naive-control | Drug-control + N | 0.0398 |
|  | 2 h | Naive-control | Drug-control + NL | 0.0009 |
|  | 2 h | Naive-control | Drug-control + NO | 0.0015 |
|  | 2 h | Naive-control | Drug-control + NLO | 0.0021 |
|  | 4 h | Naive-control | Drug-control + N | 0.0417 |
|  | 4 h | Naive-control | Drug-control + NLO | 0.0101 |
| **Two-way RM ANOVA** | **Females: Naive-control vs Surgery**  **Time:** *F*_2.748, 19.24_ = 94.06, *p* < 0.0001  **Analgesic regimen:** *F*_2.766, 19.36_ = 29.54, *p* < 0.0001  **Time x analgesic regimen:** *F*_5.925, 41.48_ = 8.327, *p* < 0.0001 | | | |
| Bonferroni *post hoc* test | 2 h | Naive-control | Surgery + N | < 0.0001 |
|  | 2 h | Naive-control | Surgery + NL | < 0.0001 |
|  | 2 h | Naive-control | Surgery + NO | < 0.0001 |
|  | 2 h | Naive-control | Surgery + NLO | < 0.0001 |
|  | 4 h | Naive-control | Surgery + N | < 0.0001 |
|  | 4 h | Naive-control | Surgery + NL | < 0.0001 |
|  | 4 h | Naive-control | Surgery + NO | < 0.0001 |
|  | 4 h | Naive-control | Surgery + NLO | < 0.0001 |
|  | 6 h | Naive-control | Surgery + N | 0.0197 |
|  | 6 h | Naive-control | Surgery + NL | 0.0015 |
|  | 6 h | Naive-control | Surgery + NO | 0.0052 |
|  | 6 h | Naive-control | Surgery + NLO | < 0.0001 |
|  | 8 h | Naive-control | Surgery + N | 0.006 |
|  | 8 h | Naive-control | Surgery + NL | 0.0111 |
|  | 8 h | Naive-control | Surgery + NO | 0.0143 |
|  | 8 h | Naive-control | Surgery + NLO | 0.0002 |
|  | Day 1 | Naive-control | Surgery + N | 0.0315 |
|  | Day 1 | Naive-control | Surgery + NO | 0.0048 |
|  | Day 1 | Naive-control | Surgery + NLO | 0.0072 |
|  | Day 4 | Naive-control | Surgery + NL | 0.0319 |
| **Sum MGS Score (see Fig. S1)** | | | | |
| **Mixed-effects model** | **Males: Naive-control vs Drug-control**  **Time:** *F*_5.463, 185.1_ = 30.2, *p* < 0.0001  **Analgesic regimen:** *F*_4, 35_ = 9.404, *p* < 0.0001  **Time x analgesic regimen:** *F*_32, 271_ = 2.529, *p* < 0.0001 | | | |
| Bonferroni *post hoc* test | 2 h | Naive-control | Drug-control + N | 0.0368 |
|  | 2 h | Naive-control | Drug-control + NL | 0.0297 |
|  | 2 h | Naive-control | Drug-control + NO | 0.0222 |
|  | 2 h | Naive-control | Drug-control + NLO | 0.0033 |
|  | 4 h | Naive-control | Drug-control + NLO | 0.001 |
|  | 4 h | Drug-control + N | Drug-control + NLO | 0.0112 |
|  | 6 h | Naive-control | Drug-control + NLO | 0.0062 |
|  | 6 h | Drug-control + N | Drug-control + NLO | 0.0089 |
|  | 6 h | Drug-control + NL | Drug-control + NLO | 0.0478 |
|  | Day 1 | Naive-control | Drug-control + NL | 0.0052 |
| **Mixed-effects model** | **Males: Naive-control vs Surgery**  **Time:** *F*_5.831, 196.8_ = 108.6, *p* < 0.0001  **Analgesic regimen:** *F*_4, 35_ = 27.45, *p* < 0.0001  **Time x analgesic regimen:** *F*_32, 270_ = 7.295, *p* < 0.0001 | | | |
| Bonferroni *post hoc* test | 2 h | Naive-control | Surgery + N | < 0.0001 |
|  | 2 h | Naive-control | Surgery + NL | < 0.0001 |
|  | 2 h | Naive-control | Surgery + NO | 0.0002 |
|  | 2 h | Naive-control | Surgery + NLO | < 0.0001 |
|  | 4 h | Naive-control | Surgery + N | < 0.0001 |
|  | 4 h | Naive-control | Surgery + NL | 0.0011 |
|  | 4 h | Naive-control | Surgery + NO | < 0.0001 |
|  | 4 h | Naive-control | Surgery + NLO | < 0.0001 |
|  | 6 h | Naive-control | Surgery + N | O.0031 |
|  | 6 h | Naive-control | Surgery + NL | 0.0069 |
|  | 6 h | Naive-control | Surgery + NO | 0.0002 |
|  | 6 h | Naive-control | Surgery + NLO | < 0.0001 |
|  | 8 h | Naive-control | Surgery + N | 0.0161 |
|  | 8 h | Naive-control | Surgery + NL | 0.013 |
|  | 8 h | Naive-control | Surgery + NO | 0.0003 |
|  | 8 h | Naive-control | Surgery + NLO | < 0.0001 |
|  | Day 1 | Naive-control | Surgery + N | 0.0004 |
|  | Day 1 | Naive-control | Surgery + NL | < 0.0001 |
|  | Day 1 | Naive-control | Surgery + NLO | < 0.0001 |
|  | Day 2 | Naive-control | Surgery + NL | 0.0179 |
|  | Day 2 | Naive-control | Surgery + NO | 0.0036 |
|  | Day 2 | Naive-control | Surgery + NLO | 0.0066 |
| **Mixed-effects model** | **Females: Naive-control vs Drug-control**  **Time:** *F*_4.657, 142.1_ = 21.45, *p* < 0.0001  **Analgesia:** *F*_4, 35_ = 2.497, *p* = 0.0604  **Time x analgesic regimen:** *F*_32, 244_ = 2.753, *p* < 0.0001 | | | |
| Bonferroni *post hoc* test | 2 h | Naive-control | Drug-control + NL | 0.0037 |
|  | 2 h | Naive-control | Drug-control + NO | 0.0013 |
|  | 2 h | Naive-control | Drug-control + NLO | 0.0481 |
|  | 4 h | Naive-control | Drug-control + N | 0.0194 |
|  | 4 h | Naive-control | Drug-control + NLO | 0.0035 |
| **Mixed-effects model** | **Females: Naive-control vs Surgery**  **Time:** *F*_5.547, 174.0_ = 94.97, *p* < 0.0001  **Analgesic regimen:** *F*_4, 35_ = 25.05, *p* < 0.0001  **Time x analgesic regimen:** *F*_32, 251_ = 7.544, *p* < 0.0001 | | | |
| Bonferroni *post hoc* test | 2 h | Naive-control | Surgery + N | < 0.0001 |
|  | 2 h | Naive-control | Surgery + NO | < 0.0001 |
|  | 2 h | Naive-control | Surgery + NLO | 0.0053 |
|  | 4 h | Naive-control | Surgery + N | 0.001 |
|  | 4 h | Naive-control | Surgery + NL | 0.0006 |
|  | 4 h | Naive-control | Surgery + NO | 0.0006 |
|  | 4 h | Naive-control | Surgery + NLO | < 0.0001 |
|  | 6 h | Naive-control | Surgery + N | 0.006 |
|  | 6 h | Naive-control | Surgery + NL | < 0.0001 |
|  | 6 h | Naive-control | Surgery + NO | 0.0003 |
|  | 6 h | Naive-control | Surgery + NLO | < 0.0001 |
|  | 8 h | Naive-control | Surgery + N | 0.0083 |
|  | 8 h | Naive-control | Surgery + NL | 0.0017 |
|  | 8 h | Naive-control | Surgery + NO | 0.0023 |
|  | 8 h | Naive-control | Surgery + NLO | < 0.0001 |
|  | Day 1 | Naive-control | Surgery + N | 0.0103 |
|  | Day 1 | Naive-control | Surgery + NO | 0.0085 |
|  | Day 1 | Naive-control | Surgery + NLO | 0.0130 |
|  | Day 4 | Naive-control | Surgery + NL | 0.0456 |
| **Sum MGS without whisker action unit (see Fig. S2)** | | | | |
| **Mixed-effects model** | **Males: Naive-control vs Drug-control**  **Time:** *F*_5.469, 190.0_ = 31.65, *p* < 0.0001  **Analgesic regimen:** *F*_4, 35_ = 9.728, *p* < 0.0001  **Time x analgesic regimen:** *F*_32, 278_ = 2.273, *p* < 0.0002 | | | |
| Bonferroni *post hoc* test | 2 h | Naive-control | Drug-control + N | 0.0006 |
|  | 2 h | Naive-control | Drug-control + NL | 0.0013 |
|  | 2 h | Naive-control | Drug-control + NO | 0.018 |
|  | 2 h | Naive-control | Drug-control + NLO | < 0.0001 |
|  | 4 h | Naive-control | Drug-control + NLO | 0.0161 |
|  | 6 h | Naive-control | Drug-control + NLO | 0.0203 |
|  | Day 1 | Naive-control | Drug-control + NL | 0.0125 |
| **Mixed-effects model** | **Males: Naive-control vs Surgery**  **Time:** *F*_5.337, 184.8_ = 127.2, *p* < 0.0001  **Analgesic regimen:** *F*_4, 35_ = 22.70, *p* < 0.0001  **Time x analgesic regimen:** *F*_32, 277_ = 7.451, *p* < 0.0001 | | | |
| Bonferroni *post hoc* test | 2 h | Naive-control | Surgery + N | < 0.0001 |
|  | 2 h | Naive-control | Surgery + NL | < 0.0001 |
|  | 2 h | Naive-control | Surgery + NO | < 0.0001 |
|  | 2 h | Naive-control | Surgery + NLO | < 0.0001 |
|  | 4 h | Naive-control | Surgery + N | < 0.0001 |
|  | 4 h | Naive-control | Surgery + NL | 0.0124 |
|  | 4 h | Naive-control | Surgery + NO | < 0.0001 |
|  | 4 h | Naive-control | Surgery + NLO | < 0.0001 |
|  | 6 h | Naive-control | Surgery + N | O.0019 |
|  | 6 h | Naive-control | Surgery + NL | 0.0069 |
|  | 6 h | Naive-control | Surgery + NO | 0.0001 |
|  | 6 h | Naive-control | Surgery + NLO | < 0.0001 |
|  | 8 h | Naive-control | Surgery + N | 0.0201 |
|  | 8 h | Naive-control | Surgery + NL | 0.0211 |
|  | 8 h | Naive-control | Surgery + NO | 0.0004 |
|  | 8 h | Naive-control | Surgery + NLO | < 0.0001 |
|  | Day 1 | Naive-control | Surgery + N | 0.0011 |
|  | Day 1 | Naive-control | Surgery + NL | 0.0007 |
|  | Day 1 | Naive-control | Surgery + NO | 0.0143 |
|  | Day 1 | Naive-control | Surgery + NLO | < 0.0001 |
|  | Day 2 | Naive-control | Surgery + NL | 0.005 |
|  | Day 2 | Naive-control | Surgery + NO | 0.0036 |
|  | Day 2 | Naive-control | Surgery + NLO | 0.0009 |
|  | Day 4 | Naive-control | Surgery + NLO | 0.0056 |
| **Two-way RM ANOVA** | **Females: Naive-control vs Drug-control**  **Time:** *F*_4.844, 169.5_ = 24.76, *p* < 0.0001  **Analgesia:** *F*_4, 35_ = 2.887, *p* = 0.0363  **Time x analgesic regimen:** *F*_32, 280_ = 2.533, *p* < 0.0001 | | | |
| Bonferroni *post hoc* test | 2 h | Naive-control | Drug-control + N | 0.018 |
|  | 2 h | Naive-control | Drug-control + NL | 0.0012 |
|  | 2 h | Naive-control | Drug-control + NO | 0.0045 |
|  | 2 h | Naive-control | Drug-control + NLO | 0.0004 |
|  | 4 h | Naive-control | Drug-control + NLO | 0.03 |
| **Two-way RM ANOVA** | **Females: Naive-control vs Surgery**  **Time:** *F*_5.577, 195.2_ = 113.4, *p* < 0.0001  **Analgesic regimen:** *F*_4, 35_ = 29.36, *p* < 0.0001  **Time x analgesic regimen:** *F*_32, 280_ = 7.992, *p* < 0.0001 | | | |
| Bonferroni *post hoc* test | 2 h | Naive-control | Surgery + N | < 0.0001 |
|  | 2 h | Naive-control | Surgery + NL | < 0.0001 |
|  | 2 h | Naive-control | Surgery + NO | < 0.0001 |
|  | 2 h | Naive-control | Surgery + NLO | < 0.0001 |
|  | 4 h | Naive-control | Surgery + N | < 0.0001 |
|  | 4 h | Naive-control | Surgery + NL | < 0.0001 |
|  | 4 h | Naive-control | Surgery + NO | < 0.0001 |
|  | 4 h | Naive-control | Surgery + NLO | < 0.0001 |
|  | 6 h | Naive-control | Surgery + N | 0.0014 |
|  | 6 h | Naive-control | Surgery + NL | < 0.0001 |
|  | 6 h | Naive-control | Surgery + NO | 0.0009 |
|  | 6 h | Naive-control | Surgery + NLO | < 0.0001 |
|  | 8 h | Naive-control | Surgery + N | 0.0083 |
|  | 8 h | Naive-control | Surgery + NL | 0.0009 |
|  | 8 h | Naive-control | Surgery + NO | 0.0097 |
|  | 8 h | Naive-control | Surgery + NLO | < 0.0001 |
|  | Day 1 | Naive-control | Surgery + NO | 0.0421 |
|  | Day 1 | Naive-control | Surgery + NLO | 0.02655 |
|  | Day 4 | Naive-control | Surgery + NL | 0.018 |

ANOVA = analysis of variance, RM = repeated measures, vs = versus, see Fig. 2, S1, S2.

**Supplementary table 4: Statistical analyses of activity parameters**

| **Statistical test** | **Time point** | **Experimental group** | **Experimental group** | ***p*-value** |
| --- | --- | --- | --- | --- |
| **Distance moved within the first 20 h after surgery [cm] (see Fig. S3a)** | | | | |
| **one-way ANOVA** | **Males: Naive-control vs Surgery**  **Analgesic regimen:** *F*_4, 35_ = 6.237, *p* = 0.0007 | | | |
| Bonferroni *post hoc* test | Within 20 h | Naive-control | Surgery + N | 0.0085 |
|  |  | Naive-control | Surgery + NL | 0.0013 |
|  |  | Naive-control | Surgery + NO | 0.0264 |
|  |  | Naive-control | Surgery + NLO | 0.002 |
| **one-way ANOVA** | **Females: Naive-control vs Surgery**  **Analgesic regimen:** *F*_4, 35_ = 7.772, *p* = 0.0001 | | | |
| Bonferroni *post hoc* test | Within 20 h | Naive-control | Surgery + N | 0.0014 |
|  |  | Naive-control | Surgery + NL | 0.0003 |
|  |  | Naive-control | Surgery + NO | 0.0045 |
|  |  | Naive-control | Surgery + NLO | 0.0009 |
| **Distance moved during the whole experimental phase [cm] (see Fig. 3a)** | | | | |
| **Mixed-effects model** | **Males: Naive-control vs Surgery**  **Time:** *F*_3.126, 102.6_ = 56.07, *p* < 0.0001  **Time x analgesic regimen:** *F*_24, 197_ = 2.213, *p* = 0.0016 | | | |
| **Two-way RM ANOVA** | **Females: Naive-control vs Surgery**  **Time:** *F*_3.301, 115.5_ = 151.0, *p* < 0.0001  **Analgesic regimen:** *F*_4, 35_ = 5.439, *p* = 0.0016  **Time x analgesic regimen:** *F*_24, 210_ = 3.803, *p* < 0.0001 | | | |
| Bonferroni *post hoc* test | Day0_dark | Naive-control | Surgery + N | 0.0494 |
|  | Day0_dark | Naive-control | Surgery + NL | 0.0417 |
|  | Day0_dark | Naive-control | Surgery + NO | 0.0198 |
|  | Day0_dark | Naive-control | Surgery + NLO | 0.0104 |
|  | Day1_dark | Naive-control | Surgery + NL | 0.0476 |
|  | Day1_dark | Naive-control | Surgery + NO | 0.0068 |
|  | Day1_dark | Naive-control | Surgery + NLO | 0.0439 |
|  | Day1_dark | Surgery + N | Surgery + NO | 0.0485 |
|  | Day2_dark | Surgery + N | Surgery + NL | 0.0193 |
|  | Day2_dark | Surgery + NL | Surgery + NLO | 0.0185 |
| **Velocity within the first 20 h after surgery [mean cm/s] (see Fig. S3b)** | | | | |
| **one-way ANOVA** | **Males: Naive-control vs Surgery**  **Analgesic regimen:** *F*_4, 35_ = 4.579, *p* = 0.0044 | | | |
| Bonferroni *post hoc* test | Within 20 h | Naive-control | Surgery + NL | 0.0067 |
|  |  | Naive-control | Surgery + NLO | 0.0086 |
| **one-way ANOVA** | **Females: Naive-control vs surgery**  **Analgesic regimen:** *F*_4, 35_ = 4.306, *p* = 0.0062 | | | |
| Bonferroni *post hoc* test | Within 20 h | Naive-control | Surgery + N | 0.0388 |
|  |  | Naive-control | Surgery + NL | 0.0114 |
|  |  | Naive-control | Surgery + NLO | 0.0177 |
| **VWR [m] (see Fig. S3c)** | | | | |
| **Mixed-effects model** | **Males: Naive-control vs Drug-control**  **Time:** *F*_2.066,_ 68.16 = 31.89, *p* < 0.0001 | | | |
| **Mixed-effects model** | **Males: Naive-control vs Surgery**  **Time:** *F*_1.986, 64.05_ = 40.7, *p* < 0.0001  **Time x analgesic regimen:** *F*_16, 129_ = 2.48, *p* = 0.0025 | | | |
| **Two-way RM ANOVA** | **Females: Naive-control vs Drug-control**  **Time:** *F*_2.292, 80.2_ = 96.88, *p* < 0.0001 | | | |
| **Two-way RM ANOVA** | **Females: Naive-control vs Surgery**  **Time:** *F*_2.607, 91.23_ = 95.45, *p* < 0.0001  **Time x analgesic regimen:** *F*_16, 140_ = 2.091, *p* = 0.0118 | | | |

ANOVA = analysis of variance, RM = repeated measures, vs = versus, see Fig. 3a, S3a-c.

**Supplementary table 5: Statistical analyses of nest complexity**

vs = versus, see Fig. 3b.

| **Statistical test** | **Time point** | **Experimental group** | **Experimental group** | ***p*-value** |
| --- | --- | --- | --- | --- |
| **Kruskal-Wallis Test** | 2 h | **Males: Naive-control vs Surgery**  *p* = 0.0013 | | |
| Dunn´s *post hoc* test |  | Naive-control | Surgery + NL | 0.0055 |
|  |  | Naive-control | Surgery + NO | 0.0055 |
|  |  | Naive-control | Surgery + NLO | 0.0055 |
| **Kruskal-Wallis Test** | 4 h | **Males: Naive-control vs Surgery**  *p* = 0.0058 | | |
| Dunn´s *post hoc* test |  | Naive-control | Surgery + NO | 0.0116 |
|  |  | Naive-control | Surgery + NLO | 0.0116 |
| **Kruskal-Wallis Test** | 6 h | **Males: Naive-control vs Surgery**  *p* = 0.0058 | | |
| Dunn´s *post hoc* test |  | Naive-control | Surgery + NO | 0.0116 |
|  |  | Naive-control | Surgery + NLO | 0.0116 |
| **Kruskal-Wallis Test** | 2 h | **Females: Naive-control vs Surgery**  *p* < 0.0001 | | |
| Dunn´s *post hoc* test |  | Naive-control | Surgery + N | 0.0003 |
|  |  | Naive-control | Surgery + NL | 0.0003 |
|  |  | Naive-control | Surgery + NO | 0.0003 |
|  |  | Naive-control | Surgery + NLO | 0.0003 |
| **Kruskal-Wallis Test** | 4 h | **Females: Naive-control vs Surgery**  *p* < 0.0001 | | |
| Dunn´s *post hoc* test |  | Naive-control | Surgery + N | 0.001 |
|  |  | Naive-control | Surgery + NL | 0.0116 |
|  |  | Naive-control | Surgery + NO | 0.001 |
|  |  | Naive-control | Surgery + NLO | 0.001 |
| **Kruskal-Wallis Test** | 6 h | **Females: Naive-control vs Surgery**  *p* < 0.0001 | | |
| Dunn´s *post hoc* test |  | Naive-control | Surgery + N | 0.0002 |
|  |  | Naive-control | Surgery + NL | 0.0021 |
|  |  | Naive-control | Surgery + NO | 0.0002 |
|  |  | Naive-control | Surgery + NLO | 0.0002 |
| **Kruskal-Wallis Test** | Day 1 | **Females: Naive-control vs Surgery**  *p* = 0.0025 | | |
| Dunn´s *post hoc* test |  | Naive-control | Surgery + NL | 0.002 |
|  |  | Naive-control | Surgery + NO | 0.0455 |
|  |  | Naive-control | Surgery + NLO | 0.032 |

**Supplementary table 6: Statistical analyses of burrowing behavior**

| **Statistical test** | | **Experimental group** | **Experimental group** | ***p*-value** |
| --- | --- | --- | --- | --- |
| **Latency to burrow [s] (see Fig. 3c)** | | | | |
| **one-way ANOVA** | **Males: Naive-control vs Surgery**  **Analgesic regimen:** *F*_4, 34_ = 4.359, *p* = 0.0059 | | | |
| Bonferroni *post hoc* test | | Naive-control | Surgery + NO | 0.0033 |
| **one-way ANOVA** | **Females: Naive-control vs Surgery**  **Analgesic regimen:** *F*_4, 35_ = 3.45, *p* = 0.0177 | | | |
| Bonferroni *post hoc* test | | Naive-control | Surgery + NO | 0.0187 |
| **▲-Latency to burrow [s] (see Fig. S4d)** | | | | |
| **one-way ANOVA** | **Males: Naive-control vs Surgery**  **Analgesic regimen:** *F*_4, 34_ = 2.724, *p* = 0.0454 | | | |
| **one-way ANOVA** | **Females: Naive-control vs Surgery**  **Analgesic regimen:** *F*_4, 35_ = 3.473, *p* = 0.0172 | | | |
| Bonferroni *post hoc* test | | Naive-control | Surgery + NO | 0.0172 |
| **Burrowed pellets [g] (see Fig. S4b)** | | | | |
| **two-way RM ANOVA** | **Males: Naive-control vs Drug-control**  **Time:** *F*_1.041, 7.290_ = 25.58, *p* = 0.0012 | | | |
| **two-way RM ANOVA** | **Males: Naive-control vs Surgery**  **Time:** *F*_1.013, 35.44_ = 11.54, *p* = 0.0016  **Analgesic regimen:** *F*_4, 35_ = 3.693, *p* = 0.0131 | | | |
| **two-way RM ANOVA** | **Females: Naive-control vs Drug-control**  **Time:** *F*_1.119, 39.18_ = 39.12, *p* < 0.0001 | | | |
| **two-way RM ANOVA** | **Females: Naive-control vs Surgery**  **Time:** *F*_1.147, 40.15_ = 19.44, *p* < 0.0001  **Analgesic regimen:** *F*_4, 35_ = 5.532, *p* = 0.0015  **Time x analgesic regimen:** *F*_12, 105_ = 3.905, *p* < 0.0001 | | | |
| **▲-Burrowed pellets [g] (see Fig. S4c)** | | | | |
| **two-way RM ANOVA** | **Females: Naive-control vs Drug-control**  **Analgesic regimen:** *F*_4, 35_ = 5.086, *p* = 0.0024 | | | |

ANOVA = analysis of variance, RM = repeated measures, vs = versus, see Fig. 3c, S4b-c.

**Supplementary table 7: Statistical analyses of body weight**

ANOVA = analysis of variance, RM = repeated measures, vs = versus, see Fig. 4a, S5a.

| **Statistical test** | **Time point** | **Experimental group** | **Experimental group** | ***p*-value** |
| --- | --- | --- | --- | --- |
| **Body weight change [%] (see Fig. 4a)** | | | | |
| **two-way RM ANOVA** | **Males: Naive-control vs Drug-control**  **Time:** *F*_3.650, 127.8_ = 41.98, *p* < 0.0001 | | | |
| **Mixed-effects model** | **Males: Naive-control vs Surgery**  **Time:** *F*_3.235, 112.7_ = 38.12, *p* < 0.0001  **Time x analgesic regimen:** *F*_24, 209_ = 3.521, *p* < 0.0001 | | | |
| Bonferroni *post hoc* test | Day 0_pre-OP | Surgery + NL | Surgery + NO | 0.0449 |
|  | Day 0_6h-post-OP | Surgery + NL | Surgery + NLO | 0.0381 |
|  | Day 1 | Naive-control | Surgery + NL | 0.0037 |
|  | Day 1 | Naive-control | Surgery + NLO | 0.0013 |
| **Mixed-effects model** | **Females: Naive-control vs Drug-control**  **Time:** *F*_3.939, 137.2_ = 37.7, *p* < 0.0001  **Time x analgesic regimen:** *F*_24,_ 209 = 2.253, *p* = 0.0012 | | | |
| **two-way RM ANOVA** | **Females: Naive-control vs Surgery**  **Time:** *F*_3.530, 123.5_ = 66.49, *p* < 0.0001  **Time x analgesic regimen:** *F*_24, 210_ = 3.778, *p* < 0.0001 | | | |
| Bonferroni *post hoc* test | Day 1 | Naive-control | Surgery + NO | 0.0004 |
|  | Day 1 | Naive-control | Surgery + NLO | 0.0088 |
|  | Day 2 | Naive-control | Surgery + NO | 0.0354 |
| **Body weight [g] (see Fig. S5a)** | | | | |
| **two-way RM ANOVA** | **Males: Naive-control vs Drug-control**  **Time:** *F*_1.162, 40.66_ = 16.29, *p* = 0.0001 | | | |
| **Mixed-effects model** | **Males: Naive-control vs Surgery**  **Time:** *F*_1.095, 38.2_ = 21.59, *p* < 0.0001  **Time x analgesic regimen:** *F*_36, 314_ = 1.988, *p* = 0.001 | | | |
| **Mixed-effects model** | **Females: Naive-control vs Drug-control**  **Time:** *F*_1.644, 57_ = 39.72, *p* < 0.0001 | | | |
| **Mixed-effects model** | **Females: Naive-control vs Surgery**  **Time:** *F*_1.498, 52.26_ = 44.95, *p* < 0.0001  **Time x analgesic regimen:** *F*_36,_ 314 = 1.664, *p* = 0.0122 | | | |

**Supplementary table 8: Statistical analyses of FCMs**

| **Statistical test** | **Time point** | **Experimental group** | **Experimental group** | ***p*-value** |
| --- | --- | --- | --- | --- |
| **one-way ANOVA** | **Males: Naive-control vs Surgery**  **Analgesic regimen:** *F*_4, 35_ = 3.019, *p* = 0.0307 | | | |
| Bonferroni | Day 1 | Naive-control | Surgery + NO | 0.0284 |
| **one-way ANOVA** | **Females: Naive-control vs Drug-control**  **Analgesic regimen:** *F*_4, 35_ = 3.399, *p* = 0.0189 | | | |
| Bonferroni | Day 1 | Drug-control + NL | Drug-control + NLO | 0.0189 |
| **one-way ANOVA** | **Females: Naive-control vs Surgery**  **Analgesic regimen:** *F*_4, 35_ = 5.253, *p* = 0.002 | | | |
| Bonferroni *post hoc* test | Day 1 | Naive-control | Surgery + NO | 0.0105 |
|  | Day 1 | Naive-control | Surgery + NLO | 0.0045 |

ANOVA = analysis of variance, RM = repeated measures, vs = versus, see Fig. 4b.

**Supplementary table 9: Numbers of animals reaching individual scores of Neuro score parameters**

| **Sex** | | **Males** | | | | | | | | | **Females** | | | | | | | | |
| --- | --- | --- | --- | --- | --- | --- | --- | --- | --- | --- | --- | --- | --- | --- | --- | --- | --- | --- | --- |
| **Experimental group** | | **NC.** | **Drug-control** | | | | **Surgery** | | | | **NC.** | **Drug-control** | | | | **Surgery** | | | |
| **Analgesic regimen** | | **no** | **N** | **NL** | **NO** | **NLO** | **N** | **NL** | **NO** | **NLO** | **no** | **N** | **NL** | **NO** | **NLO** | **N** | **NL** | **NO** | **NLO** |
| **Baseline** | | | | | | | | | | | | | | | | | | | |
| **Body position** | **-2** | 0 | 0 | 0 | 0 | 0 | 0 | 0 | 0 | 0 | 0 | 0 | 0 | 0 | 0 | 0 | 0 | 0 | 0 |
|  | **-1** | 0 | 0 | 0 | 0 | 0 | 0 | 0 | 0 | 0 | 0 | 0 | 0 | 0 | 0 | 0 | 0 | 0 | 0 |
|  | **0** | 8 | 8 | 8 | 8 | 8 | 8 | 8 | 8 | 8 | 8 | 8 | 8 | 8 | 8 | 8 | 8 | 8 | 8 |
|  | **1** | 0 | 0 | 0 | 0 | 0 | 0 | 0 | 0 | 0 | 0 | 0 | 0 | 0 | 0 | 0 | 0 | 0 | 0 |
|  | **2** | 0 | 0 | 0 | 0 | 0 | 0 | 0 | 0 | 0 | 0 | 0 | 0 | 0 | 0 | 0 | 0 | 0 | 0 |
| **Pelvic elevation** | **-1** | 0 | 0 | 0 | 0 | 0 | 0 | 0 | 0 | 0 | 0 | 0 | 0 | 0 | 0 | 0 | 0 | 0 | 0 |
|  | **0** | 8 | 7 | 5 | 6 | 8 | 7 | 8 | 7 | 7 | 7 | 7 | 8 | 8 | 8 | 8 | 7 | 7 | 8 |
|  | **1** | 0 | 1 | 3 | 2 | 0 | 1 | 0 | 1 | 1 | 1 | 1 | 0 | 0 | 0 | 0 | 1 | 1 | 0 |
| **Tail elevation** | **-1** | 0 | 0 | 0 | 0 | 0 | 0 | 0 | 0 | 0 | 0 | 0 | 0 | 0 | 0 | 0 | 0 | 0 | 0 |
|  | **0** | 8 | 8 | 8 | 8 | 8 | 8 | 8 | 8 | 8 | 8 | 8 | 8 | 8 | 8 | 8 | 8 | 8 | 8 |
|  | **1** | 0 | 0 | 0 | 0 | 0 | 0 | 0 | 0 | 0 | 0 | 0 | 0 | 0 | 0 | 0 | 0 | 0 | 0 |
|  | **2** | 0 | 0 | 0 | 0 | 0 | 0 | 0 | 0 | 0 | 0 | 0 | 0 | 0 | 0 | 0 | 0 | 0 | 0 |
| **Limb rotation** | **0** | 8 | 8 | 8 | 8 | 8 | 8 | 8 | 8 | 8 | 8 | 8 | 7 | 8 | 8 | 8 | 7 | 8 | 8 |
|  | **1** | 0 | 0 | 0 | 0 | 0 | 0 | 0 | 0 | 0 | 0 | 0 | 1 | 0 | 0 | 0 | 1 | 0 | 0 |
|  | **2** | 0 | 0 | 0 | 0 | 0 | 0 | 0 | 0 | 0 | 0 | 0 | 0 | 0 | 0 | 0 | 0 | 0 | 0 |
| **Locomotor activity** | **-2** | 0 | 0 | 0 | 0 | 0 | 0 | 0 | 0 | 0 | 0 | 0 | 0 | 0 | 0 | 0 | 0 | 0 | 0 |
|  | **-1** | 0 | 0 | 0 | 0 | 0 | 0 | 0 | 0 | 0 | 0 | 0 | 0 | 0 | 0 | 0 | 0 | 0 | 0 |
|  | **0** | 8 | 8 | 8 | 8 | 8 | 7 | 8 | 8 | 7 | 8 | 8 | 8 | 8 | 8 | 8 | 8 | 8 | 8 |
|  | **1** | 0 | 0 | 0 | 0 | 0 | 1 | 0 | 0 | 1 | 0 | 0 | 0 | 0 | 0 | 0 | 0 | 0 | 0 |
|  | **2** | 0 | 0 | 0 | 0 | 0 | 0 | 0 | 0 | 0 | 0 | 0 | 0 | 0 | 0 | 0 | 0 | 0 | 0 |
| **Respiratory rate** | **-2** | 0 | 0 | 0 | 0 | 0 | 0 | 0 | 0 | 0 | 0 | 0 | 0 | 0 | 0 | 0 | 0 | 0 | 0 |
|  | **-1** | 0 | 0 | 0 | 0 | 0 | 0 | 0 | 0 | 0 | 0 | 0 | 0 | 0 | 0 | 0 | 0 | 0 | 0 |
|  | **0** | 8 | 8 | 8 | 8 | 8 | 8 | 7 | 7 | 8 | 8 | 8 | 8 | 8 | 8 | 8 | 8 | 8 | 8 |
|  | **1** | 0 | 0 | 0 | 0 | 0 | 0 | 1 | 1 | 0 | 0 | 0 | 0 | 0 | 0 | 0 | 0 | 0 | 0 |
|  | **2** | 0 | 0 | 0 | 0 | 0 | 0 | 0 | 0 | 0 | 0 | 0 | 0 | 0 | 0 | 0 | 0 | 0 | 0 |
| **Piloerection** | **0** | 8 | 8 | 8 | 8 | 8 | 8 | 8 | 8 | 8 | 8 | 8 | 8 | 8 | 8 | 8 | 8 | 8 | 8 |
|  | **2** | 0 | 0 | 0 | 0 | 0 | 0 | 0 | 0 | 0 | 0 | 0 | 0 | 0 | 0 | 0 | 0 | 0 | 0 |
| **Ataxia** | **0** | 8 | 8 | 8 | 8 | 8 | 8 | 8 | 8 | 8 | 8 | 8 | 8 | 8 | 8 | 8 | 8 | 8 | 8 |
|  | **1** | 0 | 0 | 0 | 0 | 0 | 0 | 0 | 0 | 0 | 0 | 0 | 0 | 0 | 0 | 0 | 0 | 0 | 0 |
|  | **2** | 0 | 0 | 0 | 0 | 0 | 0 | 0 | 0 | 0 | 0 | 0 | 0 | 0 | 0 | 0 | 0 | 0 | 0 |
| **Freezing** | **0** | 8 | 8 | 8 | 8 | 8 | 8 | 8 | 8 | 8 | 8 | 8 | 8 | 8 | 8 | 8 | 8 | 8 | 8 |
|  | **1** | 0 | 0 | 0 | 0 | 0 | 0 | 0 | 0 | 0 | 0 | 0 | 0 | 0 | 0 | 0 | 0 | 0 | 0 |
|  | **2** | 0 | 0 | 0 | 0 | 0 | 0 | 0 | 0 | 0 | 0 | 0 | 0 | 0 | 0 | 0 | 0 | 0 | 0 |
|  | **2** | 0 | 0 | 0 | 0 | 0 | 0 | 0 | 0 |  | 0 | 0 | 0 | 0 | 0 | 0 | 0 | 0 | 0 |
| **Vocalisation Handling** | **0** | 4 | 2 | 3 | 3 | 1 | 3 | 1 | 5 | 1 | 1 | 0 | 7 | 0 | 2 | 1 | 0 | 0 | 0 |
|  | **1** | 4 | 6 | 5 | 5 | 7 | 5 | 7 | 3 | 7 | 7 | 8 | 1 | 8 | 6 | 7 | 8 | 8 | 8 |
| **Curiosity** | **-2** | 0 | 0 | 0 | 0 | 0 | 0 | 0 | 0 | 0 | 0 | 0 | 0 | 0 | 0 | 0 | 0 | 0 | 0 |
|  | **-1** | 0 | 0 | 0 | 0 | 0 | 0 | 0 | 0 | 0 | 0 | 0 | 0 | 0 | 0 | 0 | 0 | 0 | 0 |
|  | **0** | 7 | 8 | 8 | 8 | 7 | 8 | 8 | 8 | 8 | 8 | 8 | 8 | 8 | 8 | 8 | 8 | 8 | 8 |
|  | **1** | 1 | 0 | 0 | 0 | 1 | 0 | 0 | 0 | 0 | 0 | 0 | 0 | 0 | 0 | 0 | 0 | 0 | 0 |
|  | **2** | 0 | 0 | 0 | 0 | 0 | 0 | 0 | 0 | 0 | 0 | 0 | 0 | 0 | 0 | 0 | 0 | 0 | 0 |
| **Startle** | **-2** | 0 | 0 | 0 | 0 | 0 | 0 | 0 | 0 | 0 | 0 | 0 | 0 | 0 | 0 | 0 | 0 | 0 | 0 |
|  | **-1** | 0 | 0 | 0 | 0 | 0 | 0 | 0 | 0 | 0 | 0 | 0 | 0 | 0 | 0 | 0 | 0 | 0 | 0 |
|  | **0** | 7 | 8 | 8 | 8 | 8 | 8 | 8 | 8 | 8 | 8 | 8 | 8 | 8 | 8 | 8 | 8 | 8 | 8 |
|  | **1** | 1 | 0 | 0 | 0 | 0 | 0 | 0 | 0 | 0 | 0 | 0 | 0 | 0 | 0 | 0 | 0 | 0 | 0 |
|  | **2** | 0 | 0 | 0 | 0 | 0 | 0 | 0 | 0 | 0 | 0 | 0 | 0 | 0 | 0 | 0 | 0 | 0 | 0 |
| **Touch reaction** | **-2** | 0 | 0 | 0 | 0 | 0 | 0 | 0 | 0 | 0 | 0 | 0 | 0 | 0 | 0 | 0 | 0 | 0 | 0 |
|  | **-1** | 0 | 0 | 0 | 0 | 0 | 0 | 0 | 0 | 0 | 0 | 0 | 0 | 0 | 0 | 0 | 0 | 1 | 0 |
|  | **0** | 3 | 3 | 0 | 6 | 5 | 2 | 3 | 3 | 4 | 2 | 5 | 3 | 2 | 4 | 3 | 1 | 4 | 0 |
|  | **1** | 5 | 4 | 8 | 2 | 2 | 6 | 5 | 5 | 3 | 6 | 3 | 1 | 5 | 3 | 5 | 7 | 2 | 8 |
|  | **2** | 0 | 0 | 0 | 0 | 1 | 0 | 0 | 0 | 1 | 0 | 0 | 4 | 1 | 1 | 0 | 0 | 1 | 0 |
| **Irritability** | **0** | 8 | 8 | 7 | 7 | 8 | 7 | 8 | 7 | 8 | 6 | 6 | 8 | 7 | 7 | 7 | 5 | 8 | 8 |
|  | **1** | 0 | 0 | 1 | 1 | 0 | 1 | 0 | 1 | 0 | 2 | 2 | 0 | 1 | 1 | 1 | 3 | 0 | 0 |
|  | **2** | 0 | 0 | 0 | 0 | 0 | 0 | 0 | 0 | 0 | 0 | 0 | 0 | 0 | 0 | 0 | 0 | 0 | 0 |
| **Body tone** | **-2** | 0 | 0 | 0 | 0 | 0 | 0 | 0 | 0 | 0 | 0 | 0 | 0 | 0 | 0 | 0 | 0 | 0 | 0 |
|  | **-1** | 0 | 0 | 0 | 0 | 0 | 0 | 0 | 0 | 0 | 0 | 0 | 0 | 0 | 0 | 0 | 0 | 0 | 0 |
|  | **0** | 8 | 8 | 8 | 8 | 8 | 8 | 8 | 8 | 8 | 8 | 8 | 8 | 8 | 8 | 8 | 8 | 8 | 8 |
|  | **1** | 0 | 0 | 0 | 0 | 0 | 0 | 0 | 0 | 0 | 0 | 0 | 0 | 0 | 0 | 0 | 0 | 0 | 0 |
|  | **2** | 0 | 0 | 0 | 0 | 0 | 0 | 0 | 0 | 0 | 0 | 0 | 0 | 0 | 0 | 0 | 0 | 0 | 0 |
| **Abdominal tone** | **-1** | 0 | 0 | 0 | 0 | 0 | 1 | 0 | 0 | 0 | 0 | 0 | 0 | 0 | 0 | 0 | 0 | 0 | 0 |
|  | **0** | 8 | 8 | 8 | 7 | 8 | 7 | 8 | 8 | 8 | 6 | 8 | 5 | 7 | 6 | 6 | 7 | 8 | 6 |
|  | **1** | 0 | 0 | 0 | 1 | 0 | 0 | 0 | 0 | 0 | 2 | 0 | 3 | 1 | 2 | 2 | 1 | 0 | 2 |
| **Urination** | **0** | 4 | 3 | 4 | 3 | 3 | 1 | 1 | 2 | 0 | 3 | 6 | 1 | 2 | 3 | 4 | 2 | 0 | 2 |
|  | **1** | 4 | 5 | 4 | 5 | 5 | 7 | 7 | 6 | 8 | 5 | 2 | 7 | 6 | 5 | 4 | 6 | 8 | 6 |
| **Defecation** | **0** | 6 | 6 | 3 | 2 | 5 | 6 | 4 | 7 | 6 | 6 | 4 | 3 | 7 | 6 | 6 | 5 | 2 | 3 |
|  | **1** | 2 | 2 | 5 | 6 | 3 | 2 | 4 | 1 | 2 | 2 | 4 | 5 | 1 | 2 | 2 | 3 | 6 | 5 |
| **Sex** | | **Males** | | | | | | | | | **Females** | | | | | | | | |
| **Experimental group** | | **NC.** | **Drug-control** | | | | **Surgery** | | | | **NC.** | **Drug-control** | | | | **Surgery** | | | |
| **Analgesic regimen** | | **no** | **N** | **NL** | **NO** | **NLO** | **N** | **NL** | **NO** | **NLO** | **no** | **N** | **NL** | **NO** | **NLO** | **N** | **NL** | **NO** | **NLO** |
| **4 h post OP** | | | | | | | | | | | | | | | | | | | |
| **Body position** | **-2** | 0 | 0 | 0 | 0 | 0 | 0 | 0 | 0 | 0 | 0 | 0 | 0 | 0 | 0 | 0 | 0 | 0 | 0 |
|  | **-1** | 0 | 0 | 0 | 0 | 0 | 0 | 0 | 0 | 0 | 0 | 0 | 0 | 0 | 0 | 0 | 0 | 0 | 0 |
|  | **0** | 8 | 8 | 8 | 8 | 7 | 7 | 6 | 8 | 5 | 8 | 8 | 8 | 8 | 8 | 8 | 8 | 7 | 8 |
|  | **1** | 0 | 0 | 0 | 0 | 1 | 1 | 1 | 0 | 3 | 0 | 0 | 0 | 0 | 0 | 0 | 0 | 1 | 0 |
|  | **2** | 0 | 0 | 0 | 0 | 0 | 0 | 0 | 0 | 0 | 0 | 0 | 0 | 0 | 0 | 0 | 0 | 0 | 0 |
| **Pelvic elevation** | **-1** | 0 | 0 | 0 | 0 | 0 | 0 | 1 | 0 | 1 | 0 | 0 | 0 | 0 | 0 | 0 | 0 | 0 | 0 |
|  | **0** | 8 | 7 | 8 | 8 | 8 | 8 | 4 | 6 | 1 | 7 | 7 | 8 | 8 | 7 | 6 | 4 | 3 | 2 |
|  | **1** | 0 | 1 | 0 | 0 | 0 | 0 | 3 | 2 | 6 | 1 | 1 | 0 | 0 | 1 | 2 | 4 | 5 | 6 |
| **Tail elevation** | **-1** | 0 | 0 | 0 | 0 | 0 | 0 | 0 | 1 | 0 | 0 | 0 | 0 | 0 | 0 | 0 | 0 | 0 | 0 |
|  | **0** | 8 | 8 | 8 | 0 | 1 | 7 | 8 | 1 | 1 | 8 | 8 | 8 | 1 | 6 | 8 | 8 | 1 | 1 |
|  | **1** | 0 | 0 | 0 | 8 | 7 | 1 | 0 | 6 | 7 | 0 | 0 | 0 | 7 | 2 | 0 | 0 | 7 | 7 |
|  | **2** | 0 | 0 | 0 | 0 | 0 | 0 | 0 | 0 | 0 | 0 | 0 | 0 | 0 | 0 | 0 | 0 | 0 | 0 |
| **Limb rotation** | **0** | 8 | 6 | 8 | 8 | 8 | 8 | 6 | 8 | 8 | 8 | 7 | 7 | 8 | 8 | 5 | 8 | 7 | 6 |
|  | **1** | 0 | 2 | 0 | 0 | 0 | 0 | 2 | 0 | 0 | 0 | 1 | 1 | 0 | 0 | 3 | 0 | 1 | 2 |
|  | **2** | 0 | 0 | 0 | 0 | 0 | 0 | 0 | 0 | 0 | 0 | 0 | 0 | 0 | 0 | 0 | 0 | 0 | 0 |
| **Locomotor activity** | **-2** | 0 | 0 | 0 | 0 | 0 | 0 | 0 | 0 | 0 | 0 | 0 | 0 | 0 | 0 | 0 | 0 | 0 | 0 |
|  | **-1** | 0 | 0 | 0 | 0 | 0 | 0 | 0 | 0 | 0 | 0 | 0 | 0 | 0 | 0 | 3 | 2 | 1 | 0 |
|  | **0** | 8 | 8 | 8 | 4 | 6 | 7 | 8 | 6 | 7 | 8 | 7 | 8 | 5 | 3 | 5 | 6 | 6 | 7 |
|  | **1** | 0 | 0 | 0 | 4 | 2 | 0 | 0 | 2 | 1 | 0 | 1 | 0 | 3 | 5 | 0 | 0 | 1 | 1 |
|  | **2** | 0 | 0 | 0 | 0 | 0 | 1 | 0 | 0 | 0 | 0 | 0 | 0 | 0 | 0 | 0 | 0 | 0 | 0 |
| **Respiratory rate** | **-2** | 0 | 0 | 0 | 0 | 0 | 0 | 0 | 0 | 0 | 0 | 0 | 0 | 0 | 0 | 0 | 0 | 0 | 0 |
|  | **-1** | 0 | 0 | 0 | 0 | 0 | 0 | 0 | 0 | 0 | 0 | 0 | 0 | 0 | 0 | 0 | 0 | 0 | 0 |
|  | **0** | 8 | 8 | 8 | 8 | 8 | 8 | 7 | 8 | 8 | 8 | 8 | 8 | 8 | 8 | 8 | 8 | 8 | 8 |
|  | **1** | 0 | 0 | 0 | 0 | 0 | 0 | 1 | 0 | 0 | 0 | 0 | 0 | 0 | 0 | 0 | 0 | 0 | 0 |
|  | **2** | 0 | 0 | 0 | 0 | 0 | 0 | 0 | 0 | 0 | 0 | 0 | 0 | 0 | 0 | 0 | 0 | 0 | 0 |
| **Piloerection** | **0** | 8 | 8 | 8 | 8 | 8 | 8 | 8 | 8 | 8 | 8 | 8 | 8 | 8 | 8 | 7 | 8 | 8 | 8 |
|  | **2** | 0 | 0 | 0 | 0 | 0 | 0 | 0 | 0 | 0 | 0 | 0 | 0 | 0 | 0 | 1 | 0 | 0 | 0 |
| **Ataxia** | **0** | 8 | 8 | 8 | 8 | 8 | 7 | 8 | 8 | 8 | 8 | 8 | 8 | 8 | 8 | 8 | 8 | 8 | 8 |
|  | **1** | 0 | 0 | 0 | 0 | 0 | 1 | 0 | 0 | 0 | 0 | 0 | 0 | 0 | 0 | 0 | 0 | 0 | 0 |
|  | **2** | 0 | 0 | 0 | 0 | 0 | 0 | 0 | 0 | 0 | 0 | 0 | 0 | 0 | 0 | 0 | 0 | 0 | 0 |
| **Freezing** | **0** | 7 | 8 | 8 | 8 | 8 | 8 | 8 | 8 | 8 | 8 | 8 | 8 | 8 | 8 | 8 | 8 | 8 | 8 |
|  | **1** | 1 | 0 | 0 | 0 | 0 | 0 | 0 | 0 | 0 | 0 | 0 | 0 | 0 | 0 | 0 | 0 | 0 | 0 |
|  | **2** | 0 | 0 | 0 | 0 | 0 | 0 | 0 | 0 | 0 | 0 | 0 | 0 | 0 | 0 | 0 | 0 | 0 | 0 |
| **Vocalisation Handling** | **0** | 4 | 4 | 3 | 5 | 4 | 4 | 6 | 7 | 4 | 2 | 5 | 4 | 3 | 2 | 5 | 6 | 5 | 4 |
|  | **1** | 4 | 4 | 5 | 3 | 4 | 4 | 2 | 1 | 4 | 6 | 3 | 4 | 5 | 6 | 3 | 2 | 3 | 4 |
| **Curiosity** | **-2** | 0 | 0 | 0 | 1 | 1 | 1 | 0 | 1 | 1 | 0 | 1 | 2 | 0 | 1 | 0 | 2 | 0 | 0 |
|  | **-1** | 2 | 0 | 0 | 3 | 4 | 0 | 0 | 3 | 1 | 0 | 1 | 1 | 4 | 6 | 2 | 1 | 4 | 3 |
|  | **0** | 6 | 8 | 8 | 4 | 3 | 7 | 8 | 4 | 6 | 8 | 6 | 5 | 4 | 1 | 6 | 5 | 4 | 5 |
|  | **1** | 0 | 0 | 0 | 0 | 1 | 0 | 0 | 0 | 0 | 0 | 0 | 0 | 0 | 0 | 0 | 0 | 0 | 0 |
|  | **2** | 0 | 0 | 0 | 0 | 0 | 0 | 0 | 0 | 0 | 0 | 0 | 0 | 0 | 0 | 0 | 0 | 0 | 0 |
| **Startle** | **-2** | 0 | 0 | 0 | 0 | 0 | 0 | 0 | 0 | 0 | 0 | 0 | 0 | 0 | 0 | 0 | 0 | 0 | 0 |
|  | **-1** | 0 | 0 | 0 | 0 | 0 | 0 | 0 | 0 | 0 | 0 | 0 | 0 | 0 | 0 | 0 | 0 | 0 | 0 |
|  | **0** | 7 | 8 | 7 | 7 | 7 | 8 | 8 | 8 | 8 | 8 | 8 | 8 | 8 | 8 | 8 | 8 | 8 | 8 |
|  | **1** | 1 | 0 | 1 | 1 | 1 | 0 | 0 | 0 | 0 | 0 | 0 | 0 | 0 | 0 | 0 | 0 | 0 | 0 |
|  | **2** | 0 | 0 | 0 | 0 | 0 | 0 | 0 | 0 | 0 | 0 | 0 | 0 | 0 | 0 | 0 | 0 | 0 | 0 |
| **Touch reaction** | **-2** | 0 | 0 | 0 | 0 | 0 | 1 | 0 | 0 | 0 | 0 | 0 | 0 | 0 | 0 | 0 | 0 | 0 | 0 |
|  | **-1** | 0 | 0 | 0 | 0 | 1 | 0 | 1 | 1 | 0 | 0 | 0 | 0 | 0 | 0 | 1 | 2 | 0 | 0 |
|  | **0** | 3 | 3 | 1 | 0 | 0 | 3 | 5 | 4 | 5 | 0 | 4 | 1 | 2 | 0 | 5 | 4 | 3 | 3 |
|  | **1** | 4 | 4 | 7 | 7 | 5 | 3 | 2 | 3 | 3 | 5 | 3 | 5 | 3 | 3 | 1 | 1 | 5 | 4 |
|  | **2** | 1 | 1 | 0 | 1 | 2 | 1 | 0 | 0 | 0 | 3 | 1 | 2 | 3 | 5 | 1 | 1 | 0 | 1 |
| **Irritability** | **0** | 7 | 7 | 5 | 7 | 7 | 5 | 7 | 6 | 6 | 6 | 1 | 4 | 5 | 7 | 5 | 5 | 6 | 8 |
|  | **1** | 1 | 1 | 3 | 1 | 1 | 3 | 1 | 2 | 2 | 0 | 5 | 2 | 2 | 1 | 2 | 2 | 2 | 0 |
|  | **2** | 0 | 0 | 0 | 0 | 0 | 0 | 0 | 0 | 0 | 2 | 2 | 2 | 1 | 0 | 1 | 1 | 0 | 0 |
| **Body tone** | **-2** | 0 | 0 | 0 | 0 | 0 | 0 | 0 | 0 | 0 | 0 | 0 | 0 | 0 | 0 | 0 | 0 | 0 | 0 |
|  | **-1** | 0 | 1 | 0 | 0 | 0 | 0 | 1 | 0 | 0 | 0 | 0 | 0 | 0 | 0 | 0 | 0 | 0 | 0 |
|  | **0** | 8 | 7 | 6 | 8 | 8 | 8 | 6 | 7 | 8 | 8 | 6 | 6 | 8 | 8 | 8 | 8 | 7 | 8 |
|  | **1** | 0 | 0 | 2 | 0 | 0 | 0 | 1 | 1 | 0 | 0 | 2 | 2 | 0 | 0 | 0 | 0 | 1 | 0 |
|  | **2** | 0 | 0 | 0 | 0 | 0 | 0 | 0 | 0 | 0 | 0 | 0 | 0 | 0 | 0 | 0 | 0 | 0 | 0 |
| **Abdominal tone** | **-1** | 0 | 2 | 0 | 0 | 0 | 0 | 0 | 0 | 0 | 0 | 0 | 0 | 0 | 0 | 0 | 0 | 0 | 0 |
|  | **0** | 8 | 6 | 8 | 8 | 7 | 8 | 8 | 7 | 6 | 7 | 8 | 7 | 8 | 8 | 7 | 8 | 8 | 7 |
|  | **1** | 0 | 0 | 0 | 0 | 1 | 0 | 0 | 1 | 2 | 1 | 0 | 1 | 0 | 0 | 1 | 0 | 0 | 1 |
| **Urination** | **0** | 0 | 6 | 4 | 3 | 5 | 7 | 3 | 6 | 6 | 1 | 3 | 0 | 5 | 5 | 5 | 3 | 4 | 2 |
|  | **1** | 8 | 2 | 4 | 5 | 3 | 1 | 5 | 2 | 2 | 7 | 5 | 8 | 3 | 3 | 3 | 5 | 4 | 6 |
| **Defecation** | **0** | 4 | 6 | 3 | 2 | 6 | 6 | 6 | 7 | 7 | 2 | 4 | 1 | 5 | 3 | 5 | 6 | 6 | 7 |
|  | **1** | 4 | 2 | 5 | 6 | 2 | 2 | 2 | 1 | 1 | 6 | 4 | 7 | 3 | 5 | 3 | 2 | 2 | 1 |
| **Sex** | | **Males** | | | | | | | | | **Females** | | | | | | | | |
| **Experimental group** | | **NC.** | **Drug-control** | | | | **Surgery** | | | | **NC.** | **Drug-control** | | | | **Surgery** | | | |
| **Analgesic regimen** | | **no** | **N** | **NL** | **NO** | **NLO** | **N** | **NL** | **NO** | **NLO** | **no** | **N** | **NL** | **NO** | **NLO** | **N** | **NL** | **NO** | **NLO** |
| **Day 1** | | | | | | | | | | | | | | | | | | | |
| **Body position** | **-2** | 0 | 0 | 0 | 0 | 0 | 0 | 0 | 0 | 0 | 0 | 0 | 0 | 0 | 0 | 0 | 0 | 0 | 0 |
|  | **-1** | 0 | 0 | 0 | 0 | 0 | 0 | 0 | 0 | 0 | 0 | 0 | 0 | 0 | 0 | 0 | 0 | 0 | 0 |
|  | **0** | 8 | 8 | 8 | 8 | 8 | 8 | 8 | 8 | 8 | 8 | 8 | 8 | 8 | 8 | 8 | 8 | 8 | 8 |
|  | **1** | 0 | 0 | 0 | 0 | 0 | 0 | 0 | 0 | 0 | 0 | 0 | 0 | 0 | 0 | 0 | 0 | 0 | 0 |
|  | **2** | 0 | 0 | 0 | 0 | 0 | 0 | 0 | 0 | 0 | 0 | 0 | 0 | 0 | 0 | 0 | 0 | 0 | 0 |
| **Pelvic elevation** | **-1** | 0 | 0 | 0 | 0 | 0 | 0 | 0 | 0 | 0 | 0 | 0 | 0 | 0 | 0 | 0 | 0 | 0 | 0 |
|  | **0** | 8 | 8 | 8 | 8 | 8 | 7 | 5 | 4 | 3 | 7 | 7 | 8 | 8 | 8 | 7 | 5 | 4 | 5 |
|  | **1** | 0 | 0 | 0 | 0 | 0 | 1 | 3 | 4 | 5 | 1 | 1 | 0 | 0 | 0 | 1 | 3 | 4 | 3 |
| **Tail elevation** | **-1** | 0 | 0 | 0 | 0 | 0 | 0 | 0 | 0 | 0 | 0 | 0 | 0 | 0 | 0 | 0 | 0 | 0 | 0 |
|  | **0** | 8 | 8 | 8 | 8 | 8 | 8 | 8 | 8 | 8 | 8 | 8 | 8 | 8 | 8 | 8 | 8 | 8 | 8 |
|  | **1** | 0 | 0 | 0 | 0 | 0 | 0 | 0 | 0 | 0 | 0 | 0 | 0 | 0 | 0 | 0 | 0 | 0 | 0 |
|  | **2** | 0 | 0 | 0 | 0 | 0 | 0 | 0 | 0 | 0 | 0 | 0 | 0 | 0 | 0 | 0 | 0 | 0 | 0 |
| **Limb rotation** | **0** | 8 | 8 | 8 | 8 | 8 | 7 | 8 | 8 | 6 | 8 | 8 | 7 | 8 | 8 | 6 | 5 | 5 | 3 |
|  | **1** | 0 | 0 | 0 | 0 | 0 | 1 | 0 | 0 | 2 | 0 | 0 | 1 | 0 | 0 | 2 | 3 | 3 | 5 |
|  | **2** | 0 | 0 | 0 | 0 | 0 | 0 | 0 | 0 | 0 | 0 | 0 | 0 | 0 | 0 | 0 | 0 | 0 | 0 |
| **Locomotor activity** | **-2** | 0 | 0 | 0 | 0 | 0 | 0 | 0 | 0 | 0 | 0 | 0 | 0 | 0 | 0 | 0 | 0 | 0 | 0 |
|  | **-1** | 0 | 0 | 0 | 0 | 0 | 2 | 2 | 0 | 0 | 0 | 0 | 0 | 0 | 0 | 0 | 2 | 2 | 2 |
|  | **0** | 8 | 8 | 8 | 8 | 8 | 5 | 6 | 8 | 8 | 8 | 7 | 8 | 8 | 8 | 5 | 6 | 6 | 6 |
|  | **1** | 0 | 0 | 0 | 0 | 0 | 1 | 0 | 0 | 0 | 0 | 1 | 0 | 0 | 0 | 3 | 0 | 0 | 0 |
|  | **2** | 0 | 0 | 0 | 0 | 0 | 0 | 0 | 0 | 0 | 0 | 0 | 0 | 0 | 0 | 0 | 0 | 0 | 0 |
| **Respiratory rate** | **-2** | 0 | 0 | 0 | 0 | 0 | 0 | 0 | 0 | 0 | 0 | 0 | 0 | 0 | 0 | 0 | 0 | 0 | 0 |
|  | **-1** | 0 | 0 | 0 | 0 | 0 | 0 | 0 | 0 | 0 | 0 | 0 | 0 | 0 | 0 | 0 | 0 | 0 | 0 |
|  | **0** | 8 | 8 | 8 | 8 | 8 | 8 | 8 | 7 | 8 | 8 | 8 | 8 | 8 | 8 | 8 | 8 | 7 | 8 |
|  | **1** | 0 | 0 | 0 | 0 | 0 | 0 | 0 | 1 | 0 | 0 | 0 | 0 | 0 | 0 | 0 | 0 | 1 | 0 |
|  | **2** | 0 | 0 | 0 | 0 | 0 | 0 | 0 | 0 | 0 | 0 | 0 | 0 | 0 | 0 | 0 | 0 | 0 | 0 |
| **Piloerection** | **0** | 8 | 8 | 8 | 8 | 8 | 8 | 8 | 8 | 8 | 8 | 8 | 8 | 8 | 8 | 8 | 8 | 8 | 8 |
|  | **2** | 0 | 0 | 0 | 0 | 0 | 0 | 0 | 0 | 0 | 0 | 0 | 0 | 0 | 0 | 0 | 0 | 0 | 0 |
| **Ataxia** | **0** | 8 | 8 | 8 | 8 | 8 | 8 | 8 | 8 | 8 | 8 | 8 | 8 | 8 | 8 | 8 | 8 | 8 | 8 |
|  | **1** | 0 | 0 | 0 | 0 | 0 | 0 | 0 | 0 | 0 | 0 | 0 | 0 | 0 | 0 | 0 | 0 | 0 | 0 |
|  | **2** | 0 | 0 | 0 | 0 | 0 | 0 | 0 | 0 | 0 | 0 | 0 | 0 | 0 | 0 | 0 | 0 | 0 | 0 |
| **Freezing** | **0** | 8 | 8 | 8 | 8 | 8 | 8 | 8 | 8 | 8 | 8 | 8 | 8 | 8 | 8 | 8 | 8 | 8 | 8 |
|  | **1** | 0 | 0 | 0 | 0 | 0 | 0 | 0 | 0 | 0 | 0 | 0 | 0 | 0 | 0 | 0 | 0 | 0 | 0 |
|  | **2** | 0 | 0 | 0 | 0 | 0 | 0 | 0 | 0 | 0 | 0 | 0 | 0 | 0 | 0 | 0 | 0 | 0 | 0 |
| **Vocalisation Handling** | **0** | 2 | 4 | 2 | 7 | 0 | 0 | 1 | 1 | 1 | 0 | 0 | 0 | 0 | 1 | 1 | 3 | 0 | 2 |
|  | **1** | 6 | 4 | 6 | 1 | 8 | 8 | 7 | 7 | 7 | 8 | 8 | 8 | 8 | 7 | 7 | 5 | 8 | 6 |
| **Curiosity** | **-2** | 0 | 0 | 0 | 0 | 0 | 0 | 0 | 0 | 0 | 0 | 0 | 0 | 0 | 0 | 0 | 0 | 0 | 0 |
|  | **-1** | 0 | 0 | 0 | 1 | 1 | 1 | 0 | 0 | 0 | 1 | 0 | 0 | 0 | 0 | 0 | 0 | 0 | 1 |
|  | **0** | 8 | 8 | 8 | 7 | 7 | 7 | 8 | 8 | 8 | 7 | 8 | 8 | 8 | 8 | 8 | 8 | 8 | 7 |
|  | **1** | 0 | 0 | 0 | 0 | 0 | 0 | 0 | 0 | 0 | 0 | 0 | 0 | 0 | 0 | 0 | 0 | 0 | 0 |
|  | **2** | 0 | 0 | 0 | 0 | 0 | 0 | 0 | 0 | 0 | 0 | 0 | 0 | 0 | 0 | 0 | 0 | 0 | 0 |
| **Startle** | **-2** | 0 | 0 | 0 | 0 | 0 | 0 | 0 | 0 | 0 | 0 | 0 | 0 | 0 | 0 | 0 | 0 | 0 | 0 |
|  | **-1** | 0 | 0 | 0 | 0 | 0 | 0 | 0 | 0 | 0 | 0 | 0 | 0 | 0 | 0 | 0 | 0 | 0 | 0 |
|  | **0** | 7 | 8 | 8 | 8 | 8 | 8 | 8 | 8 | 8 | 8 | 8 | 8 | 8 | 8 | 8 | 8 | 8 | 8 |
|  | **1** | 1 | 0 | 0 | 0 | 0 | 0 | 0 | 0 | 0 | 0 | 0 | 0 | 0 | 0 | 0 | 0 | 0 | 0 |
|  | **2** | 0 | 0 | 0 | 0 | 0 | 0 | 0 | 0 | 0 | 0 | 0 | 0 | 0 | 0 | 0 | 0 | 0 | 0 |
| **Touch reaction** | **-2** | 0 | 0 | 0 | 0 | 0 | 0 | 0 | 0 | 0 | 0 | 0 | 0 | 0 | 0 | 0 | 0 | 0 | 0 |
|  | **-1** | 0 | 0 | 0 | 0 | 0 | 0 | 0 | 1 | 0 | 0 | 0 | 0 | 0 | 0 | 0 | 0 | 0 | 3 |
|  | **0** | 3 | 2 | 4 | 0 | 3 | 5 | 8 | 5 | 8 | 1 | 4 | 1 | 2 | 2 | 6 | 6 | 5 | 0 |
|  | **1** | 5 | 6 | 4 | 7 | 5 | 3 | 0 | 2 | 0 | 4 | 3 | 4 | 3 | 3 | 1 | 2 | 3 | 4 |
|  | **2** | 0 | 0 | 0 | 1 | 0 | 0 | 0 | 0 | 0 | 3 | 1 | 3 | 3 | 3 | 1 | 0 | 0 | 1 |
| **Irritability** | **0** | 7 | 8 | 6 | 7 | 8 | 8 | 8 | 8 | 8 | 7 | 6 | 8 | 8 | 6 | 8 | 7 | 8 | 8 |
|  | **1** | 1 | 0 | 2 | 1 | 0 | 0 | 0 | 0 | 0 | 1 | 1 | 0 | 0 | 2 | 0 | 1 | 0 | 0 |
|  | **2** | 0 | 0 | 0 | 0 | 0 | 0 | 0 | 0 | 0 | 0 | 1 | 0 | 0 | 0 | 0 | 0 | 0 | 0 |
| **Body tone** | **-2** | 0 | 0 | 0 | 0 | 0 | 0 | 0 | 0 | 0 | 0 | 0 | 0 | 0 | 0 | 0 | 0 | 0 | 0 |
|  | **-1** | 0 | 0 | 0 | 0 | 0 | 0 | 0 | 0 | 0 | 0 | 0 | 0 | 0 | 0 | 0 | 0 | 0 | 0 |
|  | **0** | 8 | 8 | 6 | 8 | 8 | 8 | 8 | 8 | 8 | 8 | 8 | 8 | 8 | 8 | 8 | 8 | 8 | 8 |
|  | **1** | 0 | 0 | 2 | 0 | 0 | 0 | 0 | 0 | 0 | 0 | 0 | 0 | 0 | 0 | 0 | 0 | 0 | 0 |
|  | **2** | 0 | 0 | 0 | 0 | 0 | 0 | 0 | 0 | 0 | 0 | 0 | 0 | 0 | 0 | 0 | 0 | 0 | 0 |
| **Abdominal tone** | **-1** | 0 | 0 | 0 | 0 | 0 | 0 | 0 | 0 | 0 | 0 | 0 | 0 | 0 | 0 | 0 | 0 | 0 | 0 |
|  | **0** | 8 | 8 | 8 | 8 | 8 | 8 | 8 | 8 | 8 | 8 | 8 | 8 | 8 | 8 | 7 | 8 | 8 | 8 |
|  | **1** | 0 | 0 | 0 | 0 | 0 | 0 | 0 | 0 | 0 | 0 | 0 | 0 | 0 | 0 | 1 | 0 | 0 | 0 |
| **Urination** | **0** | 1 | 5 | 5 | 3 | 4 | 5 | 3 | 5 | 6 | 4 | 1 | 3 | 3 | 5 | 6 | 3 | 2 | 4 |
|  | **1** | 7 | 3 | 3 | 5 | 4 | 3 | 5 | 3 | 2 | 4 | 7 | 5 | 5 | 3 | 2 | 5 | 6 | 4 |
| **Defecation** | **0** | 4 | 4 | 7 | 2 | 3 | 6 | 7 | 5 | 5 | 4 | 5 | 2 | 5 | 4 | 3 | 3 | 3 | 2 |
|  | **1** | 4 | 4 | 1 | 6 | 5 | 2 | 1 | 3 | 3 | 4 | 3 | 6 | 3 | 4 | 5 | 5 | 5 | 6 |
| **Sex** | | **Males** | | | | | | | | | **Females** | | | | | | | | |
| **Experimental group** | | **NC.** | **Drug-control** | | | | **Surgery** | | | | **NC.** | **Drug-control** | | | | **Surgery** | | | |
| **Analgesic regimen** | | **no** | **N** | **NL** | **NO** | **NLO** | **N** | **NL** | **NO** | **NLO** | **no** | **N** | **NL** | **NO** | **NLO** | **N** | **NL** | **NO** | **NLO** |
| **Day 2** | | | | | | | | | | | | | | | | | | | |
| **Body position** | **-2** | 0 | 0 | 0 | 0 | 0 | 0 | 0 | 0 | 0 | 0 | 0 | 0 | 0 | 0 | 0 | 0 | 0 | 0 |
|  | **-1** | 0 | 0 | 0 | 0 | 0 | 0 | 0 | 0 | 0 | 0 | 0 | 0 | 0 | 0 | 0 | 0 | 0 | 0 |
|  | **0** | 8 | 8 | 8 | 8 | 8 | 8 | 8 | 8 | 8 | 8 | 8 | 8 | 8 | 8 | 8 | 8 | 8 | 8 |
|  | **1** | 0 | 0 | 0 | 0 | 0 | 0 | 0 | 0 | 0 | 0 | 0 | 0 | 0 | 0 | 0 | 0 | 0 | 0 |
|  | **2** | 0 | 0 | 0 | 0 | 0 | 0 | 0 | 0 | 0 | 0 | 0 | 0 | 0 | 0 | 0 | 0 | 0 | 0 |
| **Pelvic elevation** | **-1** | 0 | 0 | 0 | 0 | 0 | 0 | 0 | 0 | 0 | 0 | 0 | 0 | 0 | 0 | 0 | 0 | 0 | 0 |
|  | **0** | 8 | 8 | 8 | 6 | 8 | 7 | 6 | 8 | 6 | 7 | 7 | 8 | 8 | 8 | 7 | 4 | 4 | 5 |
|  | **1** | 0 | 0 | 0 | 2 | 0 | 1 | 2 | 0 | 2 | 1 | 1 | 0 | 0 | 0 | 1 | 4 | 4 | 3 |
| **Tail elevation** | **-1** | 0 | 0 | 0 | 0 | 0 | 0 | 0 | 0 | 0 | 0 | 0 | 0 | 0 | 0 | 0 | 0 | 0 | 0 |
|  | **0** | 8 | 8 | 7 | 8 | 8 | 8 | 8 | 8 | 8 | 8 | 8 | 8 | 8 | 8 | 7 | 8 | 8 | 8 |
|  | **1** | 0 | 0 | 1 | 0 | 0 | 0 | 0 | 0 | 0 | 0 | 0 | 0 | 0 | 0 | 1 | 0 | 0 | 0 |
|  | **2** | 0 | 0 | 0 | 0 | 0 | 0 | 0 | 0 | 0 | 0 | 0 | 0 | 0 | 0 | 0 | 0 | 0 | 0 |
| **Limb rotation** | **0** | 8 | 8 | 8 | 8 | 8 | 7 | 8 | 8 | 7 | 8 | 7 | 7 | 8 | 8 | 5 | 5 | 4 | 3 |
|  | **1** | 0 | 0 | 0 | 0 | 0 | 1 | 0 | 0 | 1 | 0 | 1 | 1 | 0 | 0 | 3 | 3 | 4 | 5 |
|  | **2** | 0 | 0 | 0 | 0 | 0 | 0 | 0 | 0 | 0 | 0 | 0 | 0 | 0 | 0 | 0 | 0 | 0 | 0 |
| **Locomotor activity** | **-2** | 0 | 0 | 0 | 0 | 0 | 0 | 0 | 0 | 0 | 0 | 0 | 0 | 0 | 0 | 0 | 0 | 0 | 0 |
|  | **-1** | 0 | 0 | 0 | 0 | 0 | 2 | 1 | 0 | 0 | 0 | 0 | 0 | 0 | 0 | 0 | 1 | 1 | 0 |
|  | **0** | 8 | 8 | 8 | 8 | 8 | 6 | 7 | 8 | 7 | 8 | 8 | 8 | 8 | 8 | 8 | 7 | 7 | 8 |
|  | **1** | 0 | 0 | 0 | 0 | 0 | 0 | 0 | 0 | 1 | 0 | 0 | 0 | 0 | 0 | 0 | 0 | 0 | 0 |
|  | **2** | 0 | 0 | 0 | 0 | 0 | 0 | 0 | 0 | 0 | 0 | 0 | 0 | 0 | 0 | 0 | 0 | 0 | 0 |
| **Respiratory rate** | **-2** | 0 | 0 | 0 | 0 | 0 | 0 | 0 | 0 | 0 | 0 | 0 | 0 | 0 | 0 | 0 | 0 | 0 | 0 |
|  | **-1** | 0 | 0 | 0 | 0 | 0 | 0 | 0 | 0 | 0 | 0 | 0 | 0 | 0 | 0 | 0 | 0 | 0 | 0 |
|  | **0** | 8 | 8 | 8 | 8 | 8 | 8 | 8 | 8 | 8 | 8 | 8 | 8 | 8 | 8 | 8 | 8 | 8 | 8 |
|  | **1** | 0 | 0 | 0 | 0 | 0 | 0 | 0 | 0 | 0 | 0 | 0 | 0 | 0 | 0 | 0 | 0 | 0 | 0 |
|  | **2** | 0 | 0 | 0 | 0 | 0 | 0 | 0 | 0 | 0 | 0 | 0 | 0 | 0 | 0 | 0 | 0 | 0 | 0 |
| **Piloerection** | **0** | 8 | 8 | 8 | 8 | 8 | 8 | 8 | 8 | 8 | 8 | 8 | 8 | 8 | 8 | 8 | 8 | 8 | 8 |
|  | **2** | 0 | 0 | 0 | 0 | 0 | 0 | 0 | 0 | 0 | 0 | 0 | 0 | 0 | 0 | 0 | 0 | 0 | 0 |
| **Ataxia** | **0** | 8 | 8 | 8 | 8 | 8 | 8 | 8 | 8 | 8 | 8 | 8 | 8 | 8 | 8 | 8 | 8 | 8 | 8 |
|  | **1** | 0 | 0 | 0 | 0 | 0 | 0 | 0 | 0 | 0 | 0 | 0 | 0 | 0 | 0 | 0 | 0 | 0 | 0 |
|  | **2** | 0 | 0 | 0 | 0 | 0 | 0 | 0 | 0 | 0 | 0 | 0 | 0 | 0 | 0 | 0 | 0 | 0 | 0 |
| **Freezing** | **0** | 7 | 8 | 8 | 8 | 8 | 8 | 8 | 8 | 8 | 8 | 8 | 8 | 8 | 8 | 8 | 8 | 8 | 8 |
|  | **1** | 1 | 0 | 0 | 0 | 0 | 0 | 0 | 0 | 0 | 0 | 0 | 0 | 0 | 0 | 0 | 0 | 0 | 0 |
|  | **2** | 0 | 0 | 0 | 0 | 0 | 0 | 0 | 0 | 0 | 0 | 0 | 0 | 0 | 0 | 0 | 0 | 0 | 0 |
| **Vocalisation Handling** | 0 | 3 | 1 | 0 | 2 | 1 | 0 | 3 | 4 | 2 | 0 | 1 | 0 | 0 | 0 | 1 | 3 | 0 | 0 |
|  | **1** | 5 | 7 | 8 | 6 | 7 | 8 | 5 | 4 | 6 | 8 | 7 | 8 | 8 | 8 | 7 | 5 | 8 | 8 |
| **Curiosity** | **-2** | 0 | 0 | 0 | 0 | 0 | 0 | 0 | 0 | 0 | 0 | 0 | 0 | 0 | 0 | 0 | 0 | 0 | 0 |
|  | **-1** | 0 | 0 | 0 | 0 | 0 | 0 | 0 | 0 | 0 | 0 | 0 | 0 | 0 | 0 | 0 | 0 | 0 | 0 |
|  | **0** | 8 | 8 | 8 | 8 | 8 | 8 | 8 | 8 | 8 | 8 | 8 | 8 | 8 | 8 | 8 | 8 | 8 | 8 |
|  | **1** | 0 | 0 | 0 | 0 | 0 | 0 | 0 | 0 | 0 | 0 | 0 | 0 | 0 | 0 | 0 | 0 | 0 | 0 |
|  | **2** | 0 | 0 | 0 | 0 | 0 | 0 | 0 | 0 | 0 | 0 | 0 | 0 | 0 | 0 | 0 | 0 | 0 | 0 |
| **Startle** | **-2** | 0 | 0 | 0 | 0 | 0 | 0 | 0 | 0 | 0 | 0 | 0 | 0 | 0 | 0 | 0 | 0 | 0 | 0 |
|  | **-1** | 0 | 0 | 0 | 0 | 0 | 0 | 0 | 0 | 0 | 0 | 0 | 0 | 0 | 0 | 0 | 0 | 0 | 0 |
|  | **0** | 7 | 8 | 8 | 8 | 8 | 8 | 8 | 8 | 8 | 8 | 8 | 8 | 8 | 8 | 8 | 8 | 8 | 8 |
|  | **1** | 1 | 0 | 0 | 0 | 0 | 0 | 0 | 0 | 0 | 0 | 0 | 0 | 0 | 0 | 0 | 0 | 0 | 0 |
|  | **2** | 0 | 0 | 0 | 0 | 0 | 0 | 0 | 0 | 0 | 0 | 0 | 0 | 0 | 0 | 0 | 0 | 0 | 0 |
| **Touch reaction** | **-2** | 0 | 0 | 0 | 0 | 0 | 0 | 0 | 0 | 0 | 0 | 0 | 0 | 0 | 0 | 0 | 0 | 0 | 0 |
|  | **-1** | 0 | 0 | 0 | 1 | 0 | 2 | 1 | 0 | 1 | 0 | 0 | 0 | 0 | 0 | 0 | 0 | 0 | 0 |
|  | **0** | 4 | 1 | 1 | 1 | 4 | 3 | 4 | 7 | 6 | 0 | 5 | 1 | 1 | 0 | 4 | 4 | 4 | 2 |
|  | **1** | 3 | 7 | 7 | 5 | 4 | 3 | 3 | 1 | 1 | 5 | 2 | 3 | 5 | 5 | 4 | 2 | 3 | 6 |
|  | **2** | 1 | 0 | 0 | 1 | 0 | 0 | 0 | 0 | 0 | 3 | 1 | 4 | 2 | 3 | 0 | 2 | 1 | 0 |
| **Irritability** | **0** | 7 | 8 | 6 | 8 | 8 | 8 | 8 | 8 | 8 | 7 | 5 | 8 | 8 | 7 | 7 | 8 | 8 | 8 |
|  | **1** | 1 | 0 | 2 | 0 | 0 | 0 | 0 | 0 | 0 | 1 | 2 | 0 | 0 | 1 | 1 | 0 | 0 | 0 |
|  | **2** | 0 | 0 | 0 | 0 | 0 | 0 | 0 | 0 | 0 | 0 | 1 | 0 | 0 | 0 | 0 | 0 | 0 | 0 |
| **Body tone** | **-2** | 0 | 0 | 0 | 0 | 0 | 0 | 0 | 0 | 0 | 0 | 0 | 0 | 0 | 0 | 0 | 0 | 0 | 0 |
|  | **-1** | 0 | 0 | 0 | 0 | 0 | 0 | 0 | 0 | 0 | 0 | 0 | 0 | 0 | 0 | 0 | 0 | 0 | 0 |
|  | **0** | 8 | 8 | 6 | 8 | 8 | 8 | 8 | 8 | 8 | 8 | 8 | 8 | 8 | 8 | 8 | 8 | 8 | 8 |
|  | **1** | 0 | 0 | 2 | 0 | 0 | 0 | 0 | 0 | 0 | 0 | 0 | 0 | 0 | 0 | 0 | 0 | 0 | 0 |
|  | **2** | 0 | 0 | 0 | 0 | 0 | 0 | 0 | 0 | 0 | 0 | 0 | 0 | 0 | 0 | 0 | 0 | 0 | 0 |
| **Abdominal tone** | **-1** | 0 | 0 | 0 | 0 | 1 | 0 | 0 | 0 | 0 | 0 | 0 | 0 | 0 | 0 | 0 | 0 | 0 | 0 |
|  | **0** | 8 | 8 | 8 | 8 | 7 | 8 | 8 | 8 | 8 | 8 | 8 | 8 | 8 | 8 | 7 | 8 | 8 | 8 |
|  | **1** | 0 | 0 | 0 | 0 | 0 | 0 | 0 | 0 | 0 | 0 | 0 | 0 | 0 | 0 | 1 | 0 | 0 | 0 |
| **Urination** | **0** | 4 | 7 | 6 | 6 | 6 | 6 | 5 | 7 | 4 | 3 | 5 | 5 | 5 | 3 | 7 | 4 | 4 | 4 |
|  | **1** | 4 | 1 | 2 | 2 | 2 | 2 | 3 | 1 | 4 | 5 | 3 | 3 | 3 | 5 | 1 | 4 | 4 | 4 |
| **Defecation** | **0** | 3 | 3 | 6 | 5 | 2 | 4 | 4 | 4 | 6 | 5 | 6 | 5 | 3 | 3 | 6 | 3 | 4 | 4 |
|  | **1** | 5 | 5 | 2 | 3 | 6 | 4 | 4 | 4 | 2 | 3 | 2 | 3 | 5 | 5 | 2 | 5 | 4 | 4 |
| **Sex** | | **Males** | | | | | | | | | **Females** | | | | | | | | |
| **Experimental group** | | **NC.** | **Drug-control** | | | | **Surgery** | | | | **NC.** | **Drug-control** | | | | **Surgery** | | | |
| **Analgesic regimen** | | **no** | **N** | **NL** | **NO** | **NLO** | **N** | **NL** | **NO** | **NLO** | **no** | **N** | **NL** | **NO** | **NLO** | **N** | **NL** | **NO** | **NLO** |
| **Day 3** | | | | | | | | | | | | | | | | | | | |
| **Body position** | **-2** | 0 | 0 | 0 | 0 | 0 | 0 | 0 | 0 | 0 | 0 | 0 | 0 | 0 | 0 | 0 | 0 | 0 | 0 |
|  | **-1** | 0 | 0 | 0 | 0 | 0 | 0 | 0 | 0 | 0 | 0 | 0 | 0 | 0 | 0 | 0 | 0 | 0 | 0 |
|  | **0** | 8 | 8 | 8 | 8 | 8 | 8 | 8 | 8 | 8 | 8 | 8 | 8 | 8 | 8 | 8 | 8 | 8 | 8 |
|  | **1** | 0 | 0 | 0 | 0 | 0 | 0 | 0 | 0 | 0 | 0 | 0 | 0 | 0 | 0 | 0 | 0 | 0 | 0 |
|  | **2** | 0 | 0 | 0 | 0 | 0 | 0 | 0 | 0 | 0 | 0 | 0 | 0 | 0 | 0 | 0 | 0 | 0 | 0 |
| **Pelvic elevation** | **-1** | 0 | 0 | 0 | 0 | 0 | 0 | 0 | 0 | 0 | 0 | 0 | 0 | 0 | 0 | 0 | 0 | 0 | 0 |
|  | **0** | 8 | 8 | 7 | 8 | 8 | 6 | 7 | 8 | 8 | 7 | 7 | 8 | 8 | 8 | 8 | 5 | 5 | 6 |
|  | **1** | 0 | 0 | 1 | 0 | 0 | 2 | 1 | 0 | 0 | 1 | 1 | 0 | 0 | 0 | 0 | 3 | 3 | 2 |
| **Tail elevation** | **-1** | 0 | 0 | 0 | 0 | 0 | 0 | 0 | 0 | 0 | 0 | 0 | 0 | 0 | 0 | 0 | 0 | 0 | 0 |
|  | **0** | 8 | 8 | 7 | 8 | 8 | 8 | 7 | 8 | 8 | 8 | 8 | 7 | 8 | 6 | 7 | 8 | 8 | 7 |
|  | **1** | 0 | 0 | 1 | 0 | 0 | 0 | 1 | 0 | 0 | 0 | 0 | 1 | 0 | 2 | 1 | 0 | 0 | 1 |
|  | **2** | 0 | 0 | 0 | 0 | 0 | 0 | 0 | 0 | 0 | 0 | 0 | 0 | 0 | 0 | 0 | 0 | 0 | 0 |
| **Limb rotation** | **0** | 8 | 8 | 8 | 8 | 8 | 7 | 8 | 8 | 7 | 8 | 8 | 7 | 8 | 8 | 4 | 5 | 5 | 4 |
|  | **1** | 0 | 0 | 0 | 0 | 0 | 1 | 0 | 0 | 1 | 0 | 0 | 1 | 0 | 0 | 4 | 3 | 3 | 4 |
|  | **2** | 0 | 0 | 0 | 0 | 0 | 0 | 0 | 0 | 0 | 0 | 0 | 0 | 0 | 0 | 0 | 0 | 0 | 0 |
| **Locomotor activity** | **-2** | 0 | 0 | 0 | 0 | 0 | 0 | 0 | 0 | 0 | 0 | 0 | 0 | 0 | 0 | 0 | 0 | 0 | 0 |
|  | **-1** | 0 | 0 | 0 | 0 | 0 | 1 | 0 | 0 | 0 | 0 | 0 | 0 | 0 | 0 | 3 | 0 | 0 | 0 |
|  | **0** | 8 | 8 | 8 | 8 | 8 | 7 | 8 | 8 | 7 | 8 | 8 | 8 | 8 | 8 | 5 | 8 | 8 | 8 |
|  | **1** | 0 | 0 | 0 | 0 | 0 | 0 | 0 | 0 | 1 | 0 | 0 | 0 | 0 | 0 | 0 | 0 | 0 | 0 |
|  | **2** | 0 | 0 | 0 | 0 | 0 | 0 | 0 | 0 | 0 | 0 | 0 | 0 | 0 | 0 | 0 | 0 | 0 | 0 |
| **Respiratory rate** | **-2** | 0 | 0 | 0 | 0 | 0 | 0 | 0 | 0 | 0 | 0 | 0 | 0 | 0 | 0 | 0 | 0 | 0 | 0 |
|  | **-1** | 0 | 0 | 0 | 0 | 0 | 0 | 0 | 0 | 0 | 0 | 0 | 0 | 0 | 0 | 0 | 0 | 0 | 0 |
|  | **0** | 8 | 8 | 8 | 8 | 8 | 8 | 8 | 7 | 8 | 8 | 8 | 8 | 8 | 8 | 8 | 8 | 8 | 8 |
|  | **1** | 0 | 0 | 0 | 0 | 0 | 0 | 0 | 1 | 0 | 0 | 0 | 0 | 0 | 0 | 0 | 0 | 0 | 0 |
|  | **2** | 0 | 0 | 0 | 0 | 0 | 0 | 0 | 0 | 0 | 0 | 0 | 0 | 0 | 0 | 0 | 0 | 0 | 0 |
| **Piloerection** | **0** | 8 | 8 | 8 | 8 | 8 | 8 | 8 | 8 | 8 | 8 | 8 | 8 | 8 | 8 | 8 | 8 | 8 | 8 |
|  | **2** | 0 | 0 | 0 | 0 | 0 | 0 | 0 | 0 | 0 | 0 | 0 | 0 | 0 | 0 | 0 | 0 | 0 | 0 |
| **Ataxia** | **0** | 8 | 8 | 8 | 8 | 8 | 8 | 8 | 8 | 8 | 8 | 8 | 8 | 8 | 8 | 8 | 8 | 8 | 8 |
|  | **1** | 0 | 0 | 0 | 0 | 0 | 0 | 0 | 0 | 0 | 0 | 0 | 0 | 0 | 0 | 0 | 0 | 0 | 0 |
|  | **2** | 0 | 0 | 0 | 0 | 0 | 0 | 0 | 0 | 0 | 0 | 0 | 0 | 0 | 0 | 0 | 0 | 0 | 0 |
| **Freezing** | **0** | 8 | 8 | 8 | 8 | 8 | 8 | 8 | 8 | 8 | 8 | 8 | 8 | 8 | 8 | 8 | 8 | 8 | 8 |
|  | **1** | 0 | 0 | 0 | 0 | 0 | 0 | 0 | 0 | 0 | 0 | 0 | 0 | 0 | 0 | 0 | 0 | 0 | 0 |
|  | **2** | 0 | 0 | 0 | 0 | 0 | 0 | 0 | 0 | 0 | 0 | 0 | 0 | 0 | 0 | 0 | 0 | 0 | 0 |
| **Vocalisation Handling** | **0** | 3 | 1 | 0 | 3 | 0 | 2 | 2 | 3 | 1 | 0 | 1 | 7 | 0 | 1 | 0 | 2 | 0 | 0 |
|  | **1** | 5 | 7 | 8 | 5 | 8 | 6 | 6 | 5 | 7 | 8 | 7 | 1 | 8 | 7 | 8 | 6 | 8 | 8 |
| **Curiosity** | **-2** | 0 | 0 | 0 | 0 | 0 | 0 | 0 | 0 | 0 | 0 | 0 | 0 | 0 | 0 | 0 | 0 | 0 | 0 |
|  | **-1** | 0 | 0 | 0 | 0 | 0 | 0 | 0 | 0 | 0 | 0 | 0 | 0 | 0 | 0 | 0 | 0 | 0 | 0 |
|  | **0** | 8 | 8 | 8 | 8 | 8 | 8 | 8 | 8 | 8 | 8 | 8 | 8 | 8 | 8 | 8 | 8 | 8 | 8 |
|  | **1** | 0 | 0 | 0 | 0 | 0 | 0 | 0 | 0 | 0 | 0 | 0 | 0 | 0 | 0 | 0 | 0 | 0 | 0 |
|  | **2** | 0 | 0 | 0 | 0 | 0 | 0 | 0 | 0 | 0 | 0 | 0 | 0 | 0 | 0 | 0 | 0 | 0 | 0 |
| **Startle** | **-2** | 0 | 0 | 0 | 0 | 0 | 0 | 0 | 0 | 0 | 0 | 0 | 0 | 0 | 0 | 0 | 0 | 0 | 0 |
|  | **-1** | 0 | 0 | 0 | 0 | 0 | 0 | 0 | 0 | 0 | 0 | 0 | 0 | 0 | 0 | 0 | 0 | 0 | 0 |
|  | **0** | 6 | 8 | 7 | 8 | 8 | 8 | 8 | 8 | 8 | 8 | 8 | 8 | 8 | 8 | 8 | 8 | 8 | 8 |
|  | **1** | 2 | 0 | 1 | 0 | 0 | 0 | 0 | 0 | 0 | 0 | 0 | 0 | 0 | 0 | 0 | 0 | 0 | 0 |
|  | **2** | 0 | 0 | 0 | 0 | 0 | 0 | 0 | 0 | 0 | 0 | 0 | 0 | 0 | 0 | 0 | 0 | 0 | 0 |
| **Touch reaction** | **-2** | 0 | 0 | 0 | 0 | 0 | 0 | 0 | 0 | 0 | 0 | 0 | 0 | 0 | 0 | 0 | 0 | 0 | 0 |
|  | **-1** | 0 | 0 | 0 | 0 | 0 | 1 | 0 | 0 | 0 | 0 | 0 | 0 | 0 | 0 | 0 | 0 | 0 | 0 |
|  | **0** | 3 | 2 | 0 | 1 | 2 | 4 | 4 | 6 | 4 | 0 | 5 | 1 | 1 | 0 | 5 | 3 | 3 | 2 |
|  | **1** | 5 | 6 | 8 | 6 | 6 | 3 | 4 | 2 | 4 | 6 | 2 | 4 | 4 | 6 | 3 | 3 | 4 | 5 |
|  | **2** | 0 | 0 | 0 | 1 | 0 | 0 | 0 | 0 | 0 | 2 | 1 | 3 | 3 | 2 | 0 | 2 | 1 | 1 |
| **Irritability** | **0** | 7 | 8 | 6 | 8 | 8 | 8 | 8 | 7 | 7 | 7 | 6 | 8 | 7 | 7 | 7 | 8 | 6 | 7 |
|  | **1** | 1 | 0 | 2 | 0 | 0 | 0 | 0 | 1 | 1 | 1 | 2 | 0 | 1 | 1 | 1 | 0 | 2 | 1 |
|  | **2** | 0 | 0 | 0 | 0 | 0 | 0 | 0 | 0 | 0 | 0 | 0 | 0 | 0 | 0 | 0 | 0 | 0 | 0 |
| **Body tone** | **-2** | 0 | 0 | 0 | 0 | 0 | 0 | 0 | 0 | 0 | 0 | 0 | 0 | 0 | 0 | 0 | 0 | 0 | 0 |
|  | **-1** | 0 | 0 | 0 | 0 | 0 | 0 | 0 | 0 | 0 | 0 | 0 | 0 | 0 | 0 | 0 | 0 | 0 | 0 |
|  | **0** | 8 | 8 | 6 | 8 | 8 | 8 | 8 | 8 | 8 | 8 | 8 | 8 | 8 | 8 | 8 | 8 | 8 | 8 |
|  | **1** | 0 | 0 | 2 | 0 | 0 | 0 | 0 | 0 | 0 | 0 | 0 | 0 | 0 | 0 | 0 | 0 | 0 | 0 |
|  | **2** | 0 | 0 | 0 | 0 | 0 | 0 | 0 | 0 | 0 | 0 | 0 | 0 | 0 | 0 | 0 | 0 | 0 | 0 |
| **Abdominal tone** | **-1** | 0 | 0 | 0 | 0 | 0 | 0 | 0 | 0 | 0 | 0 | 0 | 0 | 0 | 0 | 0 | 0 | 0 | 0 |
|  | **0** | 8 | 8 | 8 | 7 | 8 | 8 | 8 | 8 | 8 | 6 | 8 | 8 | 8 | 8 | 6 | 7 | 8 | 8 |
|  | **1** | 0 | 0 | 0 | 1 | 0 | 0 | 0 | 0 | 0 | 2 | 0 | 0 | 0 | 0 | 2 | 1 | 0 | 0 |
| **Urination** | **0** | 6 | 7 | 6 | 6 | 6 | 6 | 5 | 6 | 5 | 2 | 7 | 3 | 6 | 4 | 6 | 6 | 5 | 6 |
|  | **1** | 2 | 1 | 2 | 2 | 2 | 2 | 3 | 2 | 3 | 5 | 1 | 5 | 2 | 4 | 2 | 2 | 3 | 2 |
| **Defecation** | **0** | 5 | 3 | 6 | 3 | 5 | 6 | 4 | 5 | 4 | 4 | 7 | 4 | 3 | 4 | 4 | 3 | 5 | 6 |
|  | **1** | 3 | 5 | 2 | 5 | 3 | 2 | 4 | 3 | 4 | 4 | 1 | 4 | 5 | 4 | 4 | 5 | 3 | 2 |
| **Sex** | | **Males** | | | | | | | | | **Females** | | | | | | | | |
| **Experimental group** | | **NC.** | **Drug-control** | | | | **Surgery** | | | | **NC.** | **Drug-control** | | | | **Surgery** | | | |
| **Analgesic regimen** | | **no** | **N** | **NL** | **NO** | **NLO** | **N** | **NL** | **NO** | **NLO** | **no** | **N** | **NL** | **NO** | **NLO** | **N** | **NL** | **NO** | **NLO** |
| **Day 4** | | | | | | | | | | | | | | | | | | | |
| **Body position** | **-2** | 0 | 0 | 0 | 0 | 0 | 0 | 0 | 0 | 0 | 0 | 0 | 0 | 0 | 0 | 0 | 0 | 0 | 0 |
|  | **-1** | 0 | 0 | 0 | 0 | 0 | 0 | 0 | 0 | 0 | 0 | 0 | 0 | 0 | 0 | 0 | 0 | 0 | 0 |
|  | **0** | 8 | 8 | 8 | 8 | 8 | 8 | 8 | 8 | 8 | 8 | 8 | 8 | 8 | 8 | 8 | 8 | 8 | 8 |
|  | **1** | 0 | 0 | 0 | 0 | 0 | 0 | 0 | 0 | 0 | 0 | 0 | 0 | 0 | 0 | 0 | 0 | 0 | 0 |
|  | **2** | 0 | 0 | 0 | 0 | 0 | 0 | 0 | 0 | 0 | 0 | 0 | 0 | 0 | 0 | 0 | 0 | 0 | 0 |
| **Pelvic elevation** | **-1** | 0 | 0 | 0 | 0 | 0 | 0 | 0 | 0 | 0 | 0 | 0 | 0 | 0 | 0 | 0 | 0 | 0 | 0 |
|  | **0** | 8 | 8 | 7 | 8 | 8 | 7 | 7 | 8 | 8 | 7 | 7 | 8 | 8 | 8 | 8 | 5 | 5 | 6 |
|  | **1** | 0 | 0 | 1 | 0 | 0 | 1 | 1 | 0 | 0 | 1 | 1 | 0 | 0 | 0 | 0 | 3 | 3 | 2 |
| **Tail elevation** | **-1** | 0 | 0 | 0 | 0 | 0 | 0 | 0 | 0 | 0 | 0 | 0 | 0 | 0 | 0 | 0 | 0 | 0 | 0 |
|  | **0** | 8 | 8 | 7 | 8 | 8 | 8 | 7 | 8 | 7 | 8 | 7 | 7 | 8 | 7 | 7 | 7 | 4 | 5 |
|  | **1** | 0 | 0 | 1 | 0 | 0 | 0 | 1 | 0 | 1 | 0 | 1 | 1 | 0 | 1 | 1 | 1 | 4 | 3 |
|  | **2** | 0 | 0 | 0 | 0 | 0 | 0 | 0 | 0 | 0 | 0 | 0 | 0 | 0 | 0 | 0 | 0 | 0 | 0 |
| **Limb rotation** | **0** | 8 | 8 | 8 | 8 | 8 | 7 | 8 | 8 | 8 | 8 | 8 | 7 | 8 | 8 | 6 | 6 | 7 | 4 |
|  | **1** | 0 | 0 | 0 | 0 | 0 | 1 | 0 | 0 | 0 | 0 | 0 | 1 | 0 | 0 | 2 | 2 | 1 | 4 |
|  | **2** | 0 | 0 | 0 | 0 | 0 | 0 | 0 | 0 | 0 | 0 | 0 | 0 | 0 | 0 | 0 | 0 | 0 | 0 |
| **Locomotor activity** | **-2** | 0 | 0 | 0 | 0 | 0 | 0 | 0 | 0 | 0 | 0 | 0 | 0 | 0 | 0 | 0 | 0 | 0 | 0 |
|  | **-1** | 0 | 0 | 0 | 0 | 0 | 0 | 0 | 0 | 0 | 0 | 0 | 0 | 0 | 0 | 0 | 0 | 0 | 0 |
|  | **0** | 8 | 8 | 8 | 8 | 8 | 7 | 8 | 8 | 7 | 8 | 8 | 8 | 8 | 8 | 8 | 8 | 8 | 8 |
|  | **1** | 0 | 0 | 0 | 0 | 0 | 1 | 0 | 0 | 1 | 0 | 0 | 0 | 0 | 0 | 0 | 0 | 0 | 0 |
|  | **2** | 0 | 0 | 0 | 0 | 0 | 0 | 0 | 0 | 0 | 0 | 0 | 0 | 0 | 0 | 0 | 0 | 0 | 0 |
| **Respiratory rate** | **-2** | 0 | 0 | 0 | 0 | 0 | 0 | 0 | 0 | 0 | 0 | 0 | 0 | 0 | 0 | 0 | 0 | 0 | 0 |
|  | **-1** | 0 | 0 | 0 | 0 | 0 | 0 | 0 | 0 | 0 | 0 | 0 | 0 | 0 | 0 | 0 | 0 | 0 | 0 |
|  | **0** | 8 | 8 | 8 | 8 | 8 | 8 | 8 | 8 | 8 | 8 | 8 | 8 | 8 | 8 | 8 | 8 | 8 | 8 |
|  | **1** | 0 | 0 | 0 | 0 | 0 | 0 | 0 | 0 | 0 | 0 | 0 | 0 | 0 | 0 | 0 | 0 | 0 | 0 |
|  | **2** | 0 | 0 | 0 | 0 | 0 | 0 | 0 | 0 | 0 | 0 | 0 | 0 | 0 | 0 | 0 | 0 | 0 | 0 |
| **Piloerection** | **0** | 8 | 8 | 8 | 8 | 8 | 8 | 8 | 8 | 8 | 8 | 8 | 8 | 8 | 8 | 8 | 8 | 8 | 8 |
|  | **2** | 0 | 0 | 0 | 0 | 0 | 0 | 0 | 0 | 0 | 0 | 0 | 0 | 0 | 0 | 0 | 0 | 0 | 0 |
| **Ataxia** | **0** | 8 | 8 | 8 | 8 | 8 | 8 | 8 | 8 | 8 | 8 | 8 | 8 | 8 | 8 | 8 | 8 | 8 | 8 |
|  | **1** | 0 | 0 | 0 | 0 | 0 | 0 | 0 | 0 | 0 | 0 | 0 | 0 | 0 | 0 | 0 | 0 | 0 | 0 |
|  | **2** | 0 | 0 | 0 | 0 | 0 | 0 | 0 | 0 | 0 | 0 | 0 | 0 | 0 | 0 | 0 | 0 | 0 | 0 |
| **Freezing** | **0** | 7 | 8 | 8 | 8 | 8 | 8 | 8 | 8 | 8 | 8 | 8 | 8 | 8 | 8 | 8 | 8 | 8 | 8 |
|  | **1** | 1 | 0 | 0 | 0 | 0 | 0 | 0 | 0 | 0 | 0 | 0 | 0 | 0 | 0 | 0 | 0 | 0 | 0 |
|  | **2** | 0 | 0 | 0 | 0 | 0 | 0 | 0 | 0 | 0 | 0 | 0 | 0 | 0 | 0 | 0 | 0 | 0 | 0 |
| **Vocalisation Handling** | **0** | 2 | 1 | 1 | 0 | 1 | 3 | 1 | 2 | 4 | 0 | 0 | 0 | 1 | 1 | 0 | 1 | 0 | 0 |
|  | **1** | 6 | 7 | 7 | 8 | 7 | 5 | 7 | 6 | 4 | 8 | 8 | 8 | 7 | 7 | 8 | 7 | 8 | 8 |
| **Curiosity** | **-2** | 0 | 0 | 0 | 0 | 0 | 0 | 0 | 0 | 0 | 0 | 0 | 0 | 0 | 0 | 0 | 0 | 0 | 0 |
|  | **-1** | 1 | 0 | 0 | 0 | 0 | 0 | 0 | 0 | 0 | 0 | 0 | 0 | 0 | 0 | 0 | 0 | 0 | 0 |
|  | **0** | 7 | 8 | 8 | 8 | 8 | 8 | 8 | 8 | 8 | 8 | 8 | 8 | 8 | 8 | 8 | 8 | 8 | 8 |
|  | **1** | 0 | 0 | 0 | 0 | 0 | 0 | 0 | 0 | 0 | 0 | 0 | 0 | 0 | 0 | 0 | 0 | 0 | 0 |
|  | **2** | 0 | 0 | 0 | 0 | 0 | 0 | 0 | 0 | 0 | 0 | 0 | 0 | 0 | 0 | 0 | 0 | 0 | 0 |
| **Startle** | **-2** | 0 | 0 | 0 | 0 | 0 | 0 | 0 | 0 | 0 | 0 | 0 | 0 | 0 | 0 | 0 | 0 | 0 | 0 |
|  | **-1** | 0 | 0 | 0 | 0 | 0 | 0 | 0 | 0 | 0 | 0 | 0 | 0 | 0 | 0 | 0 | 0 | 0 | 0 |
|  | **0** | 7 | 8 | 8 | 8 | 8 | 8 | 8 | 8 | 8 | 8 | 8 | 8 | 8 | 8 | 8 | 8 | 8 | 8 |
|  | **1** | 1 | 0 | 0 | 0 | 0 | 0 | 0 | 0 | 0 | 0 | 0 | 0 | 0 | 0 | 0 | 0 | 0 | 0 |
|  | **2** | 0 | 0 | 0 | 0 | 0 | 0 | 0 | 0 | 0 | 0 | 0 | 0 | 0 | 0 | 0 | 0 | 0 | 0 |
| **Touch reaction** | **-2** | 0 | 0 | 0 | 0 | 0 | 0 | 0 | 0 | 0 | 0 | 0 | 0 | 0 | 0 | 0 | 0 | 0 | 0 |
|  | **-1** | 0 | 0 | 0 | 0 | 0 | 0 | 0 | 0 | 0 | 0 | 0 | 0 | 0 | 0 | 0 | 0 | 0 | 0 |
|  | **0** | 3 | 2 | 2 | 1 | 5 | 6 | 3 | 7 | 4 | 2 | 5 | 1 | 1 | 0 | 4 | 4 | 3 | 1 |
|  | **1** | 4 | 6 | 6 | 6 | 3 | 2 | 5 | 1 | 4 | 3 | 2 | 4 | 5 | 6 | 3 | 3 | 4 | 4 |
|  | **2** | 1 | 0 | 0 | 1 | 0 | 0 | 0 | 0 | 0 | 3 | 1 | 3 | 2 | 2 | 1 | 1 | 1 | 3 |
| **Irritability** | **0** | 7 | 8 | 6 | 8 | 8 | 8 | 8 | 7 | 7 | 7 | 3 | 7 | 7 | 6 | 7 | 7 | 8 | 7 |
|  | **1** | 1 | 0 | 2 | 0 | 0 | 0 | 0 | 1 | 1 | 1 | 5 | 1 | 1 | 2 | 1 | 1 | 0 | 1 |
|  | **2** | 0 | 0 | 0 | 0 | 0 | 0 | 0 | 0 | 0 | 0 | 0 | 0 | 0 | 0 | 0 | 0 | 0 | 0 |
| **Body tone** | **-2** | 0 | 0 | 0 | 0 | 0 | 0 | 0 | 0 | 0 | 0 | 0 | 0 | 0 | 0 | 0 | 0 | 0 | 0 |
|  | **-1** | 0 | 0 | 0 | 0 | 0 | 0 | 0 | 0 | 0 | 0 | 0 | 0 | 0 | 0 | 0 | 0 | 0 | 0 |
|  | **0** | 8 | 8 | 6 | 8 | 8 | 8 | 8 | 8 | 8 | 8 | 8 | 8 | 8 | 8 | 8 | 8 | 8 | 8 |
|  | **1** | 0 | 0 | 2 | 0 | 0 | 0 | 0 | 0 | 0 | 0 | 0 | 0 | 0 | 0 | 0 | 0 | 0 | 0 |
|  | **2** | 0 | 0 | 0 | 0 | 0 | 0 | 0 | 0 | 0 | 0 | 0 | 0 | 0 | 0 | 0 | 0 | 0 | 0 |
| **Abdominal tone** | **-1** | 0 | 0 | 0 | 0 | 0 | 0 | 0 | 0 | 0 | 0 | 0 | 0 | 0 | 0 | 0 | 0 | 0 | 0 |
|  | **0** | 8 | 8 | 8 | 8 | 8 | 8 | 8 | 8 | 8 | 6 | 8 | 5 | 8 | 8 | 6 | 7 | 8 | 8 |
|  | **1** | 0 | 0 | 0 | 0 | 0 | 0 | 0 | 0 | 0 | 2 | 0 | 3 | 0 | 0 | 2 | 1 | 0 | 0 |
| **Urination** | **0** | 4 | 7 | 7 | 6 | 4 | 7 | 5 | 2 | 5 | 3 | 1 | 3 | 4 | 4 | 4 | 4 | 3 | 3 |
|  | **1** | 4 | 1 | 1 | 2 | 4 | 1 | 3 | 6 | 3 | 5 | 7 | 5 | 4 | 4 | 4 | 4 | 5 | 5 |
| **Defecation** | **0** | 8 | 6 | 7 | 6 | 7 | 6 | 8 | 8 | 7 | 6 | 5 | 5 | 8 | 4 | 4 | 8 | 4 | 6 |
|  | **1** | 0 | 2 | 1 | 2 | 1 | 2 | 0 | 0 | 1 | 2 | 3 | 3 | 0 | 4 | 4 | 0 | 4 | 2 |

Please note that the following parameters, which were never altered during the course of the study, are not included in the table: Ptosis, Exophthalmia, Lacrimation, Hypersalivation, Skin perfusion, Stereotypies, Tremor, Twitches, Convulsions, Vocalisation, Feces, Implant area. NC. = naive-control group.

**Supplementary table 10: Statistical analyses of liquid intake**

ANOVA = analysis of variance, RM = repeated measures, vs = versus, see Fig. 5b.

| **Statistical test** | **Time point** | **Experimental group** | | | **Experimental group** | ***p*-value** |
| --- | --- | --- | --- | --- | --- | --- |
| **two-way RM ANOVA** | **Males: Naive-control vs Drug-control**  **Time:** *F*_2.326, 81.39_ = 18.91, *p* < 0.0001 | | | | | |
| **Mixed-effects model** | **Males: Naive-control vs Surgery**  **Time:** *F*_1.931, 67.2_ = 24.88, *p* < 0.0001 | | | | | |
| **two-way RM ANOVA** | **Females: Naive-control vs Drug-control**  **Time:** *F*_2.737, 95.78_ = 31.77, *p* < 0.0001  **Time x analgesic regimen:** *F*_20, 175_ = 4.117, *p* < 0.0001 | | | | | |
| Bonferroni *post hoc* test | Day -1 | | Naive-control | Surgery + N | | 0.0073 |
|  | Day -1 | | Naive-control | Surgery + NL | | 0.0324 |
|  | Day -1 | | Naive-control | Surgery + NO | | 0.007 |
|  | Day -1 | | Naive-control | Surgery + NLO | | 0.0187 |
| **two-way RM ANOVA** | **Females: Naive-control vs Surgery**  **Time:** *F*_2.816, 98.56_ = 41.57, *p* < 0.0001  **Time x analgesic regimen:** *F*_20, 175_ = 5.687, *p* < 0.0001 | | | | | |
| Bonferroni *post hoc* test | Day -1 | Naive-control | | | Surgery + NL | 0.0016 |
|  | Day -1 | Naive-control | | | Surgery + NO | 0.0072 |
|  | Day -1 | Naive-control | | | Surgery + NLO | 0.0373 |
|  | Day 0 | Naive-control | | | Surgery + NO | 0.0073 |
|  | Day 0 | Naive-control | | | Surgery + NLO | 0.006 |
|  | Day 0 | Surgery + N | | | Surgery + NO | 0.0203 |
|  | Day 0 | Surgery + N | | | Surgery + NLO | 0.016 |

**Supplementary table 11: Histopathological alterations**

| **Animals** | | | **Samples (n)** | | **Histopathological Alterations (cases)** | | | | |
| --- | --- | --- | --- | --- | --- | --- | --- | --- | --- |
| Subgroup | Experimental group | Sex | Stom  + DD | Skin | Stomach | | | DD | Skin |
|  |  |  |  |  | Focal inflammaion | Erosion | Ulcer |  |  |
| +N  (n = 32) | surgery | m | 8  (per subgroup  and  sex) | 0 | 0 | 0 | 1 | 0 | n.a. |
|  |  | f |  |  | 2 | 0 | 0 | 0 |  |
|  | drug-control | m |  |  | 0 | 0 | 0 | 0 |  |
|  |  | f |  |  | 0 | 2 | 0 | 0 |  |
| +NL  (n = 32) | surgery | m |  |  | 2 | 1 | 0 | 0 |  |
|  |  | f |  |  | 1 | 3 | 0 | 0 |  |
|  | drug-control | m |  |  | 0 | 2 | 0 | 0 |  |
|  |  | f |  |  | 2 | 1 | 0 | 0 |  |
| +NO  (n = 32) | surgery | m |  | 8  (per subgroup  and  sex) | 1 | 0 | 0 | 0 | 7 |
|  |  | f |  |  | 4 | 1 | 0 | 0 | 3 |
|  | drug-control | m |  |  | 0 | 0 | 1 | 0 | 6 |
|  |  | f |  |  | 0 | 1 | 0 | 1 | 6 |
| +NLO  (n = 32) | surgery | m |  |  | 1 | 1 | 1 | 0 | 4 |
|  |  | f |  |  | 2 | 0 | 0 | 0 | 4 |
|  | drug-control | m |  |  | 0 | 1 | 0 | 0 | 7 |
|  |  | f |  |  | 0 | 0 | 0 | 1 | 7 |
| none  (n = 16) | naive-control | m |  | 0 | 0 | 0 | 0 | 0 | n.a. |
|  |  | f |  | 5 | 0 | 0 | 0 | 0 | 0 |

m = males, f = females, stom = stomach, DD = duodenum, n.a. = not applicable, see Fig. 6.

**Supplementary table 12: Introduction to parameter names and pre-selection of CMS parameters**

| **Parameter name** | **Parameter description** | **Pre-selected for CMS** |
| --- | --- | --- |
| bwc | body weight change to mean baseline values in % | bwc_day1 |
| MGS | mean mouse grimace scale | MGS_day1 |
| bur | Burrowing behavior: removed pellets in g after 2 h, 4 h, 6 h and 20 h of providing the burrowing bottle | bur_exp_20h |
| bur_lat | Latency to onset of burrowing behavior in seconds |  |
| nest | Nest building behavior: nest score | nest_day1 |
| irwin | Modified Irwin sum score, also known as Neuro Score (urination, defecation and vocalization while handling were excluded from sum scores for CMS analyses) | irwin_day1 |
| FCM | Concentration of fecal corticosterone metabolites in ng/50 mg feces | FCM_day1 |
| distance_light | Distance travelled in the PhenoTyper home cage during 12-h light and darkphases in cm | distance-day1_light |
| distance_dark |  | distance-day1_dark |
| velocity_light | Mean velocity in the PhenoTyper home cage during 12-h light and darkphases in cm/s | velocity-day1_light |
| velocity_dark |  | velocity-day1_dark |
| VWR_dark | Distance travelled in the Voluntary Wheel Running during 12-h light and darkphases in m | VWR_day1-dark |
| VWR_light |  |  |
| Liquid intake | Water or carprofen-treated water intake corrected for bottle drips in g |  |

exp = measured during experimental phase (day 0 – day 4).

**Supplementary table 13: CMS Spearman correlation analyses**

| **Parameter 1** | **Parameter 2** | **Correlation coefficient *r*** | ***p*-value** |
| --- | --- | --- | --- |
| **Males** | | | |
| bwc_day1 | velocity_day1-dark | 0.51 | 5,66E-06 |
| distance-day1_light | velocity-day1_light | 0.91 | 0 |
| distance-day1_dark | velocity-day1_dark | 0.99 | 0 |
| distance-day1_dark | VWR_day1-dark | 0.78 | 1,33E-15 |
| velocity-day1_dark | VWR_day1-dark | 0.81 | 0 |
| velocity-day1_dark | FCM_day1 | 0.52 | 4,19E-06 |
| distance-day1_dark | FCM_day1 | 0.52 | 3,49E-06 |
| **Females** | | | |
| bwc_day1 | distance-day1_dark | 0.54 | 1,02E-06 |
| bwc_day1 | velocity-day1_dark | 0.52 | 2,78E-06 |
| bwc_day1 | FCM_day1 | 0.57 | 1,32E-07 |
| distance-day1_light | velocity-day1_light | 0.92 | 0 |
| distance-day1_dark | velocity-day1_dark | 0.99 | 0 |
| distance-day1_dark | VWR_day1-dark | 0.8 | 0 |
| velocity-day1_dark | VWR_day1-dark | 0.8 | 0 |

Results of correlated (r < -0,5 or > 0,5 in combination with p < 0.05) parameter pairs. See table S12 for explanation of parameters. See Fig. S6.

**Supplementary table 14: CMS top-ranking parameters**

| **Males** | | | | **Females** | | | |
| --- | --- | --- | --- | --- | --- | --- | --- |
| **P** | **Parameter** | **F** | **%** | **P** | **parameter** | **F** | **%** |
| 1 | nest_day1 | 79 | 79 | 1 | irwin_day1 | 86 | 86 |
|  | MGS_day1 | 9 | 9 |  | distance-day1_light | 13 | 13 |
|  | irwin_day1 | 8 | 8 |  | FCM_day1 | 1 | 1 |
|  | bwc_day1 | 3 | 3 | 2 | distance-day1_light | 45 | 45 |
|  | distance-day1_light | 1 | 1 |  | FCM_day1 | 39 | 39 |
| 2 | bwc_day1 | 55 | 55 |  | irwin_day1 | 11 | 11 |
|  | distance-day1_light | 19 | 19 |  | nest_day1 | 3 | 3 |
|  | MGS_day1 | 14 | 14 |  | bur_exp_20h | 1 | 1 |
|  | nest_day1 | 7 | 7 |  | distance-day1_dark | 1 | 1 |
|  | bur_exp_20h | 3 | 3 | 3 | FCM_day1 | 48 | 48 |
|  | irwin_day1 | 2 | 2 |  | distance-day1_light | 29 | 29 |
| 3 | MGS_day1 | 34 | 34 |  | nest_day1 | 8 | 8 |
|  | bwc_day1 | 29 | 29 |  | bwc_day1 | 7 | 7 |
|  | distance-day1_light | 17 | 17 |  | bur_exp_20h | 5 | 5 |
|  | nest_day1 | 6 | 6 |  | MGS_day1 | 2 | 2 |
|  | bur_exp_20h | 4 | 4 |  | irwin_day1 | 1 | 1 |
|  | distance-day1_dark | 4 | 4 | 4 | bwc_day1 | 39 | 39 |
|  | FCM_day1 | 4 | 4 |  | nest_day1 | 39 | 39 |
|  | irwin_day1 | 2 | 2 |  | FCM_day1 | 10 | 10 |
| 4 | FCM_day1 | 41 | 41 |  | bur_exp_20h | 4 | 4 |
|  | distance-day1_light | 18 | 18 |  | MGS_day1 | 4 | 4 |
|  | bur_exp_20h | 13 | 13 |  | distance-day1_light | 2 | 2 |
|  | distance-day1_dark | 9 | 9 |  | irwin_day1 | 2 | 2 |
|  | MGS_day1 | 9 | 9 | 5 | bwc_day1 | 49 | 49 |
|  | bwc_day1 | 6 | 6 |  | nest_day1 | 37 | 37 |
|  | irwin_day1 | 4 | 4 |  | distance-day1_dark | 6 | 6 |
| 5 | distance-day1_dark | 25 | 25 |  | bur_exp_20h | 3 | 3 |
|  | distance-day1_light | 14 | 14 |  | MGS_day1 | 3 | 3 |
|  | bur_exp_20h | 10 | 10 |  | distance-day1_light | 1 | 1 |
|  | bwc_day1 | 5 | 5 |  | FCM_day1 | 1 | 1 |

P = position, F = frequency, % = percentage. See table S12 for explanation of parameters. exp = measured during experimental phase (day 0 – day 4).

**Supplementary table 15:** **CMS allocation to clusters in male mice**

| **Experimental group** | **Analgesic regimen** | **Cluster** | **n** | **Frequency** |
| --- | --- | --- | --- | --- |
| Naive-control | none | 4 | 16 | 2,49221183800623 |
|  |  | 3 | 64 | 9,96884735202492 |
|  |  | 2 | 276 | 42,9906542056075 |
|  |  | 1 | 286 | 44,5482866043614 |
| Drug-control | N | 4 | 20 | 3,21543408360129 |
|  |  | 3 | 46 | 7,39549839228296 |
|  |  | 2 | 254 | 40,8360128617363 |
|  |  | 1 | 302 | 48,5530546623794 |
|  | NL | 4 | 12 | 1,9047619047619 |
|  |  | 3 | 120 | 19,047619047619 |
|  |  | 2 | 317 | 50,3174603174603 |
|  |  | 1 | 181 | 28,7301587301587 |
|  | NO | 4 | 9 | 1,42857142857143 |
|  |  | 3 | 309 | 49,047619047619 |
|  |  | 2 | 301 | 47,7777777777778 |
|  |  | 1 | 11 | 1,74603174603175 |
|  | NLO | 4 | 78 | 12,093023255814 |
|  |  | 3 | 175 | 27,1317829457364 |
|  |  | 2 | 258 | 40 |
|  |  | 1 | 134 | 20,7751937984496 |
| Surgery | N | 4 | 102 | 16,6394779771615 |
|  |  | 3 | 215 | 35,0734094616639 |
|  |  | 2 | 222 | 36,2153344208809 |
|  |  | 1 | 74 | 12,0717781402936 |
|  | NL | 4 | 195 | 30,0462249614792 |
|  |  | 3 | 306 | 47,1494607087827 |
|  |  | 2 | 134 | 20,6471494607088 |
|  |  | 1 | 14 | 2,15716486902928 |
|  | NO | 4 | 397 | 62,8164556962025 |
|  |  | 3 | 162 | 25,6329113924051 |
|  |  | 2 | 49 | 7,75316455696202 |
|  |  | 1 | 24 | 3,79746835443038 |
|  | NLO | 4 | 234 | 36,734693877551 |
|  |  | 3 | 327 | 51,3343799058085 |
|  |  | 2 | 70 | 10,989010989011 |
|  |  | 1 | 6 | 0,941915227629513 |

See Fig. 7c.

**Supplementary table 16: CMS allocation to clusters in female mice**

| **Experimental group** | **Analgesic regimen** | **Cluster** | **n** | **Frequency** |
| --- | --- | --- | --- | --- |
| Naive-control | none | 4 | 84 | 13,0841121495327 |
|  |  | 3 | 110 | 17,1339563862928 |
|  |  | 2 | 195 | 30,3738317757009 |
|  |  | 1 | 253 | 39,4080996884735 |
| Drug-control | N | 4 | 59 | 9,48553054662379 |
|  |  | 3 | 150 | 24,1157556270096 |
|  |  | 2 | 236 | 37,9421221864952 |
|  |  | 1 | 177 | 28,4565916398714 |
|  | NL | 4 | 61 | 9,68253968253968 |
|  |  | 3 | 158 | 25,0793650793651 |
|  |  | 2 | 244 | 38,7301587301587 |
|  |  | 1 | 167 | 26,5079365079365 |
|  | NO | 4 | 19 | 3,01587301587302 |
|  |  | 3 | 173 | 27,4603174603175 |
|  |  | 2 | 346 | 54,9206349206349 |
|  |  | 1 | 92 | 14,6031746031746 |
|  | NLO | 4 | 60 | 9,30232558139535 |
|  |  | 3 | 182 | 28,2170542635659 |
|  |  | 2 | 231 | 35,8139534883721 |
|  |  | 1 | 172 | 26,6666666666667 |
| Surgery | N | 4 | 169 | 27,5693311582382 |
|  |  | 3 | 191 | 31,1582381729201 |
|  |  | 2 | 163 | 26,5905383360522 |
|  |  | 1 | 90 | 14,6818923327896 |
|  | NL | 4 | 214 | 32,9738058551618 |
|  |  | 3 | 205 | 31,5870570107858 |
|  |  | 2 | 173 | 26,6563944530046 |
|  |  | 1 | 57 | 8,78274268104777 |
|  | NO | 4 | 284 | 44,9367088607595 |
|  |  | 3 | 168 | 26,5822784810127 |
|  |  | 2 | 97 | 15,3481012658228 |
|  |  | 1 | 83 | 13,1329113924051 |
|  | NLO | 4 | 200 | 31,3971742543171 |
|  |  | 3 | 250 | 39,2464678178964 |
|  |  | 2 | 133 | 20,8791208791209 |
|  |  | 1 | 54 | 8,47723704866562 |

See Fig. 7d.

**Supplementary table 17: Effect sizes (Cohen’s d)**

| **Parameter** | **Group comparision** | | | **Males** | | | | | **Females** | | | | |
| --- | --- | --- | --- | --- | --- | --- | --- | --- | --- | --- | --- | --- | --- |
|  |  |  |  | **n** | **d** | **se** | **lwr** | **upr** | **n** | **d** | **se** | **lwr** | **upr** |
| bwc_preOP | surgery-N | vs | nc | 16 | 0.364 | 0.918 | -0.718 | 1.445 | 16 | 0.184 | 1.322 | -0.891 | 1.258 |
| bwc_recovery | surgery-N | vs | nc | 16 | 0.422 | 0.693 | -0.662 | 1.506 | 16 | 0.095 | 1.013 | -0.978 | 1.168 |
| bwc_6h | surgery-N | vs | nc | 16 | 0.742 | 0.894 | -0.367 | 1.851 | 16 | 0.622 | 1.079 | -0.476 | 1.720 |
| bwc_day1 | surgery-N | vs | nc | 16 | -1.505 | 1.271 | -2.720 | -0.290 | 16 | -1.529 | 1.156 | -2.748 | -0.310 |
| bwc_day2 | surgery-N | vs | nc | 16 | -0.952 | 1.744 | -2.083 | 0.180 | 16 | -0.753 | 1.330 | -1.863 | 0.357 |
| bwc_day3 | surgery-N | vs | nc | 16 | -0.819 | 2.087 | -1.936 | 0.297 | 16 | -0.148 | 1.289 | -1.222 | 0.926 |
| bwc_day4 | surgery-N | vs | nc | 15 | -0.825 | 1.141 | -1.990 | 0.339 | 16 | -0.376 | 1.491 | -1.458 | 0.706 |
| bur_bl_2h | surgery-N | vs | nc | 16 | -0.010 | 7.206 | -1.083 | 1.062 | 16 | -0.527 | 57.051 | -1.618 | 0.564 |
| bur_bl_4h | surgery-N | vs | nc | 16 | -0.113 | 10.353 | -1.187 | 0.960 | 16 | -0.545 | 56.854 | -1.637 | 0.547 |
| bur_bl_6h | surgery-N | vs | nc | 16 | -0.133 | 13.347 | -1.207 | 0.940 | 16 | -0.545 | 56.888 | -1.638 | 0.547 |
| bur_bl_20h | surgery-N | vs | nc | 16 | 0.240 | 21.997 | -0.836 | 1.316 | 16 | -0.737 | 57.255 | -1.845 | 0.371 |
| bur_bl_lat | surgery-N | vs | nc | 16 | 0.262 | 1.318.573 | -0.815 | 1.339 | 16 | 0.285 | 243.023 | -0.793 | 1.363 |
| bur_exp_2h | surgery-N | vs | nc | 16 | -0.884 | 2.454 | -2.007 | 0.240 | 16 | -1.063 | 9.040 | -2.209 | 0.082 |
| bur_exp_4h | surgery-N | vs | nc | 16 | -0.957 | 2.422 | -2.089 | 0.175 | 16 | -1.048 | 11.860 | -2.192 | 0.095 |
| bur_exp_6h | surgery-N | vs | nc | 16 | -1.343 | 2.489 | -2.531 | -0.156 | 16 | -1.114 | 11.810 | -2.267 | 0.039 |
| bur_exp_20h | surgery-N | vs | nc | 16 | -0.458 | 16.743 | -1.544 | 0.628 | 16 | -1.569 | 15.949 | -2.796 | -0.343 |
| bur_exp_lat | surgery-N | vs | nc | 16 | 2.175 | 5.566.396 | 0.822 | 3.528 | 16 | 1.150 | 5.291.174 | -0.007 | 2.308 |
| liquid-intake_bl_mean/h | surgery-N | vs | nc | 16 | -0.111 | 0.036 | -1.184 | 0.963 | 16 | -0.284 | 0.009 | -1.361 | 0.794 |
| liquid-intake_day-1/h | surgery-N | vs | nc | 16 | 0.397 | 0.081 | -0.686 | 1.480 | 16 | 1.925 | 0.036 | 0.628 | 3.222 |
| liquid-intake_day0/h | surgery-N | vs | nc | 16 | -1.223 | 0.047 | -2.391 | -0.054 | 16 | -0.475 | 0.104 | -1.562 | 0.612 |
| liquid-intake_day1/h | surgery-N | vs | nc | 16 | 0.378 | 0.028 | -0.704 | 1.459 | 16 | -0.153 | 0.022 | -1.227 | 0.921 |
| liquid-intake_day2/h | surgery-N | vs | nc | 16 | -0.119 | 0.033 | -1.192 | 0.955 | 16 | 1.070 | 0.015 | -0.077 | 2.216 |
| liquid-intake_day3/h | surgery-N | vs | nc | 15 | 0.035 | 0.028 | -1.084 | 1.153 | 16 | -0.603 | 0.016 | -1.700 | 0.493 |
| MGS_bl | surgery-N | vs | nc | 16 | -0.417 | 0.047 | -1.501 | 0.667 | 16 | -0.110 | 0.066 | -1.183 | 0.964 |
| MGS_2h | surgery-N | vs | nc | 16 | 5.828 | 0.060 | 3.372 | 8.284 | 16 | 6.759 | 0.052 | 3.981 | 9.536 |
| MGS_4h | surgery-N | vs | nc | 16 | 3.938 | 0.077 | 2.100 | 5.777 | 16 | 5.009 | 0.071 | 2.828 | 7.190 |
| MGS_6h | surgery-N | vs | nc | 16 | 2.384 | 0.102 | 0.981 | 3.786 | 16 | 2.412 | 0.100 | 1.003 | 3.822 |
| MGS_8h | surgery-N | vs | nc | 16 | 1.998 | 0.119 | 0.685 | 3.310 | 16 | 2.646 | 0.109 | 1.178 | 4.115 |
| MGS_day1 | surgery-N | vs | nc | 16 | 3.264 | 0.043 | 1.627 | 4.902 | 16 | 2.181 | 0.047 | 0.826 | 3.535 |
| MGS_day2 | surgery-N | vs | nc | 16 | 1.693 | 0.084 | 0.443 | 2.943 | 16 | 1.496 | 0.053 | 0.283 | 2.710 |
| MGS_day3 | surgery-N | vs | nc | 16 | 0.449 | 0.070 | -0.637 | 1.535 | 16 | 0.303 | 0.069 | -0.776 | 1.381 |
| MGS_day4 | surgery-N | vs | nc | 15 | 0.750 | 0.071 | -0.406 | 1.907 | 16 | 0.892 | 0.055 | -0.232 | 2.017 |
| nest_bl_2h | surgery-N | vs | nc | 16 | 0.592 | 0.423 | -0.504 | 1.687 | 16 | 0.000 | 0.231 | -1.072 | 1.072 |
| nest_bl_4h | surgery-N | vs | nc | 16 | 0.303 | 0.412 | -0.775 | 1.382 | 16 | 0.500 | 0.250 | -0.589 | 1.589 |
| nest_bl_6h | surgery-N | vs | nc | 16 | 0.285 | 0.438 | -0.793 | 1.363 | 16 | 0.500 | 0.250 | -0.589 | 1.589 |
| nest_bl_day-3 | surgery-N | vs | nc | 16 | 0.249 | 0.754 | -0.828 | 1.325 | 16 | 0.000 | 0.320 | -1.072 | 1.072 |
| nest_exp_2h | surgery-N | vs | nc | 16 | -1.128 | 0.222 | -2.283 | 0.026 | 16 | -2.291 | 0.164 | -3.671 | -0.911 |
| nest_exp_4h | surgery-N | vs | nc | 16 | -1.128 | 0.222 | -2.283 | 0.026 | 16 | -2.291 | 0.164 | -3.671 | -0.911 |
| nest_exp_6h | surgery-N | vs | nc | 16 | -1.128 | 0.222 | -2.283 | 0.026 | 16 | -3.500 | 0.125 | -5.206 | -1.794 |
| nest_day1 | surgery-N | vs | nc | 16 | -0.428 | 0.730 | -1.512 | 0.657 | 16 | -1.379 | 0.453 | -2.572 | -0.186 |
| nest_day2 | surgery-N | vs | nc | 16 | 0.116 | 0.541 | -0.958 | 1.189 | 16 | -0.757 | 0.496 | -1.867 | 0.353 |
| nest_day3 | surgery-N | vs | nc | 16 | -0.364 | 0.515 | -1.445 | 0.717 | 16 | 0.000 | 0.401 | -1.072 | 1.072 |
| nest_day4 | surgery-N | vs | nc | 15 | -0.255 | 0.470 | -1.378 | 0.867 | 16 | 0.485 | 0.387 | -0.603 | 1.573 |
| distance_day0-20h | surgery-N | vs | nc | 16 | -1.380 | 8.656.131 | -2.573 | -0.187 | 16 | -2.333 | 4.441.536 | -3.723 | -0.943 |
| distance_day0_dark | surgery-N | vs | nc | 16 | -1.270 | 6.730.883 | -2.445 | -0.094 | 16 | -1.881 | 3.938.811 | -3.169 | -0.593 |
| distance_day1_light | surgery-N | vs | nc | 15 | -0.934 | 9.470.738 | -2.112 | 0.243 | 16 | -0.378 | 12.150.637 | -1.459 | 0.704 |
| distance_day1_dark | surgery-N | vs | nc | 15 | -0.496 | 13.467.693 | -1.631 | 0.639 | 16 | -0.917 | 18.876.662 | -2.045 | 0.210 |
| distance_day2_light | surgery-N | vs | nc | 15 | -0.074 | 3.105.225 | -1.192 | 1.045 | 16 | 0.429 | 5.284.884 | -0.655 | 1.514 |
| distance_day2_dark | surgery-N | vs | nc | 16 | 0.191 | 12.734.675 | -0.883 | 1.266 | 16 | 0.057 | 20.167.205 | -1.016 | 1.129 |
| distance_day3_light | surgery-N | vs | nc | 14 | -0.460 | 3.159.258 | -1.652 | 0.732 | 16 | -0.013 | 1.858.024 | -1.086 | 1.059 |
| distance_day3_dark | surgery-N | vs | nc | 14 | 0.421 | 10.275.924 | -0.768 | 1.610 | 16 | -0.442 | 17.097.983 | -1.527 | 0.644 |
| velocity_day0-20h | surgery-N | vs | nc | 16 | -0.990 | 0.147 | -2.126 | 0.146 | 16 | -1.592 | 0.084 | -2.823 | -0.362 |
| velocity_day0-dark | surgery-N | vs | nc | 16 | -1.043 | 0.199 | -2.186 | 0.100 | 16 | -1.505 | 0.107 | -2.720 | -0.290 |
| velocity_day1_light | surgery-N | vs | nc | 15 | -0.944 | 0.355 | -2.123 | 0.234 | 16 | -0.416 | 0.350 | -1.500 | 0.668 |
| velocity_day1_dark | surgery-N | vs | nc | 15 | -0.490 | 0.360 | -1.625 | 0.645 | 16 | -0.830 | 0.486 | -1.948 | 0.288 |
| velocity_day2_light | surgery-N | vs | nc | 15 | -0.158 | 0.077 | -1.277 | 0.962 | 16 | 0.093 | 0.042 | -0.980 | 1.166 |
| velocity_day2_dark | surgery-N | vs | nc | 16 | 0.131 | 0.355 | -0.942 | 1.205 | 16 | -0.018 | 0.502 | -1.090 | 1.055 |
| velocity_day3_light | surgery-N | vs | nc | 15 | -0.422 | 0.084 | -1.552 | 0.709 | 16 | -0.197 | 0.044 | -1.272 | 0.878 |
| velocity_day3_dark | surgery-N | vs | nc | 14 | 0.370 | 0.303 | -0.817 | 1.556 | 16 | -0.797 | 0.389 | -1.912 | 0.317 |
| VWR_day1_dark | surgery-N | vs | nc | 15 | -0.460 | 938.718 | -1.593 | 0.673 | 16 | -0.332 | 1.381.726 | -1.412 | 0.748 |
| VWR_day2_light | surgery-N | vs | nc | 16 | -0.360 | 64.787 | -1.441 | 0.721 | 16 | 0.814 | 237.722 | -0.302 | 1.929 |
| VWR_day2_dark | surgery-N | vs | nc | 16 | 0.268 | 868.504 | -0.809 | 1.346 | 16 | -0.106 | 1.469.502 | -1.180 | 0.967 |
| VWR_day3_light | surgery-N | vs | nc | 14 | -0.630 | 110.003 | -1.835 | 0.575 | 16 | -0.178 | 73.072 | -1.252 | 0.897 |
| VWR_day3_dark | surgery-N | vs | nc | 14 | 0.664 | 1.448.252 | -0.544 | 1.872 | 16 | -0.238 | 1.561.098 | -1.314 | 0.838 |
| FCM_bl-6 | surgery-N |  | nc |  |  |  |  |  | 15 | 0.226 | 15.259 | -0.895 | 1.348 |
| FCM_bl-3 | surgery-N | vs | nc | 16 | 0.165 | 8.694 | -0.909 | 1.240 | 16 | -0.146 | 14.532 | -1.220 | 0.928 |
| FCM_day1 | surgery-N | vs | nc | 16 | 1.027 | 13.932 | -0.114 | 2.168 | 16 | 1.087 | 29.810 | -0.062 | 2.236 |
| FCM_day4 | surgery-N | vs | nc | 15 | 0.910 | 10.421 | -0.264 | 2.085 | 16 | 0.612 | 16.057 | -0.486 | 1.709 |
| bwc_preOP | surgery-NL | vs | nc | 16 | -0.293 | 0.883 | -1.371 | 0.785 | 16 | 0.281 | 1.111 | -0.797 | 1.358 |
| bwc_recovery | surgery-NL | vs | nc | 16 | -0.546 | 0.638 | -1.638 | 0.546 | 16 | 0.220 | 1.426 | -0.855 | 1.296 |
| bwc_6h | surgery-NL | vs | nc | 16 | 0.816 | 0.752 | -0.300 | 1.932 | 16 | 0.628 | 1.050 | -0.470 | 1.727 |
| bwc_day1 | surgery-NL | vs | nc | 16 | -2.347 | 0.793 | -3.740 | -0.953 | 16 | -1.633 | 1.274 | -2.871 | -0.394 |
| bwc_day2 | surgery-NL | vs | nc | 16 | -0.942 | 0.983 | -2.072 | 0.188 | 16 | -1.529 | 1.176 | -2.748 | -0.310 |
| bwc_day3 | surgery-NL | vs | nc | 16 | -1.177 | 0.978 | -2.338 | -0.015 | 16 | -0.315 | 1.435 | -1.394 | 0.764 |
| bwc_day4 | surgery-NL | vs | nc | 16 | -1.622 | 0.822 | -2.858 | -0.386 | 16 | -0.337 | 1.713 | -1.416 | 0.743 |
| bur_bl_2h | surgery-NL | vs | nc | 16 | 0.115 | 6.628 | -0.958 | 1.188 | 16 | -0.615 | 55.588 | -1.712 | 0.483 |
| bur_bl_4h | surgery-NL | vs | nc | 16 | -0.075 | 8.805 | -1.147 | 0.998 | 16 | -0.630 | 55.376 | -1.729 | 0.468 |
| bur_bl_6h | surgery-NL | vs | nc | 16 | -0.215 | 10.654 | -1.291 | 0.860 | 16 | -0.623 | 55.484 | -1.721 | 0.475 |
| bur_bl_20h | surgery-NL | vs | nc | 16 | 0.407 | 11.639 | -0.676 | 1.490 | 16 | -0.783 | 56.294 | -1.896 | 0.329 |
| bur_bl_lat | surgery-NL | vs | nc | 16 | -0.756 | 424.892 | -1.866 | 0.354 | 16 | 0.114 | 252.530 | -0.959 | 1.187 |
| bur_exp_2h | surgery-NL | vs | nc | 16 | -0.881 | 2.455 | -2.004 | 0.242 | 16 | -0.909 | 8.426 | -2.035 | 0.218 |
| bur_exp_4h | surgery-NL | vs | nc | 16 | -0.954 | 2.418 | -2.085 | 0.178 | 16 | -0.916 | 11.413 | -2.043 | 0.212 |
| bur_exp_6h | surgery-NL | vs | nc | 16 | -0.975 | 2.717 | -2.110 | 0.159 | 16 | -0.983 | 11.363 | -2.118 | 0.152 |
| bur_exp_20h | surgery-NL | vs | nc | 16 | -0.670 | 14.281 | -1.773 | 0.432 | 16 | -1.744 | 13.907 | -3.004 | -0.484 |
| bur_exp_lat | surgery-NL | vs | nc | 16 | 0.026 | 438.544 | -1.047 | 1.098 | 16 | 1.799 | 4.030.087 | 0.528 | 3.070 |
| liquid-intake_bl_mean/h | surgery-NL | vs | nc | 16 | -0.104 | 0.032 | -1.177 | 0.969 | 16 | -0.218 | 0.013 | -1.294 | 0.857 |
| liquid-intake_day-1/h | surgery-NL | vs | nc | 16 | 0.371 | 0.085 | -0.710 | 1.453 | 16 | 2.948 | 0.032 | 1.399 | 4.497 |
| liquid-intake_day0/h | surgery-NL | vs | nc | 16 | -1.275 | 0.039 | -2.451 | -0.099 | 16 | -0.132 | 0.118 | -1.206 | 0.941 |
| liquid-intake_day1/h | surgery-NL | vs | nc | 16 | 0.426 | 0.034 | -0.658 | 1.511 | 16 | -0.468 | 0.025 | -1.555 | 0.619 |
| liquid-intake_day2/h | surgery-NL | vs | nc | 16 | -0.077 | 0.031 | -1.150 | 0.996 | 16 | 0.586 | 0.016 | -0.509 | 1.681 |
| liquid-intake_day3/h | surgery-NL | vs | nc | 16 | 0.042 | 0.025 | -1.030 | 1.115 | 16 | 0.012 | 0.021 | -1.060 | 1.085 |
| MGS_bl | surgery-NL | vs | nc | 16 | 0.495 | 0.042 | -0.594 | 1.584 | 16 | 0.232 | 0.066 | -0.844 | 1.308 |
| MGS_2h | surgery-NL | vs | nc | 16 | 4.078 | 0.078 | 2.196 | 5.960 | 16 | 5.825 | 0.066 | 3.370 | 8.281 |
| MGS_4h | surgery-NL | vs | nc | 15 | 3.030 | 0.090 | 1.393 | 4.667 | 16 | 5.315 | 0.072 | 3.033 | 7.598 |
| MGS_6h | surgery-NL | vs | nc | 16 | 2.277 | 0.127 | 0.900 | 3.653 | 16 | 4.297 | 0.069 | 2.346 | 6.247 |
| MGS_8h | surgery-NL | vs | nc | 16 | 2.182 | 0.137 | 0.828 | 3.536 | 16 | 2.686 | 0.106 | 1.207 | 4.165 |
| MGS_day1 | surgery-NL | vs | nc | 16 | 4.250 | 0.054 | 2.315 | 6.186 | 16 | 1.693 | 0.082 | 0.443 | 2.943 |
| MGS_day2 | surgery-NL | vs | nc | 16 | 2.322 | 0.061 | 0.935 | 3.710 | 16 | 1.321 | 0.067 | 0.138 | 2.505 |
| MGS_day3 | surgery-NL | vs | nc | 16 | 0.602 | 0.064 | -0.494 | 1.699 | 16 | 0.956 | 0.060 | -0.176 | 2.088 |
| MGS_day4 | surgery-NL | vs | nc | 16 | 1.446 | 0.064 | 0.242 | 2.651 | 16 | 1.871 | 0.036 | 0.585 | 3.157 |
| nest_bl_2h | surgery-NL | vs | nc | 16 | 0.635 | 0.590 | -0.464 | 1.735 | 16 | 0.000 | 0.231 | -1.072 | 1.072 |
| nest_bl_4h | surgery-NL | vs | nc | 16 | 0.519 | 0.482 | -0.571 | 1.609 | 16 | 0.000 | 0.231 | -1.072 | 1.072 |
| nest_bl_6h | surgery-NL | vs | nc | 16 | 0.639 | 0.489 | -0.460 | 1.739 | 16 | 0.224 | 0.280 | -0.852 | 1.299 |
| nest_bl_day-3 | surgery-NL | vs | nc | 16 | 0.162 | 0.773 | -0.913 | 1.236 | 16 | 0.458 | 0.409 | -0.628 | 1.545 |
| nest_exp_2h | surgery-NL | vs | nc | 16 | -1.708 | 0.183 | -2.961 | -0.455 | 16 | -2.291 | 0.164 | -3.671 | -0.911 |
| nest_exp_4h | surgery-NL | vs | nc | 16 | -1.128 | 0.222 | -2.283 | 0.026 | 16 | -1.517 | 0.206 | -2.734 | -0.300 |
| nest_exp_6h | surgery-NL | vs | nc | 16 | -1.128 | 0.222 | -2.283 | 0.026 | 16 | -2.121 | 0.177 | -3.462 | -0.781 |
| nest_day1 | surgery-NL | vs | nc | 16 | -0.888 | 0.563 | -2.012 | 0.236 | 16 | -2.036 | 0.460 | -3.357 | -0.715 |
| nest_day2 | surgery-NL | vs | nc | 16 | -0.252 | 0.496 | -1.329 | 0.824 | 16 | -1.110 | 0.507 | -2.262 | 0.042 |
| nest_day3 | surgery-NL | vs | nc | 16 | -0.471 | 0.398 | -1.558 | 0.616 | 16 | -1.128 | 0.443 | -2.283 | 0.026 |
| nest_day4 | surgery-NL | vs | nc | 16 | -0.316 | 0.395 | -1.395 | 0.763 | 16 | 0.000 | 0.496 | -1.072 | 1.072 |
| distance_day0-20h | surgery-NL | vs | nc | 16 | -1.645 | 8.551.252 | -2.885 | -0.404 | 16 | -2.281 | 5.100.276 | -3.658 | -0.903 |
| distance_day0_dark | surgery-NL | vs | nc | 16 | -1.611 | 6.736.851 | -2.846 | -0.377 | 16 | -1.726 | 4.761.528 | -2.982 | -0.470 |
| distance_day1_light | surgery-NL | vs | nc | 16 | -1.335 | 8.624.296 | -2.521 | -0.149 | 16 | -0.530 | 11.422.510 | -1.621 | 0.561 |
| distance_day1_dark | surgery-NL | vs | nc | 16 | -0.853 | 12.626.787 | -1.974 | 0.267 | 16 | -1.716 | 18.830.649 | -2.970 | -0.462 |
| distance_day2_light | surgery-NL | vs | nc | 16 | 0.605 | 2.419.177 | -0.492 | 1.701 | 16 | 0.994 | 2.161.422 | -0.142 | 2.131 |
| distance_day2_dark | surgery-NL | vs | nc | 16 | -0.448 | 11.229.478 | -1.533 | 0.638 | 16 | -1.249 | 19.379.436 | -2.421 | -0.076 |
| distance_day3_light | surgery-NL | vs | nc | 15 | 0.401 | 3.418.900 | -0.728 | 1.531 | 16 | 0.472 | 1.462.130 | -0.615 | 1.560 |
| distance_day3_dark | surgery-NL | vs | nc | 15 | -0.592 | 9.466.525 | -1.735 | 0.550 | 16 | -0.775 | 17.374.861 | -1.887 | 0.337 |
| velocity_day0-20h | surgery-NL | vs | nc | 16 | -1.424 | 0.143 | -2.625 | -0.223 | 16 | -1.488 | 0.103 | -2.700 | -0.277 |
| velocity_day0-dark | surgery-NL | vs | nc | 16 | -1.433 | 0.198 | -2.635 | -0.231 | 16 | -1.433 | 0.129 | -2.635 | -0.231 |
| velocity_day1_light | surgery-NL | vs | nc | 16 | -1.237 | 0.340 | -2.407 | -0.066 | 16 | -0.119 | 0.410 | -1.193 | 0.954 |
| velocity_day1_dark | surgery-NL | vs | nc | 16 | -0.794 | 0.350 | -1.908 | 0.319 | 16 | -1.593 | 0.484 | -2.824 | -0.363 |
| velocity_day2_light | surgery-NL | vs | nc | 16 | 0.461 | 0.064 | -0.625 | 1.548 | 16 | 1.064 | 0.063 | -0.082 | 2.210 |
| velocity_day2_dark | surgery-NL | vs | nc | 16 | -0.496 | 0.310 | -1.585 | 0.593 | 16 | -1.196 | 0.471 | -2.361 | -0.032 |
| velocity_day3_light | surgery-NL | vs | nc | 15 | 0.605 | 0.102 | -0.538 | 1.749 | 16 | 0.567 | 0.068 | -0.526 | 1.661 |
| velocity_day3_dark | surgery-NL | vs | nc | 15 | -0.764 | 0.250 | -1.922 | 0.394 | 16 | -0.611 | 0.504 | -1.708 | 0.487 |
| VWR_day1_dark | surgery-NL | vs | nc | 16 | -0.598 | 899.320 | -1.695 | 0.498 | 16 | -0.929 | 1.078.092 | -2.058 | 0.200 |
| VWR_day2_light | surgery-NL | vs | nc | 16 | 0.935 | 116.720 | -0.194 | 2.065 | 16 | 0.859 | 101.425 | -0.262 | 1.979 |
| VWR_day2_dark | surgery-NL | vs | nc | 16 | -0.230 | 674.774 | -1.306 | 0.846 | 16 | -0.857 | 1.290.442 | -1.977 | 0.264 |
| VWR_day3_light | surgery-NL | vs | nc | 15 | 0.589 | 184.786 | -0.553 | 1.731 | 16 | 0.291 | 82.584 | -0.787 | 1.369 |
| VWR_day3_dark | surgery-NL | vs | nc | 15 | -0.517 | 682.034 | -1.654 | 0.619 | 16 | -0.481 | 1.542.435 | -1.569 | 0.607 |
| FCM_bl-6 | surgery-NL |  | nc |  |  |  |  |  | 16 | 0.532 | 12.605 | -0.559 | 1.623 |
| FCM_bl-3 | surgery-NL | vs | nc | 16 | 0.253 | 6.411 | -0.823 | 1.330 | 16 | 0.280 | 14.178 | -0.798 | 1.357 |
| FCM_day1 | surgery-NL | vs | nc | 16 | 0.828 | 33.761 | -0.290 | 1.945 | 16 | 1.126 | 51.559 | -0.028 | 2.281 |
| FCM_day4 | surgery-NL | vs | nc | 16 | 0.845 | 3.845 | -0.274 | 1.964 | 16 | 0.698 | 13.218 | -0.407 | 1.802 |
| bwc_preOP | surgery-NO | vs | nc | 16 | 0.817 | 1.237 | -0.299 | 1.933 | 16 | -0.201 | 1.051 | -1.276 | 0.874 |
| bwc_recovery | surgery-NO | vs | nc | 16 | 1.303 | 0.727 | 0.122 | 2.484 | 16 | 0.394 | 0.870 | -0.689 | 1.477 |
| bwc_6h | surgery-NO | vs | nc | 16 | 0.535 | 0.887 | -0.557 | 1.626 | 16 | -0.361 | 0.924 | -1.442 | 0.720 |
| bwc_day1 | surgery-NO | vs | nc | 16 | -0.737 | 1.471 | -1.845 | 0.371 | 16 | -2.975 | 1.042 | -4.532 | -1.419 |
| bwc_day2 | surgery-NO | vs | nc | 16 | -0.531 | 1.531 | -1.622 | 0.560 | 16 | -1.751 | 1.098 | -3.012 | -0.490 |
| bwc_day3 | surgery-NO | vs | nc | 16 | 0.130 | 1.237 | -0.943 | 1.204 | 16 | -0.117 | 1.234 | -1.190 | 0.956 |
| bwc_day4 | surgery-NO | vs | nc | 16 | 0.264 | 1.197 | -0.813 | 1.342 | 16 | -0.122 | 1.525 | -1.195 | 0.951 |
| bur_bl_2h | surgery-NO | vs | nc | 16 | -0.724 | 5.558 | -1.831 | 0.383 | 16 | -0.576 | 56.418 | -1.670 | 0.519 |
| bur_bl_4h | surgery-NO | vs | nc | 16 | -0.942 | 7.083 | -2.073 | 0.188 | 16 | -0.592 | 56.225 | -1.688 | 0.503 |
| bur_bl_6h | surgery-NO | vs | nc | 16 | -0.854 | 9.342 | -1.974 | 0.266 | 16 | -0.591 | 56.271 | -1.687 | 0.504 |
| bur_bl_20h | surgery-NO | vs | nc | 16 | -0.320 | 13.608 | -1.399 | 0.760 | 16 | -0.655 | 57.927 | -1.756 | 0.446 |
| bur_bl_lat | surgery-NO | vs | nc | 16 | 0.612 | 7.205.810 | -0.485 | 1.709 | 16 | -0.131 | 279.567 | -1.205 | 0.943 |
| bur_exp_2h | surgery-NO | vs | nc | 16 | -0.827 | 2.455 | -1.945 | 0.290 | 16 | -0.941 | 8.402 | -2.071 | 0.189 |
| bur_exp_4h | surgery-NO | vs | nc | 16 | -0.915 | 2.418 | -2.042 | 0.212 | 16 | -0.938 | 11.395 | -2.068 | 0.192 |
| bur_exp_6h | surgery-NO | vs | nc | 16 | -1.293 | 2.485 | -2.472 | -0.114 | 16 | -0.933 | 11.417 | -2.062 | 0.196 |
| bur_exp_20h | surgery-NO | vs | nc | 16 | -0.881 | 14.095 | -2.004 | 0.243 | 16 | -1.262 | 15.545 | -2.436 | -0.087 |
| bur_exp_lat | surgery-NO | vs | nc | 15 | 2.041 | 10.682.110 | 0.663 | 3.418 | 16 | 2.890 | 2.916.391 | 1.357 | 4.424 |
| liquid-intake_bl_mean/h | surgery-NO | vs | nc | 16 | 0.163 | 0.038 | -0.911 | 1.238 | 16 | -1.138 | 0.008 | -2.294 | 0.018 |
| liquid-intake_day-1/h | surgery-NO | vs | nc | 16 | 0.460 | 0.090 | -0.627 | 1.546 | 16 | 2.492 | 0.032 | 1.063 | 3.921 |
| liquid-intake_day0/h | surgery-NO | vs | nc | 16 | -1.301 | 0.035 | -2.481 | -0.121 | 16 | -0.665 | 0.104 | -1.767 | 0.436 |
| liquid-intake_day1/h | surgery-NO | vs | nc | 16 | 0.926 | 0.024 | -0.202 | 2.055 | 16 | -0.568 | 0.022 | -1.662 | 0.525 |
| liquid-intake_day2/h | surgery-NO | vs | nc | 16 | 0.514 | 0.028 | -0.576 | 1.604 | 16 | 0.233 | 0.013 | -0.843 | 1.309 |
| liquid-intake_day3/h | surgery-NO | vs | nc | 16 | 0.705 | 0.022 | -0.400 | 1.810 | 16 | -1.251 | 0.011 | -2.424 | -0.079 |
| MGS_bl | surgery-NO | vs | nc | 16 | -0.442 | 0.043 | -1.527 | 0.644 | 16 | 0.242 | 0.045 | -0.835 | 1.318 |
| MGS_2h | surgery-NO | vs | nc | 16 | 2.266 | 0.127 | 0.892 | 3.641 | 16 | 7.559 | 0.050 | 4.499 | 10.620 |
| MGS_4h | surgery-NO | vs | nc | 16 | 5.696 | 0.063 | 3.285 | 8.107 | 16 | 6.834 | 0.063 | 4.029 | 9.638 |
| MGS_6h | surgery-NO | vs | nc | 16 | 3.463 | 0.094 | 1.768 | 5.158 | 16 | 3.333 | 0.094 | 1.676 | 4.991 |
| MGS_8h | surgery-NO | vs | nc | 16 | 3.273 | 0.082 | 1.633 | 4.913 | 16 | 2.712 | 0.122 | 1.226 | 4.198 |
| MGS_day1 | surgery-NO | vs | nc | 16 | 3.041 | 0.066 | 1.466 | 4.615 | 16 | 2.461 | 0.071 | 1.039 | 3.882 |
| MGS_day2 | surgery-NO | vs | nc | 16 | 3.419 | 0.046 | 1.737 | 5.102 | 16 | 0.783 | 0.065 | -0.330 | 1.895 |
| MGS_day3 | surgery-NO | vs | nc | 16 | 0.242 | 0.072 | -0.834 | 1.319 | 16 | 0.793 | 0.079 | -0.321 | 1.907 |
| MGS_day4 | surgery-NO | vs | nc | 16 | 0.934 | 0.070 | -0.196 | 2.063 | 16 | 0.599 | 0.076 | -0.497 | 1.695 |
| nest_bl_2h | surgery-NO | vs | nc | 16 | 0.276 | 0.453 | -0.802 | 1.353 | 16 | 0.500 | 0.250 | -0.589 | 1.589 |
| nest_bl_4h | surgery-NO | vs | nc | 16 | 0.162 | 0.387 | -0.913 | 1.236 | 16 | 0.500 | 0.250 | -0.589 | 1.589 |
| nest_bl_6h | surgery-NO | vs | nc | 16 | 0.316 | 0.395 | -0.763 | 1.395 | 16 | 0.556 | 0.337 | -0.537 | 1.649 |
| nest_bl_day-3 | surgery-NO | vs | nc | 16 | -0.249 | 0.754 | -1.325 | 0.828 | 16 | 0.000 | 0.458 | -1.072 | 1.072 |
| nest_exp_2h | surgery-NO | vs | nc | 16 | -1.708 | 0.183 | -2.961 | -0.455 | 16 | -2.291 | 0.164 | -3.671 | -0.911 |
| nest_exp_4h | surgery-NO | vs | nc | 16 | -1.708 | 0.183 | -2.961 | -0.455 | 16 | -2.291 | 0.164 | -3.671 | -0.911 |
| nest_exp_6h | surgery-NO | vs | nc | 16 | -1.708 | 0.183 | -2.961 | -0.455 | 16 | -3.500 | 0.125 | -5.206 | -1.794 |
| nest_day1 | surgery-NO | vs | nc | 16 | -1.789 | 0.524 | -3.058 | -0.520 | 16 | -1.126 | 0.611 | -2.280 | 0.028 |
| nest_day2 | surgery-NO | vs | nc | 16 | -0.809 | 0.541 | -1.924 | 0.306 | 16 | -0.993 | 0.441 | -2.129 | 0.144 |
| nest_day3 | surgery-NO | vs | nc | 16 | -0.342 | 0.549 | -1.422 | 0.739 | 16 | -0.485 | 0.387 | -1.573 | 0.603 |
| nest_day4 | surgery-NO | vs | nc | 16 | 0.136 | 0.460 | -0.938 | 1.209 | 16 | 0.720 | 0.347 | -0.387 | 1.827 |
| distance_day0-20h | surgery-NO | vs | nc | 16 | -1.238 | 8.555.790 | -2.409 | -0.068 | 16 | -1.913 | 4.898.175 | -3.207 | -0.618 |
| distance_day0_dark | surgery-NO | vs | nc | 16 | -1.576 | 6.840.551 | -2.803 | -0.348 | 16 | -2.038 | 4.221.488 | -3.360 | -0.717 |
| distance_day1_light | surgery-NO | vs | nc | 16 | -0.989 | 9.469.723 | -2.125 | 0.147 | 16 | -0.855 | 11.177.563 | -1.976 | 0.265 |
| distance_day1_dark | surgery-NO | vs | nc | 16 | -1.299 | 14.001.106 | -2.479 | -0.119 | 16 | -2.262 | 18.601.927 | -3.635 | -0.889 |
| distance_day2_light | surgery-NO | vs | nc | 16 | 0.057 | 2.533.437 | -1.016 | 1.130 | 16 | -0.324 | 1.354.534 | -1.404 | 0.755 |
| distance_day2_dark | surgery-NO | vs | nc | 16 | -0.377 | 13.443.400 | -1.459 | 0.705 | 16 | -0.620 | 22.976.445 | -1.718 | 0.478 |
| distance_day3_light | surgery-NO | vs | nc | 15 | 0.278 | 5.116.852 | -0.845 | 1.402 | 16 | -0.091 | 1.068.810 | -1.163 | 0.982 |
| distance_day3_dark | surgery-NO | vs | nc | 15 | 0.099 | 10.357.059 | -1.020 | 1.218 | 16 | 0.041 | 18.489.444 | -1.031 | 1.114 |
| velocity_day0-20h | surgery-NO | vs | nc | 16 | -1.039 | 0.145 | -2.182 | 0.103 | 16 | -1.390 | 0.091 | -2.585 | -0.195 |
| velocity_day0-dark | surgery-NO | vs | nc | 16 | -1.432 | 0.200 | -2.634 | -0.230 | 16 | -1.734 | 0.114 | -2.992 | -0.476 |
| velocity_day1_light | surgery-NO | vs | nc | 16 | -1.002 | 0.363 | -2.140 | 0.136 | 16 | -0.779 | 0.333 | -1.891 | 0.334 |
| velocity_day1_dark | surgery-NO | vs | nc | 16 | -1.190 | 0.385 | -2.354 | -0.027 | 16 | -2.270 | 0.468 | -3.645 | -0.895 |
| velocity_day2_light | surgery-NO | vs | nc | 16 | -0.036 | 0.068 | -1.109 | 1.036 | 16 | -0.234 | 0.036 | -1.310 | 0.842 |
| velocity_day2_dark | surgery-NO | vs | nc | 16 | -0.324 | 0.364 | -1.404 | 0.755 | 16 | -0.690 | 0.550 | -1.794 | 0.414 |
| velocity_day3_light | surgery-NO | vs | nc | 15 | 0.228 | 0.125 | -0.893 | 1.350 | 16 | -0.139 | 0.026 | -1.213 | 0.935 |
| velocity_day3_dark | surgery-NO | vs | nc | 15 | -0.077 | 0.277 | -1.195 | 1.042 | 16 | 0.059 | 0.525 | -1.014 | 1.131 |
| VWR_day1_dark | surgery-NO | vs | nc | 16 | -1.150 | 874.640 | -2.307 | 0.008 | 16 | -1.573 | 1.016.181 | -2.800 | -0.346 |
| VWR_day2_light | surgery-NO | vs | nc | 16 | -0.242 | 80.780 | -1.319 | 0.834 | 16 | -0.342 | 30.061 | -1.422 | 0.738 |
| VWR_day2_dark | surgery-NO | vs | nc | 16 | -0.458 | 708.168 | -1.545 | 0.628 | 16 | -0.383 | 1.480.377 | -1.465 | 0.699 |
| VWR_day3_light | surgery-NO | vs | nc | 15 | -0.102 | 177.790 | -1.221 | 1.017 | 16 | -0.233 | 64.839 | -1.310 | 0.843 |
| VWR_day3_dark | surgery-NO | vs | nc | 15 | -0.554 | 751.615 | -1.693 | 0.585 | 16 | 0.282 | 1.345.844 | -0.796 | 1.360 |
| FCM_bl-6 | surgery-NO |  | nc |  |  |  |  |  | 16 | 0.532 | 11.028 | -0.559 | 1.623 |
| FCM_bl-3 | surgery-NO | vs | nc | 16 | 1.095 | 12.218 | -0.055 | 2.245 | 16 | 0.052 | 13.856 | -1.021 | 1.124 |
| FCM_day1 | surgery-NO | vs | nc | 16 | 1.377 | 45.697 | 0.185 | 2.570 | 16 | 2.573 | 37.228 | 1.124 | 4.023 |
| FCM_day4 | surgery-NO | vs | nc | 16 | 2.031 | 3.170 | 0.710 | 3.351 | 16 | 0.841 | 10.644 | -0.277 | 1.960 |
| bwc_preOP | surgery-NLO | vs | nc | 16 | 0.119 | 1.171 | -0.954 | 1.192 | 16 | 0.309 | 0.974 | -0.770 | 1.388 |
| bwc_recovery | surgery-NLO | vs | nc | 16 | 0.039 | 0.708 | -1.034 | 1.111 | 16 | 0.796 | 0.868 | -0.318 | 1.910 |
| bwc_6h | surgery-NLO | vs | nc | 16 | -0.982 | 0.822 | -2.118 | 0.153 | 16 | -0.371 | 1.011 | -1.452 | 0.711 |
| bwc_day1 | surgery-NLO | vs | nc | 16 | -2.616 | 0.867 | -4.077 | -1.155 | 16 | -2.114 | 1.076 | -3.453 | -0.775 |
| bwc_day2 | surgery-NLO | vs | nc | 16 | -1.527 | 1.071 | -2.746 | -0.308 | 16 | -1.603 | 1.163 | -2.836 | -0.371 |
| bwc_day3 | surgery-NLO | vs | nc | 16 | -0.612 | 1.209 | -1.709 | 0.485 | 16 | -1.047 | 0.990 | -2.190 | 0.097 |
| bwc_day4 | surgery-NLO | vs | nc | 16 | -0.687 | 1.436 | -1.790 | 0.417 | 16 | -1.057 | 1.301 | -2.202 | 0.088 |
| bur_bl_2h | surgery-NLO | vs | nc | 16 | 0.014 | 7.436 | -1.058 | 1.087 | 16 | -0.691 | 55.318 | -1.795 | 0.413 |
| bur_bl_4h | surgery-NLO | vs | nc | 16 | -0.285 | 8.626 | -1.363 | 0.792 | 16 | -0.701 | 55.118 | -1.806 | 0.404 |
| bur_bl_6h | surgery-NLO | vs | nc | 16 | -0.302 | 10.545 | -1.380 | 0.777 | 16 | -0.700 | 55.164 | -1.805 | 0.404 |
| bur_bl_20h | surgery-NLO | vs | nc | 16 | 0.471 | 17.912 | -0.616 | 1.559 | 16 | -0.798 | 56.691 | -1.913 | 0.316 |
| bur_bl_lat | surgery-NLO | vs | nc | 16 | -0.638 | 515.262 | -1.737 | 0.462 | 16 | 0.433 | 210.083 | -0.652 | 1.518 |
| bur_exp_2h | surgery-NLO | vs | nc | 16 | -0.850 | 2.455 | -1.970 | 0.269 | 16 | -0.937 | 8.403 | -2.067 | 0.193 |
| bur_exp_4h | surgery-NLO | vs | nc | 16 | -0.924 | 2.421 | -2.053 | 0.204 | 16 | -0.936 | 11.393 | -2.065 | 0.194 |
| bur_exp_6h | surgery-NLO | vs | nc | 16 | -1.306 | 2.483 | -2.487 | -0.125 | 16 | -1.000 | 11.341 | -2.137 | 0.138 |
| bur_exp_20h | surgery-NLO | vs | nc | 16 | -1.060 | 13.469 | -2.206 | 0.085 | 16 | -1.889 | 13.413 | -3.179 | -0.599 |
| bur_exp_lat | surgery-NLO | vs | nc | 16 | 2.307 | 6.087.074 | 0.923 | 3.691 | 16 | 2.043 | 3.227.337 | 0.720 | 3.366 |
| liquid-intake_bl_mean/h | surgery-NLO | vs | nc | 16 | 0.127 | 0.038 | -0.947 | 1.200 | 16 | -0.447 | 0.009 | -1.533 | 0.639 |
| liquid-intake_day-1/h | surgery-NLO | vs | nc | 16 | 0.426 | 0.083 | -0.659 | 1.510 | 16 | 2.001 | 0.035 | 0.687 | 3.315 |
| liquid-intake_day0/h | surgery-NLO | vs | nc | 16 | -1.413 | 0.033 | -2.612 | -0.215 | 16 | -0.661 | 0.097 | -1.762 | 0.440 |
| liquid-intake_day1/h | surgery-NLO | vs | nc | 16 | 0.459 | 0.024 | -0.627 | 1.546 | 16 | -1.268 | 0.018 | -2.444 | -0.093 |
| liquid-intake_day2/h | surgery-NLO | vs | nc | 16 | 0.806 | 0.029 | -0.309 | 1.921 | 16 | -0.394 | 0.011 | -1.477 | 0.689 |
| liquid-intake_day3/h | surgery-NLO | vs | nc | 16 | 0.560 | 0.026 | -0.533 | 1.653 | 16 | -0.920 | 0.014 | -2.047 | 0.208 |
| MGS_bl | surgery-NLO | vs | nc | 16 | 0.349 | 0.043 | -0.732 | 1.429 | 16 | -0.043 | 0.068 | -1.115 | 1.030 |
| MGS_2h | surgery-NLO | vs | nc | 16 | 7.554 | 0.051 | 4.496 | 10.613 | 16 | 7.576 | 0.050 | 4.510 | 10.642 |
| MGS_4h | surgery-NLO | vs | nc | 16 | 4.029 | 0.094 | 2.162 | 5.895 | 16 | 6.713 | 0.061 | 3.951 | 9.474 |
| MGS_6h | surgery-NLO | vs | nc | 16 | 4.020 | 0.083 | 2.156 | 5.884 | 16 | 3.953 | 0.079 | 2.110 | 5.796 |
| MGS_8h | surgery-NLO | vs | nc | 16 | 4.463 | 0.076 | 2.459 | 6.466 | 16 | 4.456 | 0.085 | 2.455 | 6.457 |
| MGS_day1 | surgery-NLO | vs | nc | 16 | 4.147 | 0.045 | 2.243 | 6.050 | 16 | 2.945 | 0.041 | 1.397 | 4.494 |
| MGS_day2 | surgery-NLO | vs | nc | 16 | 2.615 | 0.045 | 1.155 | 4.076 | 16 | 1.005 | 0.088 | -0.133 | 2.143 |
| MGS_day3 | surgery-NLO | vs | nc | 16 | 0.661 | 0.073 | -0.441 | 1.762 | 16 | 0.684 | 0.060 | -0.419 | 1.787 |
| MGS_day4 | surgery-NLO | vs | nc | 16 | 1.460 | 0.057 | 0.253 | 2.667 | 16 | 1.337 | 0.053 | 0.151 | 2.524 |
| nest_bl_2h | surgery-NLO | vs | nc | 16 | 0.471 | 0.398 | -0.616 | 1.558 | 16 | 0.000 | 0.231 | -1.072 | 1.072 |
| nest_bl_4h | surgery-NLO | vs | nc | 16 | 0.000 | 0.354 | -1.072 | 1.072 | 16 | 0.000 | 0.231 | -1.072 | 1.072 |
| nest_bl_6h | surgery-NLO | vs | nc | 16 | 0.172 | 0.363 | -0.902 | 1.247 | 16 | 0.000 | 0.231 | -1.072 | 1.072 |
| nest_bl_day-3 | surgery-NLO | vs | nc | 16 | 0.234 | 0.800 | -0.842 | 1.310 | 16 | -0.713 | 0.263 | -1.819 | 0.393 |
| nest_exp_2h | surgery-NLO | vs | nc | 16 | -1.708 | 0.183 | -2.961 | -0.455 | 16 | -2.291 | 0.164 | -3.671 | -0.911 |
| nest_exp_4h | surgery-NLO | vs | nc | 16 | -1.708 | 0.183 | -2.961 | -0.455 | 16 | -2.291 | 0.164 | -3.671 | -0.911 |
| nest_exp_6h | surgery-NLO | vs | nc | 16 | -1.708 | 0.183 | -2.961 | -0.455 | 16 | -3.500 | 0.125 | -5.206 | -1.794 |
| nest_day1 | surgery-NLO | vs | nc | 16 | -0.983 | 0.699 | -2.119 | 0.152 | 16 | -1.844 | 0.441 | -3.124 | -0.564 |
| nest_day2 | surgery-NLO | vs | nc | 16 | -0.569 | 0.549 | -1.663 | 0.525 | 16 | -1.434 | 0.479 | -2.636 | -0.232 |
| nest_day3 | surgery-NLO | vs | nc | 16 | -0.425 | 0.441 | -1.510 | 0.659 | 16 | -1.040 | 0.541 | -2.183 | 0.102 |
| nest_day4 | surgery-NLO | vs | nc | 16 | 0.167 | 0.375 | -0.908 | 1.241 | 16 | -0.262 | 0.477 | -1.339 | 0.815 |
| distance_day0-20h | surgery-NLO | vs | nc | 16 | -1.645 | 8.265.628 | -2.886 | -0.405 | 16 | -2.103 | 5.083.437 | -3.439 | -0.766 |
| distance_day0_dark | surgery-NLO | vs | nc | 16 | -1.787 | 6.482.679 | -3.055 | -0.518 | 16 | -2.366 | 4.014.137 | -3.765 | -0.968 |
| distance_day1_light | surgery-NLO | vs | nc | 16 | -1.089 | 9.484.765 | -2.238 | 0.060 | 16 | -0.524 | 11.813.045 | -1.615 | 0.566 |
| distance_day1_dark | surgery-NLO | vs | nc | 16 | -1.264 | 11.413.618 | -2.438 | -0.089 | 16 | -1.754 | 18.407.632 | -3.016 | -0.492 |
| distance_day2_light | surgery-NLO | vs | nc | 16 | 0.290 | 2.789.053 | -0.788 | 1.368 | 16 | 0.546 | 3.071.070 | -0.546 | 1.639 |
| distance_day2_dark | surgery-NLO | vs | nc | 16 | 0.266 | 10.915.832 | -0.811 | 1.343 | 16 | 0.318 | 21.848.063 | -0.761 | 1.398 |
| distance_day3_light | surgery-NLO | vs | nc | 15 | 0.163 | 3.932.262 | -0.957 | 1.283 | 16 | 0.973 | 2.975.463 | -0.161 | 2.108 |
| distance_day3_dark | surgery-NLO | vs | nc | 15 | 0.425 | 12.299.686 | -0.706 | 1.556 | 16 | 0.471 | 17.373.833 | -0.616 | 1.558 |
| velocity_day0-20h | surgery-NLO | vs | nc | 16 | -1.492 | 0.133 | -2.704 | -0.279 | 16 | -1.494 | 0.098 | -2.707 | -0.282 |
| velocity_day0-dark | surgery-NLO | vs | nc | 16 | -1.590 | 0.192 | -2.820 | -0.360 | 16 | -2.231 | 0.102 | -3.597 | -0.865 |
| velocity_day1_light | surgery-NLO | vs | nc | 16 | -1.385 | 0.332 | -2.579 | -0.191 | 16 | -0.533 | 0.346 | -1.624 | 0.559 |
| velocity_day1_dark | surgery-NLO | vs | nc | 16 | -1.218 | 0.310 | -2.386 | -0.050 | 16 | -1.784 | 0.466 | -3.052 | -0.516 |
| velocity_day2_light | surgery-NLO | vs | nc | 16 | 0.230 | 0.076 | -0.846 | 1.306 | 16 | 0.492 | 0.077 | -0.596 | 1.581 |
| velocity_day2_dark | surgery-NLO | vs | nc | 16 | 0.110 | 0.303 | -0.964 | 1.183 | 16 | 0.252 | 0.520 | -0.824 | 1.329 |
| velocity_day3_light | surgery-NLO | vs | nc | 15 | 0.156 | 0.105 | -0.964 | 1.276 | 16 | 0.899 | 0.072 | -0.227 | 2.024 |
| velocity_day3_dark | surgery-NLO | vs | nc | 15 | 0.192 | 0.303 | -0.928 | 1.313 | 16 | 0.285 | 0.444 | -0.793 | 1.362 |
| VWR_day1_dark | surgery-NLO | vs | nc | 16 | -1.190 | 828.808 | -2.354 | -0.027 | 16 | -0.912 | 1.168.533 | -2.038 | 0.215 |
| VWR_day2_light | surgery-NLO | vs | nc | 16 | 0.334 | 84.222 | -0.746 | 1.413 | 16 | 0.679 | 42.494 | -0.424 | 1.782 |
| VWR_day2_dark | surgery-NLO | vs | nc | 16 | -0.084 | 623.914 | -1.156 | 0.989 | 16 | 0.213 | 1.409.284 | -0.863 | 1.288 |
| VWR_day3_light | surgery-NLO | vs | nc | 15 | -0.011 | 145.644 | -1.129 | 1.107 | 16 | 0.661 | 94.423 | -0.441 | 1.762 |
| VWR_day3_dark | surgery-NLO | vs | nc | 15 | -0.006 | 775.496 | -1.124 | 1.112 | 16 | 0.493 | 1.394.849 | -0.596 | 1.581 |
| FCM_bl-6 | surgery-NLO |  | nc |  |  |  |  |  | 16 | -0.020 | 8.251 | -1.093 | 1.052 |
| FCM_bl-3 | surgery-NLO | vs | nc | 16 | 0.243 | 5.662 | -0.834 | 1.319 | 16 | -0.231 | 12.231 | -1.307 | 0.845 |
| FCM_day1 | surgery-NLO | vs | nc | 16 | 1.798 | 22.006 | 0.527 | 3.069 | 16 | 2.000 | 51.916 | 0.687 | 3.314 |
| FCM_day4 | surgery-NLO | vs | nc | 16 | 1.631 | 4.561 | 0.393 | 2.869 | 16 | 0.435 | 15.923 | -0.650 | 1.520 |
| bwc_preOP | drug-control-N | vs | nc | 16 | -0.122 | 1.432 | -1.196 | 0.951 | 16 | 0.336 | 1.007 | -0.744 | 1.416 |
| bwc_recovery | drug-control-N | vs | nc | 16 | 0.673 | 0.844 | -0.429 | 1.776 | 16 | 0.788 | 0.841 | -0.325 | 1.902 |
| bwc_6h | drug-control-N | vs | nc | 16 | 0.612 | 0.689 | -0.485 | 1.710 | 16 | 1.139 | 0.689 | -0.017 | 2.295 |
| bwc_day1 | drug-control-N | vs | nc | 16 | -0.859 | 0.845 | -1.980 | 0.262 | 16 | -0.496 | 1.029 | -1.585 | 0.593 |
| bwc_day2 | drug-control-N | vs | nc | 16 | -0.011 | 0.957 | -1.083 | 1.061 | 16 | -0.431 | 1.001 | -1.516 | 0.654 |
| bwc_day3 | drug-control-N | vs | nc | 16 | -0.290 | 0.848 | -1.368 | 0.788 | 16 | 0.277 | 1.087 | -0.800 | 1.355 |
| bwc_day4 | drug-control-N | vs | nc | 16 | -0.109 | 0.881 | -1.182 | 0.964 | 16 | -0.476 | 1.203 | -1.564 | 0.611 |
| bur_bl_2h | drug-control-N | vs | nc | 16 | 0.519 | 14.703 | -0.572 | 1.609 | 16 | -0.463 | 56.238 | -1.550 | 0.624 |
| bur_bl_4h | drug-control-N | vs | nc | 16 | 0.326 | 15.336 | -0.754 | 1.405 | 16 | -0.481 | 56.027 | -1.569 | 0.607 |
| bur_bl_6h | drug-control-N | vs | nc | 16 | 0.201 | 16.488 | -0.874 | 1.276 | 16 | -0.477 | 56.053 | -1.564 | 0.611 |
| bur_bl_20h | drug-control-N | vs | nc | 16 | 0.278 | 17.806 | -0.799 | 1.356 | 16 | -0.613 | 56.269 | -1.710 | 0.484 |
| bur_bl_lat | drug-control-N | vs | nc | 16 | -0.335 | 576.364 | -1.415 | 0.745 | 16 | -0.080 | 184.919 | -1.153 | 0.992 |
| bur_exp_2h | drug-control-N | vs | nc | 16 | -0.826 | 2.466 | -1.943 | 0.291 | 16 | -0.885 | 8.421 | -2.008 | 0.239 |
| bur_exp_4h | drug-control-N | vs | nc | 16 | -0.839 | 2.429 | -1.958 | 0.280 | 16 | -0.829 | 11.436 | -1.947 | 0.288 |
| bur_exp_6h | drug-control-N | vs | nc | 16 | -0.420 | 3.857 | -1.504 | 0.664 | 16 | -0.845 | 11.404 | -1.964 | 0.274 |
| bur_exp_20h | drug-control-N | vs | nc | 16 | 0.390 | 19.589 | -0.693 | 1.472 | 16 | -0.836 | 16.641 | -1.954 | 0.282 |
| bur_exp_lat | drug-control-N | vs | nc | 16 | 1.143 | 5.281.856 | -0.013 | 2.300 | 16 | 0.680 | 2.515.877 | -0.423 | 1.783 |
| liquid-intake_bl_mean/h | drug-control-N | vs | nc | 16 | -0.377 | 0.031 | -1.459 | 0.704 | 16 | -1.239 | 0.008 | -2.410 | -0.068 |
| liquid-intake_day-1/h | drug-control-N | vs | nc | 16 | 0.295 | 0.086 | -0.784 | 1.373 | 16 | 2.416 | 0.027 | 1.005 | 3.826 |
| liquid-intake_day0/h | drug-control-N | vs | nc | 16 | -0.644 | 0.037 | -1.744 | 0.456 | 16 | -0.565 | 0.092 | -1.659 | 0.528 |
| liquid-intake_day1/h | drug-control-N | vs | nc | 16 | 0.412 | 0.026 | -0.671 | 1.496 | 16 | -0.568 | 0.019 | -1.661 | 0.526 |
| liquid-intake_day2/h | drug-control-N | vs | nc | 16 | -0.091 | 0.029 | -1.164 | 0.982 | 16 | 0.173 | 0.014 | -0.901 | 1.248 |
| liquid-intake_day3/h | drug-control-N | vs | nc | 16 | -0.244 | 0.026 | -1.321 | 0.832 | 16 | -0.787 | 0.015 | -1.900 | 0.326 |
| MGS_bl | drug-control-N | vs | nc | 16 | 0.160 | 0.047 | -0.914 | 1.234 | 16 | 0.122 | 0.045 | -0.951 | 1.196 |
| MGS_2h | drug-control-N | vs | nc | 16 | 2.141 | 0.072 | 0.796 | 3.486 | 16 | 1.970 | 0.077 | 0.663 | 3.277 |
| MGS_4h | drug-control-N | vs | nc | 16 | 1.056 | 0.066 | -0.089 | 2.200 | 16 | 1.818 | 0.079 | 0.544 | 3.093 |
| MGS_6h | drug-control-N | vs | nc | 16 | 0.601 | 0.082 | -0.495 | 1.697 | 16 | 0.418 | 0.083 | -0.666 | 1.502 |
| MGS_8h | drug-control-N | vs | nc | 16 | 0.227 | 0.079 | -0.849 | 1.303 | 16 | 0.479 | 0.078 | -0.609 | 1.567 |
| MGS_day1 | drug-control-N | vs | nc | 16 | 0.995 | 0.030 | -0.142 | 2.132 | 16 | 1.295 | 0.043 | 0.116 | 2.475 |
| MGS_day2 | drug-control-N | vs | nc | 16 | 1.203 | 0.030 | 0.037 | 2.368 | 16 | 0.403 | 0.066 | -0.680 | 1.486 |
| MGS_day3 | drug-control-N | vs | nc | 16 | -0.593 | 0.066 | -1.688 | 0.503 | 16 | -0.353 | 0.063 | -1.434 | 0.727 |
| MGS_day4 | drug-control-N | vs | nc | 16 | -0.582 | 0.043 | -1.676 | 0.513 | 16 | -0.231 | 0.057 | -1.307 | 0.845 |
| nest_bl_2h | drug-control-N | vs | nc | 16 | 0.635 | 0.590 | -0.464 | 1.735 | 16 | 0.764 | 0.164 | -0.347 | 1.875 |
| nest_bl_4h | drug-control-N | vs | nc | 16 | 0.483 | 0.518 | -0.605 | 1.571 | 16 | 0.764 | 0.164 | -0.347 | 1.875 |
| nest_bl_6h | drug-control-N | vs | nc | 16 | 0.596 | 0.524 | -0.500 | 1.692 | 16 | 0.910 | 0.206 | -0.216 | 2.037 |
| nest_bl_day-3 | drug-control-N | vs | nc | 16 | 0.157 | 0.796 | -0.917 | 1.231 | 16 | 0.632 | 0.395 | -0.466 | 1.731 |
| nest_exp_2h | drug-control-N | vs | nc | 16 | -0.193 | 0.324 | -1.268 | 0.882 | 16 | 0.224 | 0.280 | -0.852 | 1.299 |
| nest_exp_4h | drug-control-N | vs | nc | 16 | 0.000 | 0.320 | -1.072 | 1.072 | 16 | 0.342 | 0.366 | -0.739 | 1.422 |
| nest_exp_6h | drug-control-N | vs | nc | 16 | 0.202 | 0.310 | -0.873 | 1.277 | 16 | -0.224 | 0.280 | -1.299 | 0.852 |
| nest_day1 | drug-control-N | vs | nc | 16 | -0.222 | 0.563 | -1.298 | 0.854 | 16 | 0.111 | 0.565 | -0.963 | 1.184 |
| nest_day2 | drug-control-N | vs | nc | 16 | 0.000 | 0.594 | -1.072 | 1.072 | 16 | 0.000 | 0.458 | -1.072 | 1.072 |
| nest_day3 | drug-control-N | vs | nc | 16 | -0.117 | 0.532 | -1.191 | 0.956 | 16 | 0.605 | 0.310 | -0.491 | 1.702 |
| nest_day4 | drug-control-N | vs | nc | 16 | -0.524 | 0.477 | -1.615 | 0.567 | 16 | 0.485 | 0.387 | -0.603 | 1.573 |
| distance_day0-20h | drug-control-N | vs | nc | 16 | -0.038 | 12.900.496 | -1.110 | 1.035 | 16 | -0.512 | 5.997.168 | -1.601 | 0.578 |
| distance_day0_dark | drug-control-N | vs | nc | 16 | -0.312 | 9.059.424 | -1.391 | 0.767 | 16 | -0.439 | 5.468.045 | -1.524 | 0.646 |
| distance_day1_light | drug-control-N | vs | nc | 16 | -0.166 | 9.997.018 | -1.241 | 0.908 | 16 | 0.282 | 14.536.154 | -0.796 | 1.360 |
| distance_day1_dark | drug-control-N | vs | nc | 16 | 0.452 | 18.418.629 | -0.634 | 1.538 | 16 | 0.172 | 29.359.096 | -0.903 | 1.246 |
| distance_day2_light | drug-control-N | vs | nc | 16 | 0.596 | 3.713.197 | -0.500 | 1.691 | 16 | 0.441 | 3.393.460 | -0.644 | 1.527 |
| distance_day2_dark | drug-control-N | vs | nc | 16 | 0.956 | 14.396.930 | -0.176 | 2.088 | 16 | 0.211 | 29.459.745 | -0.864 | 1.287 |
| distance_day3_light | drug-control-N | vs | nc | 15 | 0.668 | 8.957.686 | -0.481 | 1.816 | 16 | 0.388 | 2.236.038 | -0.695 | 1.470 |
| distance_day3_dark | drug-control-N | vs | nc | 15 | 0.778 | 12.201.363 | -0.382 | 1.937 | 16 | 0.291 | 26.237.647 | -0.787 | 1.369 |
| velocity_day0-20h | drug-control-N | vs | nc | 16 | 0.177 | 0.226 | -0.898 | 1.251 | 16 | -0.483 | 0.113 | -1.571 | 0.605 |
| velocity_day0-dark | drug-control-N | vs | nc | 16 | -0.336 | 0.251 | -1.416 | 0.743 | 16 | -0.447 | 0.134 | -1.533 | 0.639 |
| velocity_day1_light | drug-control-N | vs | nc | 16 | -0.355 | 0.377 | -1.436 | 0.726 | 16 | 0.327 | 0.438 | -0.753 | 1.406 |
| velocity_day1_dark | drug-control-N | vs | nc | 16 | 0.348 | 0.464 | -0.732 | 1.429 | 16 | 0.140 | 0.728 | -0.934 | 1.214 |
| velocity_day2_light | drug-control-N | vs | nc | 16 | 0.525 | 0.094 | -0.566 | 1.616 | 16 | 0.482 | 0.089 | -0.606 | 1.570 |
| velocity_day2_dark | drug-control-N | vs | nc | 16 | 0.769 | 0.371 | -0.342 | 1.880 | 16 | 0.303 | 0.800 | -0.775 | 1.382 |
| velocity_day3_light | drug-control-N | vs | nc | 15 | 0.640 | 0.227 | -0.506 | 1.787 | 16 | 0.461 | 0.070 | -0.625 | 1.548 |
| velocity_day3_dark | drug-control-N | vs | nc | 15 | 0.483 | 0.310 | -0.651 | 1.617 | 16 | 0.194 | 0.818 | -0.881 | 1.269 |
| VWR_day1_dark | drug-control-N | vs | nc | 16 | 0.070 | 1.320.272 | -1.002 | 1.143 | 16 | 0.586 | 1.520.658 | -0.510 | 1.681 |
| VWR_day2_light | drug-control-N | vs | nc | 16 | 0.537 | 145.846 | -0.554 | 1.629 | 16 | 0.541 | 766.532 | -0.551 | 1.633 |
| VWR_day2_dark | drug-control-N | vs | nc | 16 | 0.501 | 1.005.943 | -0.589 | 1.590 | 16 | 0.158 | 1.687.909 | -0.916 | 1.232 |
| VWR_day3_light | drug-control-N | vs | nc | 15 | 0.320 | 349.453 | -0.805 | 1.445 | 16 | -0.571 | 61.991 | -1.665 | 0.523 |
| VWR_day3_dark | drug-control-N | vs | nc | 15 | 0.314 | 931.841 | -0.811 | 1.439 | 16 | 0.160 | 1.863.004 | -0.914 | 1.234 |
| FCM_bl-6 | drug-control-N |  | nc |  |  |  |  |  | 15 | 0.285 | 9.236 | -0.839 | 1.409 |
| FCM_bl-3 | drug-control-N | vs | nc | 16 | 0.671 | 4.468 | -0.431 | 1.774 | 16 | 0.160 | 12.941 | -0.914 | 1.234 |
| FCM_day1 | drug-control-N | vs | nc | 16 | 0.606 | 6.292 | -0.491 | 1.703 | 16 | 0.106 | 17.385 | -0.968 | 1.179 |
| FCM_day4 | drug-control-N | vs | nc | 16 | 0.754 | 4.645 | -0.356 | 1.863 | 16 | 0.762 | 15.576 | -0.349 | 1.872 |
| bwc_preOP | drug-control-NL | vs | nc | 16 | 0.193 | 1.026 | -0.882 | 1.268 | 16 | -0.019 | 1.081 | -1.091 | 1.053 |
| bwc_recovery | drug-control-NL | vs | nc | 16 | 0.239 | 0.674 | -0.838 | 1.315 | 16 | 0.586 | 0.943 | -0.509 | 1.682 |
| bwc_6h | drug-control-NL | vs | nc | 16 | 0.518 | 0.714 | -0.572 | 1.608 | 15 | 1.597 | 1.208 | 0.314 | 2.881 |
| bwc_day1 | drug-control-NL | vs | nc | 16 | -0.692 | 0.954 | -1.796 | 0.412 | 16 | -0.959 | 1.188 | -2.091 | 0.174 |
| bwc_day2 | drug-control-NL | vs | nc | 16 | -0.151 | 1.275 | -1.225 | 0.923 | 16 | -0.456 | 1.085 | -1.543 | 0.630 |
| bwc_day3 | drug-control-NL | vs | nc | 16 | -0.088 | 1.578 | -1.161 | 0.985 | 16 | 0.527 | 1.133 | -0.564 | 1.618 |
| bwc_day4 | drug-control-NL | vs | nc | 16 | -0.329 | 1.506 | -1.409 | 0.751 | 16 | -0.271 | 1.481 | -1.348 | 0.806 |
| bur_bl_2h | drug-control-NL | vs | nc | 16 | 0.346 | 11.314 | -0.735 | 1.426 | 16 | -0.490 | 56.709 | -1.578 | 0.599 |
| bur_bl_4h | drug-control-NL | vs | nc | 16 | 0.302 | 15.773 | -0.776 | 1.381 | 16 | -0.502 | 56.533 | -1.591 | 0.587 |
| bur_bl_6h | drug-control-NL | vs | nc | 16 | 0.274 | 17.104 | -0.803 | 1.352 | 16 | -0.499 | 56.573 | -1.588 | 0.590 |
| bur_bl_20h | drug-control-NL | vs | nc | 16 | 1.121 | 15.644 | -0.033 | 2.274 | 16 | -0.415 | 58.439 | -1.499 | 0.669 |
| bur_bl_lat | drug-control-NL | vs | nc | 16 | -0.434 | 538.343 | -1.519 | 0.651 | 16 | 0.500 | 315.682 | -0.589 | 1.589 |
| bur_exp_2h | drug-control-NL | vs | nc | 16 | -0.564 | 2.705 | -1.657 | 0.530 | 16 | -0.842 | 8.628 | -1.961 | 0.277 |
| bur_exp_4h | drug-control-NL | vs | nc | 16 | -0.452 | 2.750 | -1.538 | 0.634 | 16 | -0.775 | 11.657 | -1.887 | 0.337 |
| bur_exp_6h | drug-control-NL | vs | nc | 16 | 0.467 | 4.252 | -0.619 | 1.554 | 16 | -0.686 | 11.945 | -1.790 | 0.417 |
| bur_exp_20h | drug-control-NL | vs | nc | 16 | 0.139 | 17.475 | -0.935 | 1.213 | 16 | -0.246 | 18.964 | -1.323 | 0.830 |
| bur_exp_lat | drug-control-NL | vs | nc | 16 | 0.871 | 5.248.730 | -0.251 | 1.993 | 16 | 0.324 | 1.778.397 | -0.756 | 1.403 |
| liquid-intake_bl_mean/h | drug-control-NL | vs | nc | 16 | 0.021 | 0.032 | -1.052 | 1.093 | 16 | -0.028 | 0.012 | -1.101 | 1.044 |
| liquid-intake_day-1/h | drug-control-NL | vs | nc | 16 | 0.352 | 0.083 | -0.729 | 1.433 | 16 | 1.998 | 0.038 | 0.685 | 3.311 |
| liquid-intake_day0/h | drug-control-NL | vs | nc | 16 | -0.731 | 0.044 | -1.839 | 0.377 | 16 | -0.468 | 0.131 | -1.555 | 0.619 |
| liquid-intake_day1/h | drug-control-NL | vs | nc | 16 | 0.378 | 0.028 | -0.704 | 1.460 | 16 | -0.024 | 0.022 | -1.097 | 1.048 |
| liquid-intake_day2/h | drug-control-NL | vs | nc | 16 | 0.124 | 0.034 | -0.949 | 1.197 | 16 | 1.015 | 0.012 | -0.124 | 2.155 |
| liquid-intake_day3/h | drug-control-NL | vs | nc | 16 | 0.224 | 0.036 | -0.851 | 1.300 | 16 | -0.425 | 0.018 | -1.509 | 0.660 |
| MGS_bl | drug-control-NL | vs | nc | 16 | 0.223 | 0.052 | -0.852 | 1.299 | 16 | -0.060 | 0.066 | -1.133 | 1.012 |
| MGS_2h | drug-control-NL | vs | nc | 16 | 2.061 | 0.076 | 0.734 | 3.387 | 16 | 3.102 | 0.050 | 1.510 | 4.693 |
| MGS_4h | drug-control-NL | vs | nc | 16 | 1.478 | 0.082 | 0.268 | 2.688 | 16 | 1.433 | 0.085 | 0.231 | 2.635 |
| MGS_6h | drug-control-NL | vs | nc | 16 | 1.068 | 0.076 | -0.078 | 2.215 | 16 | 0.823 | 0.068 | -0.294 | 1.939 |
| MGS_8h | drug-control-NL | vs | nc | 16 | 0.791 | 0.084 | -0.323 | 1.904 | 16 | 0.341 | 0.110 | -0.739 | 1.421 |
| MGS_day1 | drug-control-NL | vs | nc | 16 | 2.309 | 0.037 | 0.925 | 3.694 | 16 | 1.061 | 0.052 | -0.084 | 2.207 |
| MGS_day2 | drug-control-NL | vs | nc | 16 | 1.827 | 0.037 | 0.550 | 3.104 | 16 | -0.461 | 0.058 | -1.547 | 0.626 |
| MGS_day3 | drug-control-NL | vs | nc | 16 | 0.059 | 0.073 | -1.014 | 1.131 | 16 | -0.563 | 0.049 | -1.657 | 0.530 |
| MGS_day4 | drug-control-NL | vs | nc | 16 | 0.073 | 0.054 | -0.999 | 1.146 | 16 | -0.156 | 0.055 | -1.230 | 0.918 |
| nest_bl_2h | drug-control-NL | vs | nc | 16 | 0.441 | 0.567 | -0.644 | 1.526 | 16 | 0.500 | 0.250 | -0.589 | 1.589 |
| nest_bl_4h | drug-control-NL | vs | nc | 16 | 0.347 | 0.541 | -0.734 | 1.427 | 16 | 0.671 | 0.280 | -0.431 | 1.773 |
| nest_bl_6h | drug-control-NL | vs | nc | 16 | 0.407 | 0.460 | -0.676 | 1.491 | 16 | 0.500 | 0.250 | -0.589 | 1.589 |
| nest_bl_day-3 | drug-control-NL | vs | nc | 16 | -0.257 | 0.730 | -1.334 | 0.820 | 16 | 0.000 | 0.259 | -1.072 | 1.072 |
| nest_exp_2h | drug-control-NL | vs | nc | 16 | -0.483 | 0.259 | -1.571 | 0.605 | 16 | -1.080 | 0.231 | -2.228 | 0.068 |
| nest_exp_4h | drug-control-NL | vs | nc | 16 | -0.483 | 0.259 | -1.571 | 0.605 | 16 | -1.080 | 0.231 | -2.228 | 0.068 |
| nest_exp_6h | drug-control-NL | vs | nc | 16 | 0.000 | 0.259 | -1.072 | 1.072 | 16 | -1.128 | 0.222 | -2.283 | 0.026 |
| nest_day1 | drug-control-NL | vs | nc | 16 | 0.332 | 0.565 | -0.748 | 1.412 | 16 | -0.212 | 0.590 | -1.287 | 0.864 |
| nest_day2 | drug-control-NL | vs | nc | 16 | -0.130 | 0.479 | -1.204 | 0.943 | 16 | 0.000 | 0.496 | -1.072 | 1.072 |
| nest_day3 | drug-control-NL | vs | nc | 16 | -0.652 | 0.479 | -1.752 | 0.449 | 16 | -0.162 | 0.387 | -1.236 | 0.913 |
| nest_day4 | drug-control-NL | vs | nc | 16 | -0.112 | 0.557 | -1.185 | 0.961 | 16 | 0.300 | 0.417 | -0.779 | 1.378 |
| distance_day0-20h | drug-control-NL | vs | nc | 16 | -0.314 | 10.169.191 | -1.393 | 0.765 | 16 | -0.382 | 6.238.842 | -1.464 | 0.700 |
| distance_day0_dark | drug-control-NL | vs | nc | 16 | -0.592 | 7.101.034 | -1.688 | 0.504 | 16 | -0.266 | 5.245.531 | -1.343 | 0.812 |
| distance_day1_light | drug-control-NL | vs | nc | 16 | -0.606 | 10.309.065 | -1.702 | 0.491 | 16 | 0.338 | 14.748.598 | -0.742 | 1.418 |
| distance_day1_dark | drug-control-NL | vs | nc | 16 | -0.074 | 18.225.908 | -1.146 | 0.999 | 16 | -0.432 | 19.009.253 | -1.517 | 0.653 |
| distance_day2_light | drug-control-NL | vs | nc | 16 | 0.908 | 5.400.632 | -0.218 | 2.034 | 16 | 0.806 | 3.817.853 | -0.309 | 1.922 |
| distance_day2_dark | drug-control-NL | vs | nc | 16 | 0.372 | 11.531.955 | -0.709 | 1.454 | 16 | -0.230 | 20.805.292 | -1.306 | 0.846 |
| distance_day3_light | drug-control-NL | vs | nc | 15 | 0.821 | 6.034.367 | -0.343 | 1.985 | 16 | 0.840 | 9.209.682 | -0.279 | 1.959 |
| distance_day3_dark | drug-control-NL | vs | nc | 15 | -0.172 | 9.164.920 | -1.292 | 0.948 | 16 | 0.135 | 19.057.581 | -0.939 | 1.208 |
| velocity_day0-20h | drug-control-NL | vs | nc | 16 | -0.256 | 0.170 | -1.333 | 0.821 | 16 | -0.254 | 0.113 | -1.331 | 0.823 |
| velocity_day0-dark | drug-control-NL | vs | nc | 16 | -0.556 | 0.206 | -1.649 | 0.537 | 16 | -0.142 | 0.137 | -1.216 | 0.931 |
| velocity_day1_light | drug-control-NL | vs | nc | 16 | -0.563 | 0.390 | -1.656 | 0.531 | 16 | 0.315 | 0.413 | -0.764 | 1.394 |
| velocity_day1_dark | drug-control-NL | vs | nc | 16 | 0.052 | 0.581 | -1.021 | 1.125 | 16 | -0.358 | 0.504 | -1.439 | 0.723 |
| velocity_day2_light | drug-control-NL | vs | nc | 16 | 0.865 | 0.153 | -0.257 | 1.986 | 16 | 0.821 | 0.107 | -0.296 | 1.937 |
| velocity_day2_dark | drug-control-NL | vs | nc | 16 | 0.365 | 0.412 | -0.717 | 1.446 | 16 | -0.169 | 0.489 | -1.243 | 0.905 |
| velocity_day3_light | drug-control-NL | vs | nc | 15 | 0.842 | 0.166 | -0.324 | 2.009 | 16 | 0.921 | 0.091 | -0.207 | 2.049 |
| velocity_day3_dark | drug-control-NL | vs | nc | 15 | -0.120 | 0.296 | -1.239 | 0.999 | 16 | 0.118 | 0.488 | -0.956 | 1.191 |
| VWR_day1_dark | drug-control-NL | vs | nc | 16 | -0.117 | 1.118.188 | -1.191 | 0.956 | 16 | 0.461 | 1.073.319 | -0.626 | 1.547 |
| VWR_day2_light | drug-control-NL | vs | nc | 16 | 0.909 | 267.156 | -0.218 | 2.035 | 16 | 0.965 | 208.641 | -0.168 | 2.098 |
| VWR_day2_dark | drug-control-NL | vs | nc | 16 | 0.369 | 1.001.850 | -0.712 | 1.451 | 16 | 0.231 | 1.337.132 | -0.845 | 1.307 |
| VWR_day3_light | drug-control-NL | vs | nc | 15 | 0.731 | 247.013 | -0.424 | 1.885 | 16 | 0.478 | 166.774 | -0.610 | 1.565 |
| VWR_day3_dark | drug-control-NL | vs | nc | 15 | 0.029 | 1.086.917 | -1.090 | 1.147 | 16 | 0.307 | 1.538.344 | -0.772 | 1.386 |
| FCM_bl-6 | drug-control-NL |  | nc |  |  |  |  |  | 16 | 0.110 | 7.226 | -0.963 | 1.183 |
| FCM_bl-3 | drug-control-NL | vs | nc | 16 | 0.581 | 8.721 | -0.514 | 1.675 | 16 | -0.012 | 14.823 | -1.084 | 1.061 |
| FCM_day1 | drug-control-NL | vs | nc | 16 | -0.021 | 5.939 | -1.093 | 1.051 | 16 | -0.428 | 17.902 | -1.513 | 0.656 |
| FCM_day4 | drug-control-NL | vs | nc | 16 | 0.819 | 3.969 | -0.298 | 1.935 | 16 | 0.967 | 13.648 | -0.167 | 2.100 |
| bwc_preOP | drug-control-NO | vs | nc | 16 | 0.345 | 1.135 | -0.736 | 1.425 | 16 | 0.312 | 1.046 | -0.767 | 1.391 |
| bwc_recovery | drug-control-NO | vs | nc | 16 | 0.578 | 0.870 | -0.517 | 1.672 | 16 | 0.338 | 1.037 | -0.742 | 1.418 |
| bwc_6h | drug-control-NO | vs | nc | 16 | 0.277 | 1.167 | -0.801 | 1.355 | 16 | 0.881 | 0.866 | -0.242 | 2.004 |
| bwc_day1 | drug-control-NO | vs | nc | 16 | -0.596 | 1.149 | -1.692 | 0.500 | 16 | -0.912 | 0.915 | -2.039 | 0.215 |
| bwc_day2 | drug-control-NO | vs | nc | 16 | -0.163 | 1.133 | -1.237 | 0.912 | 16 | -1.115 | 1.068 | -2.267 | 0.038 |
| bwc_day3 | drug-control-NO | vs | nc | 16 | 0.144 | 1.184 | -0.930 | 1.218 | 16 | 0.053 | 1.590 | -1.020 | 1.126 |
| bwc_day4 | drug-control-NO | vs | nc | 16 | -0.331 | 1.127 | -1.411 | 0.748 | 16 | 0.085 | 2.146 | -0.988 | 1.158 |
| bur_bl_2h | drug-control-NO | vs | nc | 16 | -0.012 | 7.041 | -1.084 | 1.061 | 16 | -0.706 | 55.274 | -1.811 | 0.399 |
| bur_bl_4h | drug-control-NO | vs | nc | 16 | -0.199 | 8.656 | -1.274 | 0.876 | 16 | -0.719 | 55.068 | -1.826 | 0.387 |
| bur_bl_6h | drug-control-NO | vs | nc | 16 | -0.240 | 10.661 | -1.317 | 0.836 | 16 | -0.723 | 55.113 | -1.830 | 0.384 |
| bur_bl_20h | drug-control-NO | vs | nc | 16 | 0.295 | 16.195 | -0.783 | 1.373 | 16 | -0.835 | 56.187 | -1.953 | 0.283 |
| bur_bl_lat | drug-control-NO | vs | nc | 16 | -1.071 | 440.721 | -2.218 | 0.076 | 16 | 0.391 | 224.253 | -0.692 | 1.474 |
| bur_exp_2h | drug-control-NO | vs | nc | 16 | -0.775 | 2.475 | -1.887 | 0.337 | 16 | -0.726 | 8.714 | -1.833 | 0.381 |
| bur_exp_4h | drug-control-NO | vs | nc | 16 | -0.730 | 2.459 | -1.837 | 0.378 | 16 | -0.534 | 12.178 | -1.626 | 0.557 |
| bur_exp_6h | drug-control-NO | vs | nc | 16 | -1.111 | 2.510 | -2.263 | 0.041 | 16 | -0.430 | 12.597 | -1.514 | 0.655 |
| bur_exp_20h | drug-control-NO | vs | nc | 16 | -0.353 | 15.129 | -1.434 | 0.728 | 16 | -0.074 | 19.902 | -1.147 | 0.999 |
| bur_exp_lat | drug-control-NO | vs | nc | 15 | 2.273 | 1.347.117 | 0.840 | 3.706 | 16 | 0.908 | 1.238.373 | -0.219 | 2.034 |
| liquid-intake_bl_mean/h | drug-control-NO | vs | nc | 16 | -0.039 | 0.031 | -1.112 | 1.033 | 16 | -0.136 | 0.009 | -1.209 | 0.938 |
| liquid-intake_day-1/h | drug-control-NO | vs | nc | 16 | 0.427 | 0.073 | -0.657 | 1.512 | 16 | 2.582 | 0.038 | 1.130 | 4.034 |
| liquid-intake_day0/h | drug-control-NO | vs | nc | 16 | 0.080 | 0.043 | -0.993 | 1.153 | 16 | -0.552 | 0.488 | -1.645 | 0.541 |
| liquid-intake_day1/h | drug-control-NO | vs | nc | 16 | 0.758 | 0.026 | -0.352 | 1.868 | 16 | 0.310 | 0.018 | -0.769 | 1.389 |
| liquid-intake_day2/h | drug-control-NO | vs | nc | 16 | 0.335 | 0.028 | -0.744 | 1.415 | 16 | 0.254 | 0.018 | -0.822 | 1.331 |
| liquid-intake_day3/h | drug-control-NO | vs | nc | 16 | -0.011 | 0.023 | -1.083 | 1.061 | 16 | -0.665 | 0.012 | -1.767 | 0.436 |
| MGS_bl | drug-control-NO | vs | nc | 16 | -0.344 | 0.045 | -1.425 | 0.736 | 16 | -0.085 | 0.048 | -1.158 | 0.988 |
| MGS_2h | drug-control-NO | vs | nc | 16 | 1.898 | 0.074 | 0.606 | 3.189 | 16 | 3.117 | 0.063 | 1.521 | 4.713 |
| MGS_4h | drug-control-NO | vs | nc | 16 | 1.307 | 0.088 | 0.126 | 2.489 | 16 | 1.490 | 0.082 | 0.278 | 2.702 |
| MGS_6h | drug-control-NO | vs | nc | 16 | 1.542 | 0.075 | 0.321 | 2.763 | 16 | 0.764 | 0.074 | -0.347 | 1.875 |
| MGS_8h | drug-control-NO | vs | nc | 16 | 0.865 | 0.106 | -0.256 | 1.986 | 16 | 0.893 | 0.112 | -0.232 | 2.018 |
| MGS_day1 | drug-control-NO | vs | nc | 16 | 1.582 | 0.046 | 0.354 | 2.811 | 16 | 0.916 | 0.046 | -0.211 | 2.044 |
| MGS_day2 | drug-control-NO | vs | nc | 16 | 0.937 | 0.036 | -0.192 | 2.067 | 16 | -1.171 | 0.040 | -2.332 | -0.010 |
| MGS_day3 | drug-control-NO | vs | nc | 16 | -0.551 | 0.066 | -1.644 | 0.541 | 16 | -0.431 | 0.056 | -1.516 | 0.654 |
| MGS_day4 | drug-control-NO | vs | nc | 16 | 0.254 | 0.048 | -0.823 | 1.330 | 16 | -0.276 | 0.055 | -1.354 | 0.801 |
| nest_bl_2h | drug-control-NO | vs | nc | 16 | 0.323 | 0.581 | -0.756 | 1.402 | 16 | 0.000 | 0.231 | -1.072 | 1.072 |
| nest_bl_4h | drug-control-NO | vs | nc | 16 | 0.116 | 0.541 | -0.958 | 1.189 | 16 | 0.303 | 0.206 | -0.775 | 1.382 |
| nest_bl_6h | drug-control-NO | vs | nc | 16 | 0.128 | 0.489 | -0.946 | 1.201 | 16 | 0.303 | 0.206 | -0.775 | 1.382 |
| nest_bl_day-3 | drug-control-NO | vs | nc | 16 | 0.000 | 0.756 | -1.072 | 1.072 | 16 | 0.238 | 0.263 | -0.839 | 1.314 |
| nest_exp_2h | drug-control-NO | vs | nc | 16 | -1.708 | 0.183 | -2.961 | -0.455 | 16 | -0.500 | 0.250 | -1.589 | 0.589 |
| nest_exp_4h | drug-control-NO | vs | nc | 16 | -1.708 | 0.183 | -2.961 | -0.455 | 16 | -0.255 | 0.245 | -1.331 | 0.822 |
| nest_exp_6h | drug-control-NO | vs | nc | 16 | -0.764 | 0.245 | -1.875 | 0.347 | 16 | -0.303 | 0.206 | -1.382 | 0.775 |
| nest_day1 | drug-control-NO | vs | nc | 15 | -1.030 | 0.515 | -2.220 | 0.159 | 16 | 0.364 | 0.515 | -0.717 | 1.445 |
| nest_day2 | drug-control-NO | vs | nc | 16 | -0.370 | 0.507 | -1.452 | 0.711 | 16 | 0.000 | 0.417 | -1.072 | 1.072 |
| nest_day3 | drug-control-NO | vs | nc | 16 | -0.500 | 0.500 | -1.589 | 0.589 | 16 | 0.342 | 0.366 | -0.739 | 1.422 |
| nest_day4 | drug-control-NO | vs | nc | 16 | -0.128 | 0.489 | -1.201 | 0.946 | 16 | 1.080 | 0.347 | -0.068 | 2.228 |
| distance_day0-20h | drug-control-NO | vs | nc | 16 | -0.344 | 10.222.245 | -1.424 | 0.737 | 16 | 0.342 | 5.948.754 | -0.738 | 1.423 |
| distance_day0_dark | drug-control-NO | vs | nc | 16 | -0.953 | 7.144.021 | -2.084 | 0.179 | 16 | -0.588 | 4.872.984 | -1.684 | 0.507 |
| distance_day1_light | drug-control-NO | vs | nc | 16 | -0.777 | 9.160.069 | -1.889 | 0.336 | 16 | -0.557 | 11.436.489 | -1.650 | 0.536 |
| distance_day1_dark | drug-control-NO | vs | nc | 16 | -0.880 | 11.510.620 | -2.004 | 0.243 | 16 | -0.284 | 20.440.842 | -1.361 | 0.794 |
| distance_day2_light | drug-control-NO | vs | nc | 16 | 0.447 | 2.634.462 | -0.639 | 1.533 | 16 | 0.548 | 18.316.096 | -0.544 | 1.640 |
| distance_day2_dark | drug-control-NO | vs | nc | 16 | 0.455 | 15.845.230 | -0.631 | 1.542 | 16 | 0.230 | 25.765.098 | -0.846 | 1.306 |
| distance_day3_light | drug-control-NO | vs | nc | 15 | 0.674 | 4.477.600 | -0.475 | 1.824 | 16 | 0.558 | 13.676.308 | -0.536 | 1.651 |
| distance_day3_dark | drug-control-NO | vs | nc | 15 | 0.399 | 14.066.218 | -0.730 | 1.528 | 16 | 0.249 | 22.923.694 | -0.828 | 1.325 |
| velocity_day0-20h | drug-control-NO | vs | nc | 16 | -0.182 | 0.171 | -1.257 | 0.892 | 16 | 0.474 | 0.108 | -0.613 | 1.562 |
| velocity_day0-dark | drug-control-NO | vs | nc | 16 | -0.880 | 0.211 | -2.003 | 0.243 | 16 | -0.637 | 0.120 | -1.736 | 0.462 |
| velocity_day1_light | drug-control-NO | vs | nc | 16 | -0.833 | 0.350 | -1.951 | 0.285 | 16 | -0.548 | 0.340 | -1.640 | 0.545 |
| velocity_day1_dark | drug-control-NO | vs | nc | 16 | -0.889 | 0.306 | -2.013 | 0.235 | 16 | -0.351 | 0.512 | -1.431 | 0.730 |
| velocity_day2_light | drug-control-NO | vs | nc | 16 | 0.303 | 0.074 | -0.776 | 1.381 | 16 | 0.540 | 0.554 | -0.552 | 1.631 |
| velocity_day2_dark | drug-control-NO | vs | nc | 16 | 0.322 | 0.398 | -0.757 | 1.402 | 16 | 0.195 | 0.627 | -0.879 | 1.270 |
| velocity_day3_light | drug-control-NO | vs | nc | 15 | 0.849 | 0.144 | -0.318 | 2.016 | 16 | 0.542 | 0.464 | -0.550 | 1.633 |
| velocity_day3_dark | drug-control-NO | vs | nc | 15 | 0.194 | 0.340 | -0.926 | 1.315 | 16 | 0.223 | 0.593 | -0.853 | 1.299 |
| VWR_day1_dark | drug-control-NO | vs | nc | 16 | -0.777 | 857.980 | -1.889 | 0.335 | 16 | -0.461 | 997.741 | -1.548 | 0.625 |
| VWR_day2_light | drug-control-NO | vs | nc | 16 | 0.400 | 95.845 | -0.683 | 1.483 | 16 | 0.508 | 44.479 | -0.582 | 1.597 |
| VWR_day2_dark | drug-control-NO | vs | nc | 16 | 0.258 | 907.797 | -0.819 | 1.335 | 16 | -0.046 | 1.468.337 | -1.118 | 1.027 |
| VWR_day3_light | drug-control-NO | vs | nc | 15 | 0.464 | 244.577 | -0.669 | 1.597 | 16 | 0.283 | 146.631 | -0.795 | 1.361 |
| VWR_day3_dark | drug-control-NO | vs | nc | 15 | 0.231 | 843.421 | -0.891 | 1.352 | 16 | 0.320 | 1.557.561 | -0.759 | 1.400 |
| FCM_bl-6 | drug-control-NO |  | nc |  |  |  |  |  | 16 | 0.370 | 8.872 | -0.712 | 1.451 |
| FCM_bl-3 | drug-control-NO | vs | nc | 16 | 0.027 | 4.659 | -1.046 | 1.099 | 16 | -0.142 | 11.820 | -1.216 | 0.932 |
| FCM_day1 | drug-control-NO | vs | nc | 15 | 0.444 | 7.722 | -0.688 | 1.575 | 16 | 0.736 | 17.234 | -0.373 | 1.844 |
| FCM_day4 | drug-control-NO | vs | nc | 16 | 0.910 | 7.969 | -0.217 | 2.036 | 16 | 0.937 | 17.297 | -0.192 | 2.067 |
| bwc_preOP | drug-control-NLO | vs | nc | 16 | 0.623 | 1.058 | -0.475 | 1.721 | 16 | 0.164 | 1.020 | -0.910 | 1.239 |
| bwc_recovery | drug-control-NLO | vs | nc | 16 | 0.822 | 0.945 | -0.294 | 1.939 | 16 | 0.546 | 1.029 | -0.547 | 1.638 |
| bwc_6h | drug-control-NLO | vs | nc | 16 | 0.466 | 0.904 | -0.621 | 1.552 | 16 | 0.691 | 0.890 | -0.413 | 1.794 |
| bwc_day1 | drug-control-NLO | vs | nc | 16 | -0.464 | 0.929 | -1.551 | 0.622 | 16 | -1.217 | 1.006 | -2.385 | -0.050 |
| bwc_day2 | drug-control-NLO | vs | nc | 16 | -0.072 | 1.243 | -1.145 | 1.000 | 16 | -0.364 | 1.315 | -1.445 | 0.718 |
| bwc_day3 | drug-control-NLO | vs | nc | 16 | 0.338 | 1.116 | -0.742 | 1.418 | 16 | 0.658 | 1.008 | -0.443 | 1.759 |
| bwc_day4 | drug-control-NLO | vs | nc | 16 | -0.216 | 1.270 | -1.291 | 0.860 | 16 | 0.101 | 1.384 | -0.972 | 1.174 |
| bur_bl_2h | drug-control-NLO | vs | nc | 16 | -0.331 | 5.936 | -1.410 | 0.749 | 16 | -0.743 | 55.216 | -1.852 | 0.366 |
| bur_bl_4h | drug-control-NLO | vs | nc | 16 | -0.542 | 7.382 | -1.634 | 0.550 | 16 | -0.738 | 55.047 | -1.847 | 0.370 |
| bur_bl_6h | drug-control-NLO | vs | nc | 16 | -0.547 | 9.556 | -1.639 | 0.545 | 16 | -0.735 | 55.114 | -1.843 | 0.373 |
| bur_bl_20h | drug-control-NLO | vs | nc | 16 | 0.315 | 12.821 | -0.764 | 1.394 | 16 | -0.832 | 55.843 | -1.950 | 0.286 |
| bur_bl_lat | drug-control-NLO | vs | nc | 16 | 0.339 | 3.659.627 | -0.742 | 1.419 | 16 | 0.632 | 175.666 | -0.467 | 1.730 |
| bur_exp_2h | drug-control-NLO | vs | nc | 16 | -0.701 | 2.495 | -1.806 | 0.403 | 16 | -0.682 | 8.949 | -1.785 | 0.421 |
| bur_exp_4h | drug-control-NLO | vs | nc | 16 | -0.397 | 2.642 | -1.480 | 0.686 | 16 | -0.448 | 12.663 | -1.534 | 0.638 |
| bur_exp_6h | drug-control-NLO | vs | nc | 16 | 0.019 | 3.925 | -1.053 | 1.092 | 16 | -0.292 | 12.951 | -1.371 | 0.786 |
| bur_exp_20h | drug-control-NLO | vs | nc | 16 | -0.022 | 19.029 | -1.094 | 1.051 | 16 | -0.074 | 23.821 | -1.147 | 0.999 |
| bur_exp_lat | drug-control-NLO | vs | nc | 16 | 1.017 | 8.231.536 | -0.123 | 2.157 | 16 | 1.163 | 1.238.468 | 0.004 | 2.323 |
| liquid-intake_bl_mean/h | drug-control-NLO | vs | nc | 16 | 0.191 | 0.036 | -0.884 | 1.266 | 16 | -0.964 | 0.009 | -2.097 | 0.169 |
| liquid-intake_day-1/h | drug-control-NLO | vs | nc | 16 | 0.758 | 0.078 | -0.352 | 1.869 | 16 | 2.148 | 0.041 | 0.802 | 3.495 |
| liquid-intake_day0/h | drug-control-NLO | vs | nc | 16 | 0.194 | 0.081 | -0.881 | 1.269 | 16 | -0.359 | 0.097 | -1.440 | 0.722 |
| liquid-intake_day1/h | drug-control-NLO | vs | nc | 16 | 0.739 | 0.023 | -0.369 | 1.847 | 16 | 0.219 | 0.018 | -0.857 | 1.294 |
| liquid-intake_day2/h | drug-control-NLO | vs | nc | 16 | 0.422 | 0.030 | -0.662 | 1.506 | 16 | 0.950 | 0.010 | -0.181 | 2.081 |
| liquid-intake_day3/h | drug-control-NLO | vs | nc | 16 | 0.432 | 0.028 | -0.652 | 1.517 | 16 | -1.067 | 0.012 | -2.213 | 0.080 |
| MGS_bl | drug-control-NLO | vs | nc | 16 | 0.116 | 0.046 | -0.957 | 1.190 | 16 | -0.007 | 0.053 | -1.079 | 1.066 |
| MGS_2h | drug-control-NLO | vs | nc | 16 | 2.368 | 0.056 | 0.969 | 3.766 | 16 | 3.006 | 0.066 | 1.441 | 4.571 |
| MGS_4h | drug-control-NLO | vs | nc | 16 | 2.757 | 0.067 | 1.260 | 4.255 | 16 | 2.100 | 0.060 | 0.765 | 3.436 |
| MGS_6h | drug-control-NLO | vs | nc | 16 | 2.283 | 0.081 | 0.905 | 3.661 | 16 | 1.218 | 0.084 | 0.050 | 2.386 |
| MGS_8h | drug-control-NLO | vs | nc | 16 | 1.244 | 0.104 | 0.072 | 2.415 | 16 | 1.255 | 0.089 | 0.081 | 2.428 |
| MGS_day1 | drug-control-NLO | vs | nc | 16 | 1.505 | 0.073 | 0.290 | 2.720 | 16 | 0.277 | 0.048 | -0.801 | 1.354 |
| MGS_day2 | drug-control-NLO | vs | nc | 16 | 1.668 | 0.044 | 0.423 | 2.913 | 16 | 0.116 | 0.039 | -0.957 | 1.190 |
| MGS_day3 | drug-control-NLO | vs | nc | 16 | -0.022 | 0.072 | -1.095 | 1.050 | 16 | -0.488 | 0.066 | -1.576 | 0.600 |
| MGS_day4 | drug-control-NLO | vs | nc | 16 | 0.596 | 0.062 | -0.500 | 1.692 | 16 | -0.527 | 0.051 | -1.618 | 0.563 |
| nest_bl_2h | drug-control-NLO | vs | nc | 16 | 0.212 | 0.590 | -0.864 | 1.287 | 16 | -0.255 | 0.245 | -1.331 | 0.822 |
| nest_bl_4h | drug-control-NLO | vs | nc | 16 | 0.284 | 0.660 | -0.794 | 1.362 | 16 | 0.000 | 0.231 | -1.072 | 1.072 |
| nest_bl_6h | drug-control-NLO | vs | nc | 16 | 0.332 | 0.565 | -0.748 | 1.412 | 16 | 0.000 | 0.231 | -1.072 | 1.072 |
| nest_bl_day-3 | drug-control-NLO | vs | nc | 16 | -0.257 | 0.730 | -1.334 | 0.820 | 16 | -0.255 | 0.245 | -1.331 | 0.822 |
| nest_exp_2h | drug-control-NLO | vs | nc | 16 | -0.764 | 0.245 | -1.875 | 0.347 | 16 | -1.517 | 0.206 | -2.734 | -0.300 |
| nest_exp_4h | drug-control-NLO | vs | nc | 16 | -0.764 | 0.245 | -1.875 | 0.347 | 16 | -1.080 | 0.231 | -2.228 | 0.068 |
| nest_exp_6h | drug-control-NLO | vs | nc | 16 | -0.483 | 0.259 | -1.571 | 0.605 | 16 | -0.303 | 0.206 | -1.382 | 0.775 |
| nest_day1 | drug-control-NLO | vs | nc | 16 | -0.166 | 0.753 | -1.240 | 0.908 | 16 | -0.364 | 0.515 | -1.445 | 0.717 |
| nest_day2 | drug-control-NLO | vs | nc | 16 | 0.000 | 0.594 | -1.072 | 1.072 | 16 | 0.121 | 0.515 | -0.952 | 1.195 |
| nest_day3 | drug-control-NLO | vs | nc | 16 | -0.238 | 0.526 | -1.314 | 0.839 | 16 | 0.517 | 0.363 | -0.573 | 1.607 |
| nest_day4 | drug-control-NLO | vs | nc | 16 | -0.244 | 0.513 | -1.320 | 0.833 | 16 | 0.892 | 0.350 | -0.233 | 2.016 |
| distance_day0-20h | drug-control-NLO | vs | nc | 16 | 0.469 | 10.907.500 | -0.618 | 1.556 | 16 | -0.081 | 7.426.893 | -1.153 | 0.992 |
| distance_day0_dark | drug-control-NLO | vs | nc | 16 | -0.214 | 6.987.161 | -1.289 | 0.862 | 16 | -0.543 | 5.969.900 | -1.635 | 0.549 |
| distance_day1_light | drug-control-NLO | vs | nc | 16 | -1.018 | 8.732.660 | -2.158 | 0.122 | 16 | -0.400 | 11.446.896 | -1.483 | 0.683 |
| distance_day1_dark | drug-control-NLO | vs | nc | 16 | -0.620 | 11.606.721 | -1.717 | 0.478 | 16 | 0.082 | 34.553.323 | -0.991 | 1.155 |
| distance_day2_light | drug-control-NLO | vs | nc | 16 | 1.085 | 3.612.435 | -0.064 | 2.234 | 16 | 1.011 | 2.455.177 | -0.128 | 2.150 |
| distance_day2_dark | drug-control-NLO | vs | nc | 16 | 1.277 | 12.745.164 | 0.100 | 2.453 | 16 | 0.379 | 29.226.289 | -0.703 | 1.461 |
| distance_day3_light | drug-control-NLO | vs | nc | 15 | 0.409 | 4.124.901 | -0.721 | 1.538 | 16 | 0.762 | 2.853.713 | -0.348 | 1.873 |
| distance_day3_dark | drug-control-NLO | vs | nc | 15 | 1.179 | 15.598.083 | -0.032 | 2.390 | 16 | 0.523 | 21.128.982 | -0.567 | 1.614 |
| velocity_day0-20h | drug-control-NLO | vs | nc | 16 | 0.471 | 0.164 | -0.616 | 1.558 | 16 | 0.158 | 0.120 | -0.916 | 1.232 |
| velocity_day0-dark | drug-control-NLO | vs | nc | 16 | -0.319 | 0.201 | -1.398 | 0.760 | 16 | -0.382 | 0.157 | -1.464 | 0.700 |
| velocity_day1_light | drug-control-NLO | vs | nc | 16 | -1.083 | 0.339 | -2.231 | 0.065 | 16 | -0.450 | 0.338 | -1.536 | 0.636 |
| velocity_day1_dark | drug-control-NLO | vs | nc | 16 | -0.681 | 0.309 | -1.784 | 0.422 | 16 | 0.032 | 0.875 | -1.041 | 1.104 |
| velocity_day2_light | drug-control-NLO | vs | nc | 16 | 0.994 | 0.097 | -0.143 | 2.131 | 16 | 0.982 | 0.072 | -0.153 | 2.117 |
| velocity_day2_dark | drug-control-NLO | vs | nc | 16 | 1.039 | 0.332 | -0.103 | 2.182 | 16 | 0.401 | 0.807 | -0.682 | 1.484 |
| velocity_day3_light | drug-control-NLO | vs | nc | 15 | 0.397 | 0.126 | -0.732 | 1.526 | 16 | 0.732 | 0.119 | -0.376 | 1.840 |
| velocity_day3_dark | drug-control-NLO | vs | nc | 15 | 0.966 | 0.391 | -0.215 | 2.147 | 16 | 0.477 | 0.596 | -0.611 | 1.564 |
| VWR_day1_dark | drug-control-NLO | vs | nc | 16 | -0.840 | 834.328 | -1.958 | 0.279 | 16 | 0.186 | 1.573.876 | -0.889 | 1.260 |
| VWR_day2_light | drug-control-NLO | vs | nc | 16 | 0.641 | 123.707 | -0.459 | 1.740 | 16 | 0.767 | 236.727 | -0.344 | 1.878 |
| VWR_day2_dark | drug-control-NLO | vs | nc | 16 | 0.530 | 780.194 | -0.561 | 1.621 | 16 | 0.378 | 1.597.767 | -0.704 | 1.459 |
| VWR_day3_light | drug-control-NLO | vs | nc | 15 | -0.432 | 114.777 | -1.563 | 0.699 | 16 | 0.090 | 71.376 | -0.983 | 1.163 |
| VWR_day3_dark | drug-control-NLO | vs | nc | 15 | 0.417 | 1.136.347 | -0.713 | 1.547 | 16 | 0.412 | 1.728.075 | -0.672 | 1.496 |
| FCM_bl-6 | drug-control-NLO |  | nc |  |  |  |  |  | 16 | 0.599 | 13.320 | -0.498 | 1.695 |
| FCM_bl-3 | drug-control-NLO | vs | nc | 16 | 0.270 | 4.403 | -0.808 | 1.347 | 16 | 0.267 | 25.893 | -0.810 | 1.345 |
| FCM_day1 | drug-control-NLO | vs | nc | 16 | 0.363 | 8.962 | -0.719 | 1.444 | 16 | 1.121 | 23.770 | -0.033 | 2.274 |
| FCM_day4 | drug-control-NLO | vs | nc | 16 | 0.563 | 3.218 | -0.530 | 1.657 | 16 | 0.518 | 13.067 | -0.572 | 1.608 |

See table S12 for explanation of parameters. bl = measured at baseline time point (day -4 to day -3), exp = measured during experimental phase (day 0 – day 4), OP = operation, nc = naive-control group, n = animal number, d = cohen´s d, se = standard error, lwr = lower and upr = upper d of the 95 % confindence interval, vs = versus.

**Supplementary table 18: Details on Neuro score parameters**

| **Parameter** | **Scoring range** | **Description** |
| --- | --- | --- |
| **Observation in the home cage** | | |
| **Body position** | [-2] – [2] | Flat, lying on the side or on the abdomen, no muscle tone / Reduced muscle tone, occasional postural corrections / Normal posture / Rigid or cramped posture (e.g., opisthotonus) / Animal shows no resting position, evaluation not possible |
| **Pelvic elevation** | [-1] – [1] | Flattened pelvic position/ Normal pelvic position / Elevated pelvic position |
| **Tail elevation** | [-1] – [2] | Tail flat on the floor while walking / Normal tail position / Tail erected (≤ 90 ° angle) during resting and activity period, tail rattling / Tail erected (≥ 90 ° angle, Straub phenomenon) |
| **Limb rotation** | [0] – [2] | Not present / Slight lateral rotation / Pronounced lateral rotation |
| **Locomotor activity** | [-2] – [2] | No activity / Reduced activity / Normal activity /Slight to moderate increase in activity / Pronounced increase in activity with restless motion type |
| **Ptosis**  (cave: differentiate from orbital tightening of MGS) | [0] – [2] | Eyelids opened normally / Eyelids half opened / Eyelids closed |
| **Exophthalmia** | [0] – [2] | No exophthalmia / Mild exophthalmia / Pronounced exophthalmia |
| **Lacrimation** | [0] / [2] | Not present / Present |
| **Hypersalivation** | [0] / [1] | Not present / Present |
| **Respiratory Rate** | [-2] – [2] | Acute respiratory distress, severe dyspnoea / Reduced and/or irregular breathing with abdominal breathing / Normal breathing / Slight increase in breathing rate / Pronounced increase in breathing rate (also present during resting periods) |
| **Piloerection** | [0] / [2] | Not present / Present |
| **Skin perfusion** | [-2] – [2] | Cyanosis (bluish discoloration of tail and paws) / Pale skin (e.g. paws or tail)/ Normal skin perfusion / Mild to moderate erythema (paws and tail dark pink in color) / Pronounced erythema (skin, paws, or tail red in color) |
| **Ataxia** | [0] – [2] | Not present / Slight to moderate ataxia (unsteady gait with swaying and occasional stumbling) / Pronounced ataxia (from frequent stumbling to inability to move) |
| **Stereotypies**  (e.g. walking or running in circles, constant biting of the cage grid) | [0] / [2] | Not present / Present |
| **Freezing** | [0] – [2] | Not present / Transient short episodes of freezing behavior/ Long-lasting freezing behavior |
| **Tremors**  (instinctive, repetitive trembling of muscle groups) | [0] / [2] | Not present / Present |
| **Twitches**  (instinctive or specific, non-repetitive) | [0] / [2] | Not present / Present |
| **Convulsions** | [0] / [2] | Not present / Present |
| **Vocalisation**  (not when fixated) | [0] – [2] | Not present / Occasional vocalisation with low volume/ Frequent, louder vocalisation |
| **Feces** | [-1] – [2] | No or scarce amount of feces in the cage / Character and amount of feces normal / Increased amount of feces / Diarrhea (liquid feces) |
| **Implant area** | [0] – [2] | Normal wound healing of implant area / Minor inflammation in the implant area / Pronounced inflammation in the implant area and/or necrotic areas (with qualitative description of changes in the protocols) |
| **Observations with cage lid removed** | | |
| **Curiosity**  (reaction towards presenting an object (pen)) | [-2] – [2] | No reaction / Decreased curiosity / Normal interest towards the presented object / Intensified tracking or chasing of the presented object when retracting / Attack behavior towards the presented object |
| **Startle** | [-2] – [2] | No reaction / Delayed or reduced reaction / Normal reaction/ Intensified escape reaction/ Pronounced reaction (e.g. eyelids closed, ears pulled back, pressing on the cage floor or freezing) |
| **Touch reaction** | [-2] – [2] | No reaction / Slow, reduced reaction / Normal response to light touch / Intensified escape or transient freezing reaction / Pronounced escape reaction or longer lasting freezing reaction |
| **Observations while handling the mouse** | | |
| **Vocalisation** | [0] / [1] | Not present / Present |
| **Irritability**  (e.g. bite attempt) | [0] – [2] | Not present / Mild irritability / Pronounced irritability |
| **Body tone** | [-2] – [2] | Flaccid muscle tone / Reduced muscle tone / Normal muscle tone / Increased muscle tone / Rigid muscle tone |
| **Abdominal tone** | [-1] – [1] | Reduced abdominal muscle tone / Normal abdominal muscle tone / Increased abdominal muscle tone |
| **Urination** | [0] / [1] | Not present / Present |
| **Defecation** | [0] / [1] | Not present / Present |
